# Bridging the gap: co-producing resources for NHS mental health trusts and community providers to support people with severe mental illness live active lives in their community

**DOI:** 10.1186/s12889-026-27936-7

**Published:** 2026-06-16

**Authors:** H Quirk, T Burke, G Jones, L Fletcher, M Faires, E Hillison, M Horspool, S Ker, K Machaczek, E. Peckham

**Affiliations:** 1 School of Medicine and Population Health, The University of Sheffield, Sheffield, UK; 2 Centre for Mental Health and Society, School of Health Sciences, Bangor University, Bangor, UK; 3 Advanced Wellbeing Research Centre, Sheffield Hallam University, Sheffield, UK; 4 Sport for Confidence, Patch Chelmsford, Chelmsford, UK; 5 Institute for Health and Care Improvement, York St John University, Lord Mayor’s Walk, York, UK; 6 Sheffield Health Partnership University NHS Foundation Trust, Centre Court, Atlas Way, Sheffield, UK; 7 Tees, Esk and Wear Valleys NHS Foundation Trust, Darlington, UK; 8 Centre for Applied and Social Care Research and Advanced Wellbeing Research Centre, Sheffield Hallam University, Sheffield, UK

**Keywords:** Exercise, Severe mental illness, Health behaviour, Co-produced resources, Community-based physical activity, Community-based activity providers, Schizophrenia, Bipolar disorder, Psychosis

## Abstract

**Background:**

Life expectancy is reduced by 15–20 years for people with severe mental illness (SMI). Many of these deaths result from preventable physical health conditions linked to lack of physical activity. During a supervised physical activity intervention delivered in the community by NHS staff, participants and advisors emphasised the crucial role of community-based physical activity providers, in helping people with SMI maintain their physical activity post intervention. However, community-based physical activity providers often lack guidance or support to engage people with SMI, which may hinder physical activity maintenance for this population. The aim of this project was to co-produce support resources for community-based physical activity providers and NHS professionals, to enhance physical activity engagement among people living with SMI.

**Methods:**

Support resources were developed using the Double Diamond design framework. ‘Data was collected through 32 in-depth, semi-structured interviews with NHS staff trained to deliver a physical activity intervention and community-based activity providers, alongside a national survey of 52 community-based physical activity providers. The results of these informed three co-production workshops involving people with lived experience of SMI.

**Results:**

Interviews and survey findings highlighted community providers’ willingness to be inclusive, alongside their limited confidence, knowledge, and procedural guidance for supporting people with SMI. These insights informed the co-production of two resources: a practical support booklet and a lived-experience video.

**Conclusions:**

We co-produced resources to support community-based physical activity providers in engaging people with SMI. These resources are expected to enhance the impact of physical activity interventions bridging the gap between the NHS and community providers and, when implemented in community settings, improve providers’ capacity to create safe and inclusive spaces for physical activity.

**Supplementary Information:**

The online version contains supplementary material available at 10.1186/s12889-026-27936-7.

## Background

People living with severe mental illness (SMI) such as schizophrenia, bipolar disorder and schizoaffective disorder experience a mortality gap of 15–20 years compared to people without SMI [1]. An important contributing factor to this is preventable physical health conditions such as cardiovascular disease and diabetes [2]. Life expectancy for people with SMI has declined over recent decades [3] and addressing this widening health inequality is a priority for the NHS [4].

Previous international [3] and UK research [5] has demonstrated that people with SMI engage in substantially lower physical activity behaviour and higher sedentary behaviour than those without SMI. Additionally, research has shown that people with SMI experience unique barriers to engaging in physical activity such as increased symptoms of mental illness, lack of social support and the side effects of medication [6]. These barriers highlight the importance of interventions that provide both structure and social support, which a meta-ethnography has shown can be achieved through community-based group physical activity [7, 8].

**Fig. 1:**
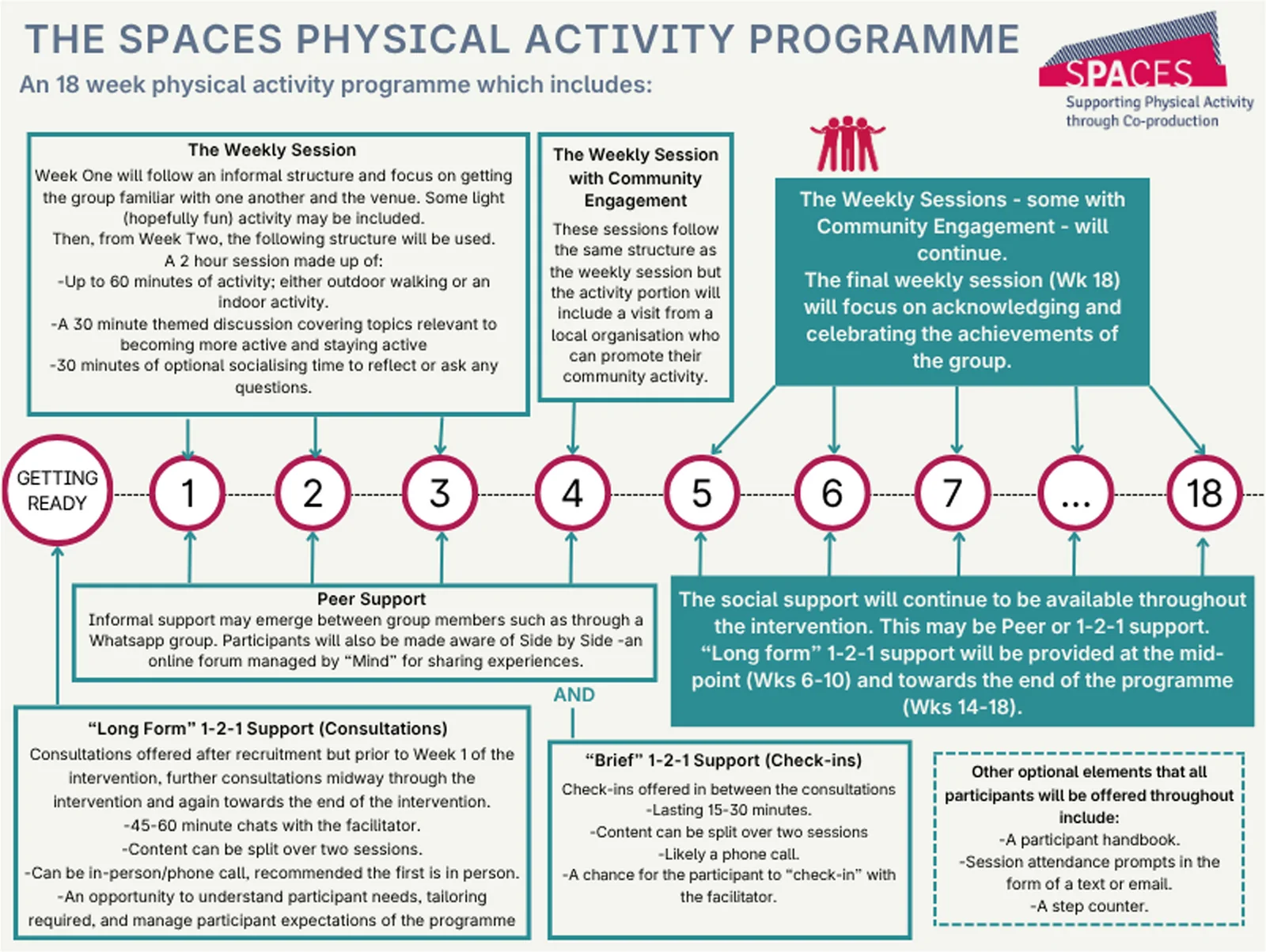
Infographic of the SPACES intervention

The Supporting Physical Activity through Co-production in pEople with Severe mental illness (SPACES) programme was funded in 2021 by the National Institute for Health and Care Research’s (NIHR) Programme Grants for Applied Research (PGfAR) and aimed to address some of these unique barriers to physical activity for people with SMI [7, 9]. The aim of SPACES was to co-produce and evaluate an intervention delivered by the NHS aimed at increasing physical activity in people with SMI. The intervention developed is an 18-week, weekly group-based physical activity programme led by NHS professionals within a community setting; trained by the SPACES research team and herein known as Physical Activity Coordinators (PACs). The sessions involved a 60-min group activity session, alongside 30 min of themed discussion targeting relevant subjects around physical activity, and 30 min social time. Four of the group activity sessions were planned to be delivered by community physical activity providers identified by the NHS staff delivering the intervention. In addition, one-to-one relational sessions [10] were offered to support motivation, goal setting, and overcoming individual barriers (See Fig. 1 for an intervention infographic).

Our previous work has shown that clinically connected support may be particularly important when people who live with SMI begin changing behaviour, whereas sustained activity is more likely when acceptable community-based opportunities are available over time [7, 8]. The findings from our qualitative study conducted alongside the SPACES fidelity trial identified personalised support, social connectedness, welcoming venues and well-prepared delivery staff as key enablers of engagement [11].

Once people have become more physically active through supervised programmes like SPACES, it is important to support them to find ways to maintain their increased physical activity when the intervention ends. The SPACES co-production process [12] highlighted the importance of having the opportunity to connect with others, connect with local community groups and find ways to sustain physical activity long-term through local community activity providers.

To address the need for socialisation, ongoing social support and maintenance of activity after the end of the intervention, the 18-week SPACES intervention involves up to 4 community taster sessions e.g., walking football or leisure providers. The purpose of the community taster sessions was to support the person in the transition from the SPACES intervention to other forms of physical activity in the community, thereby supporting the maintenance of physical activity and relevant mechanisms for change (e.g., social connection, sense of belonging in the community).

During the SPACES feasibility trial [13], participants and advisors emphasised the crucial role of community-based physical activity providers in helping people with SMI maintain their physical activity after the intervention ends [7, 11]. While SPACES demonstrated that it was feasible for NHS staff to deliver physical activity within a community setting qualitative interviews with the feasibility participants (people with SMI and NHS delivery staff) exposed a critical challenge: sustaining physical activity once NHS structured support ends. Therefore, integrating people with SMI into community-based physical activity is of utmost importance to ensure long-term engagement. Co-SPACES was set up to explore ways to better support PACs to engage with community physical activity providers and to support community physical activity providers in including people with SMI in their provision. The resources generated in Co-SPACES will be used to support PACs and community physical activity providers in the SPACES full scale trial. In addition, it is intended that the resources can be used as standalone resources outside of the SPACES trial to support NHS staff and community physical activity providers to support people with SMI in taking part in community based physical activity.

## Methods

### Aim

The aim of Community-SPACES (Co-SPACES) was to co-produce support resources for community-based physical activity providers and NHS professionals, to enhance physical activity engagement among people living with SMI.

### Definitions

#### Severe Mental Illness (SMI)

There is no one agreed definition of SMI, therefore for the purposes of the SPACES and Co-SPACES studies, and in consultation with the SPACES Patient and Public Involvement and Engagement (PPIE) group, we have defined SMI as being people who would be eligible to be on the UK General Practice Quality Outcomes Framework SMI register.

#### Community-based physical activity providers (community providers)

We refer to community providers as third sector organisations, council funded physical activity providers or small enterprises that provide physical activity.

#### Physical Activity Coordinators (PACs)

Staff employed by the NHS and trained by the central research team to deliver the SPACES intervention.

### Study design

**Fig. 2:**
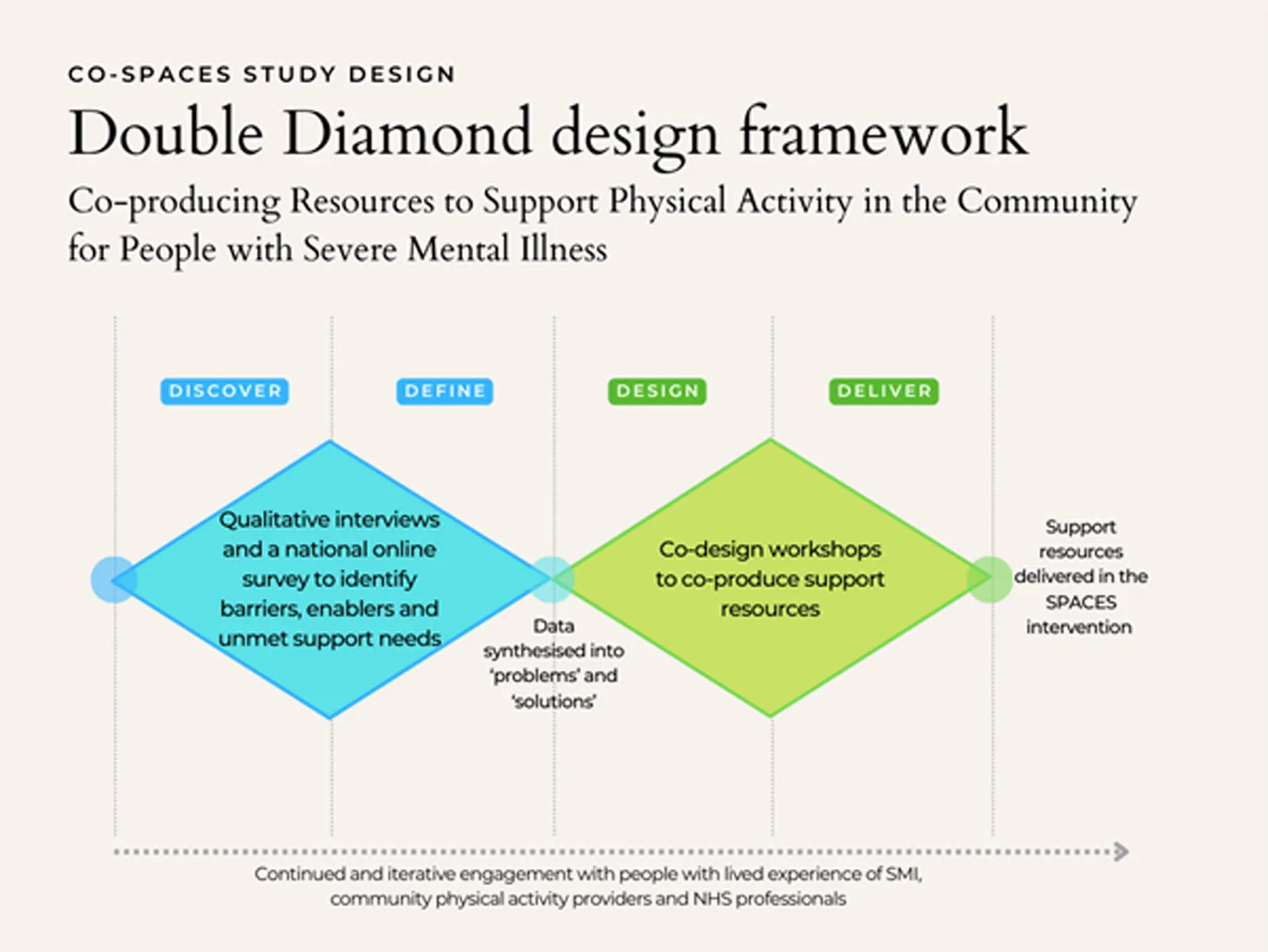
Co-SPACES study design based on the Double Diamond design framework

Co-SPACES was a multi-phase co-production study guided by the human-centred Double Diamond design framework [14] comprising four phases: Discover, Define, Develop, and Deliver (see Fig. 2). The framework was used to structure the co-production process for this study. In the Discover phase, we conducted interviews and an online survey to explore the problem space and understand stakeholders’ experiences, needs, and challenges. In the Define phase, we synthesised these data to clarify and prioritise key barriers and support needs. The Develop phase involved three workshops to generate and refine potential solutions and co-produce support resources. The Deliver phase focused on testing, refining, and finalising the resources within the SPACES trial. Throughout all phases, there was continued and iterative engagement with people with lived experience of SMI, community-based physical activity providers and PACs to ensure end-users experiences were embedded in the co-production of resources.

Patient and Public Involvement was paramount throughout this study and to the development of the resources. To ensure that the views of people with lived experience were included in the process we shared our findings with the PPIE group to obtain their feedback and suggestions (Define phase). People with lived experience of SMI (who were not members of the PPIE group) took part in the stakeholder workshop (Develop phase) and we shared the resources with the PPIE group and asked for suggestions on language and content before finalising (Deliver phase).

Details regarding PPIE is reported fully in Additional File 1 using the GRIPP2-Short Form checklist [15].

### Discover and define—through qualitative interviews and a national online survey

#### Participants

Convenience sampling was conducted to identify community providers to invite to be interviewed and complete the survey. This involved conducting an internet search to identify organisations that provided community physical activity and asking personal contacts involved in the delivery of physical activity and/or mental health care to provide introductions to relevant organisations. Sport for Confidence aided distribution of the survey through their national networks too. Invitations to relevant organisations including sole traders, sporting partnerships, leisure organisations, football association community partners and sporting national governing bodies, were sent by email asking participants to complete an online survey and share with others in their networks.

Recruitment for the interviews took place between May – June 2024. All of the PACS who took part in the feasibility study (*n* = 23) were approached and those who consented to take part were interviewed (*n* = 14). Every PAC trained as part of the SPACES feasibility study was invited to be interviewed regardless of whether they delivered the intervention or not. Community providers participants were purposefully selected to ensure diverse experience.

#### Data collection and analysis

##### Interviews

Conduct of the interviews and the reporting of the findings was guided by the consolidated Criteria for Reporting Qualitative Research (COREQ) [16], which is a 32-item checklist for interviews and focus groups (see Additional File 2).

Interviews sought to explore the methods used to identify and engage community providers, the perceived barriers and enablers to engaging community providers and support needs to facilitate better engagement. Interviews with community providers sought to explore the capacity, willingness and confidence to engage people with SMI in their provision of physical activity opportunities and the resources or support required to do so effectively. The interview topic guides were co-produced with our partner organisation Sport for Confidence (see Additional File 3).

HQ and TB (female researchers who both have a PhD) conducted the interviews online, each was audio-recorded with consent, transcribed verbatim and analysed using framework analysis [17]. Interviews lasted up to one hour. Framework analysis aims to identify, describe, and interpret key patterns within and across cases and themes within the phenomenon of interest [17, 18]. Initial coding, guided by the study objectives, was followed by the inductive development of subthemes. Additional File 4 provides full detail regarding the specific actions taken to achieve each step. NVivo 15 (v1.7.1) data analysis software package and Microsoft’s virtual whiteboard were used to support coding and theme development, conducted independently by GJ and TB. Authors GJ (male researcher with a PhD) and TB met to compare analysis, with any differences being resolved through discussion and consensus.

##### Online survey

The national online survey sought to explore community providers’ perspectives on i) people with SMI attending their physical activity space, ii) their motivation, capability, opportunities and barriers to providing SMI-friendly physical activity facilities and sessions, and iii) their training and support needs to support people with SMI. This paper reports specifically on the training and support needs identified by community providers (point 3).

The bespoke survey was designed through consultation with key stakeholders, including our partner organisation Sport for Confidence and public co-applicant (a person with lived experience of SMI), through reviewing existing literature around inclusion in community spaces (e.g., dementia friendly spaces) and reviewing existing surveys/questionnaires. Where there were no suitable existing surveys, bespoke questions were created by the research team. The survey included both closed and open-ended items and was open from 16th August 2024 until 24th November 2024.

The survey was administered, and data collected, using Qualtrics software. A consent statement was included in the survey. Closed questions were analysed using descriptive statistics and the open questions were coded using Framework analysis.

### Develop and deliver—through co-production of support resources

Three workshops were conducted to co-produce and refine support resources ready for delivery (develop and deliver). Data from the discover and define phase (interview and survey data) were synthesised with input from the SPACES PPIE group. Data were categorised into ‘problems’ and ‘solutions’, which informed the direction of the co-production workshops.

#### Participants

Workshop attendees were purposefully recruited from PACs and community providers identified during the interviews and through additional stakeholders and partner organisations, ensuring representation from people living with SMI, community providers, and professionals. Participants were asked to complete a consent form at the start of Workshop 1 that covered both workshops as described in the participant information sheet. The three interactive workshops involved a total of *N* = 18 participants who together, with the research team, co-produced and refined resources. Notes were taken at the workshops and photos taken of each groups’ Post-it notes placed on posters of the sailboat analogy tool used and of the impact/effort matrix. The workshops were not audio recorded.

#### Data collection and analysis

Workshops 1a and 1b covered identical content and were held with participants in Sheffield (*n* = 6) and London (*n* = 12) in a community space. Workshop 1 (see Additional File 5) aimed to clarify and refine the themes identified in the interviews and survey and to generate ideas for the support resources. Workshops were facilitated by the Co-SPACES research team (TB, GJ, MF, KM, HQ and EP). KM, HQ, EP and TB are female and have a PhD, GJ is male and has a PhD, and MF is male and has an MSc). Following a brief presentation of the findings from the interviews and survey, workshop attendees took part in structured, interactive activities adapted from the Lightning Decision Jam technique [19] to support prioritisation and decision-making. Data from workshop 1 was analysed into themes which were presented at workshop 2.

Workshop 2 (see Additional File 6) included a subset of participants from workshop 1a and 1b and included representation from all stakeholder groups (i.e., lived SMI experience, community provider, NHS staff trained to deliver the SPACES intervention). We invited everyone who attended workshops 1a and 1b to attend workshop 2 (*n* = 8). Participants participated in interactive activities focused on developing and defining solutions that were practical and achievable within the constraints of the Co-SPACES study. We asked participants to categorise proposed solutions using a traffic light framework based on their anticipated impact and feasibility. Anticipated impact and feasibility were discussed and consensus agreed among the workshop attendees based on resources, time and effort needed. Solutions were classified as green (high impact and easy to achieve within Co-SPACES), amber (high impact and achievable within Co-SPACES), or red (high impact but outside the scope of Co-SPACES, and low impact and outside the scope of Co-SPACES). Workshop attendees were offered a £75 voucher as a gesture of thanks for their participation. Travel and lunch were also provided.

## Results

### Interview findings

The interview study sought to identify enablers, barriers, and necessary support for involving community providers in the provision of activity for people with SMI. A total of 32 in-depth, semi-structured interviews were conducted (15 PACs and 17 community providers) between May and November 2024, see Table 1 for participant characteristics. The themes identified follow below:

**Table 1:** Participant demographics

PAC ID	Sex	Ethnicity	Role within SPACES
P01	M	White British	Feasibility study PAC
P02	F	White British	Feasibility study PAC
P03	F	White British	Feasibility study PAC
P04	M	White British	NHS Research officer
P05	M	White British	Feasibility study PAC
P06	F	White British	Feasibility study PAC
P07	F	White British	Feasibility study PAC
P08	F	White British	Feasibility study PAC
P09	F	White British	Feasibility study PAC
P10	F	White British	NHS research officer
P11	F	White Other	Feasibility study PAC
P12	F	White British	Feasibility study PAC
P13	M	Black British	Trained but didn’t deliver
P14	F	White British	Newly trained for full RCT
P15	M	White British	Newly trained for full RCT

Community Provider ID	Sex	Ethnicity	Organisation type
CP01	F	White British	Activity classes
CP02	F	White British	Activity classes
CP03	3	White British	Community health service
CP04	F	White British	Development Trust
CP05	M	White British	Performance Training
CP06	M	White British	Outdoor activities charity
CP07	M	White British	Outdoor activities charity
CP08	M	White British	Community wellness service
CP09	F	White British	Community wellness service
CP10	F	White British	Outdoor activities charity
CP11	M	White British	Community activity hub
CP12	F	White British	Outdoor activities charity
CP13	M	White British	Leisure facility
CP14	M	White British	City health and wellbeing programme
CP15	M	White British	Leisure facility
CP16	F	White British	Community group
CP17	M	White British	Outdoor activities charity

#### Theme 1: utilising existing relationships and networks to find community providers

Successful involvement of community providers in the provision of activity for people with SMI was enabled by the strength and quality of existing relationships. Established links with local community providers is said to enable a smoother collaboration, whereas a lack of existing connections makes it difficult to identify suitable community providers. As one PAC explained, “*Community providers do exist — I just couldn’t find them*” (P14). Wherever engagement with community providers was less successful, the PACs described limited time and capacity to identify providers, lack of knowledge of local provision, and uncertainty about providers’ ability or confidence to accommodate participants with SMI. One PAC described the absence of community connections as “*the Achilles’ heel of SPACES project* (P02), noting that participants’ long-term motivation depended on “*trusted community networks*” being in place.

Suggested solutions to establishing these relationships and networks included mapping local resources, identifying key community and local leaders, and introducing a dedicated link role to “*join the dots*” (P02) between health services and local community providers.

#### Theme 2: finding ‘appropriate’ community providers with desirable characteristics

Successful involvement of community providers in the provision of activity for people with SMI required not only finding community providers but finding ‘appropriate’ providers with desirable characteristics.

Appropriate providers were said by the PACs to be those that demonstrated an awareness of SMI and confidence to work alongside people with SMI. PACs were aware that the appropriateness would change depending on the needs of the individual. A recurring concern among PACs was that the community providers they engaged with had limited awareness and confidence, to support individuals with SMI. PACs reported encountering occasional stigma, uncertainty, and apprehension among providers. One PAC highlighted the need for “*basic education and advice — a protocol — on making sessions more welcoming and less anxiety-building*” (P01). When the providers demonstrated confidence and willingness to engage people with SMI and interpersonal qualities such as empathy, flexibility and the ability to create a safe and welcoming place, the PACs felt reassured that this was an appropriate provider.

Suggested practical strategies to address this lack of awareness included mental health awareness training for all staff (including front-of-house personnel), clear communication about participant needs, and simple environmental adjustments, such as optimising room layout and providing quiet areas.

As well as knowledge, community providers’ soft interpersonal skills, such as empathy, patience, and flexibility, were valued more highly than formal physical activity qualifications. Community providers who created welcoming and safe activity environments and were willing to adapt activities to meet the needs of participants were especially commended. One PAC observed that effective providers “*demonstrated a genuine interest*” in participants and “*understood the impact of medication on fluctuating motivation”* (P05).

Conversely, it was reported that some community providers lacked the characteristics needed to meaningfully engage with people with SMI. The unfavourable characteristics reported included a lack of consistent follow-up on the part of the community provider to PAC queries, reliance on inaccessible digital booking systems, or being located too far from participants’ homes. In such cases, PACs emphasised the need to support and upskill community providers to improve the accessibility and continuity of their provision.

#### Theme 3: the need for a person-centred transition into community spaces

Successful involvement of community providers in the provision of activity for people with SMI was said to need a gradual, person-centred transition into community-based physical activity settings. Building confidence and trust between community provider and the service user entering this community provision was seen as a process requiring time and continuity: “*It [feeling confident about joining a new group] takes more time than you think*” (P01). Both PACs and community providers stressed the importance of identifying and addressing each participant’s unique motivations to maintain their engagement in physical activity. As one community provider described: *“Honestly I’ve never seen owt like it in my life… So, when you get the right motivation of an individual, that’s what I’m saying and you get the right environment, magic can happen”* (CP11).

To facilitate this process, one community provider proposed the use of “*Activity Passports*” (CP03) that people with SMI could take to community providers to share their goals, interests, and preferences more confidently at the outset.

#### Theme 4: supporting community providers with training

Community providers expressed enthusiasm for additional training around SMI, not only for instructors but for all staff likely to have contact with people with SMI. Preferred learning formats included on-the-job observation, workshops, and blended or peer-led approaches. “*Observing and learning from centres excelling in supporting people with SMI is invaluable*” (CP04) noted one provider. Community providers also requested more practical, less theory-heavy mental health training and explicit guidance on using physical activity spaces creatively to accommodate participants’ diverse needs.

### Online survey findings

The national online survey sought to explore community providers’ perspectives on involving people with SMI in their provision. This paper reports specifically on the training and support needs identified by community providers.

In total, 52 respondents completed the survey anonymously from across the UK and Ireland. As we used a snowballing technique to recruit participants, we cannot determine how many providers were invited to take part in the survey. Although this approach limits the assessment of representativeness, it did enable access to a broad range of providers. Respondents represented small, medium and large organisations as well as sole traders, fitness or leisure operators, active partnerships, sports clubs, and community interest companies. Respondents described their organisations as gyms, leisure centres, fitness centres, sports clubs, community sports venues, swimming pools, outdoor sports complex, exercise classes, personal trainer services, boutique gym/franchises, green- or blue-space activity providers and sports venues. Just under half (41.5%) of the organisations had a designated Mental Health First Aider and 62.3% reported having an initiative that targeted mental health.

#### Community providers’ support needs

Analysis of survey responses identified a vital need to know more about SMI, with one respondent admitting “*I have no idea what I need to put in place”* to engage with people who have SMI*.* We will now describe the key themes related to training and development needs among the community providers.

#### Theme 1: increasing knowledge and understanding of SMI

Community providers expressed a need to know more about mental health conditions and SMI. There was a need to not only define SMI and its symptoms, but to “*understand what it is like*”, “*what to expect*”, the behaviour traits that might be demonstrated and “*common triggers*” that might be experienced. One respondent also identified the need to understand how medication may affect the ability for people to participate in physical activity. Respondents reported that this knowledge and awareness needed to transcend all levels of the organisation, from front line staff (front-of-house personnel), instructors, activity deliverers and management. One respondent believed that physical activity training/qualifications should incorporate mental health. Another believed that the best place to start was to consult SMI groups to “*find out what activities they would like to try and what would help them to join in and specifically if they want to join our main classes or if they want their own*”.

#### Theme 2: adapting the setting

Community providers recognised that some adaptations to their physical activity setting would be needed to engage with people who have SMI. There was a need to know how to plan, adapt and develop new sessions that are appropriate for people with SMI (taking into consideration noise levels, and setting busyness during peak/off-period times). One provider said they would need designated safe spaces in their setting. Another felt they would need to change their instructor-to-participant ratios to accommodate those with additional needs, with another saying they would need budget for additional staff. Other adaptations suggested included “*specialist equipment*” and “*mental health screening and risk assessment for new members.*”

#### Theme 3: developing interpersonal skills

Community providers expressed a need to know how to communicate and behave appropriately when they engage with people who have SMI. They mentioned communication skills, including listening, trauma-informed communication and non-verbal communication such as body language.

#### Theme 4: having crisis management procedures

Community providers needed reassurance that they would know how to support someone who became unwell or experienced crisis in their setting. There was a sense of concern and lack of confidence in “*coping with challenging behaviour*” and “*how to handle situations where the person becomes distressed, or upset, or has a mental health crisis when in the centre*”. Related to this, one provider expressed a need for suicide awareness training, and another requested additional safeguarding training.

#### Theme 5: accessing specialist support

The value of working alongside specialist mental health support was reported. With some community providers believing that mental health professionals need to be involved and work alongside activity professionals. Community providers wanted to know what organisations to signpost and refer people with SMI to.

Overall, the survey findings reveal that community providers have a strong need to know more about SMI before being confident and able to engage with people who have SMI. The survey findings complement those from the interview study, highlighting an enthusiasm to be inclusive but adding a new perspective of worry and concern about how best to provide effective and welcoming support for people with SMI.

### Workshop findings/output

#### Co-production of support resources

Together, the interview and survey findings informed the content of the co-production workshops and the development of resources to strengthen the involvement of community providers in the provision of activity for people with SMI. The first workshop aimed to clarify and refine the themes identified in the interviews and survey and to generate ideas for the support resources. Using the traffic light framework, three categories of actionable ideas for the support resources emerged from the workshops. Green actionable ideas could be implemented immediately, primarily relating to the enhancement of the SPACES intervention. The changes involved updating the PAC training and included guidance around building networks, strengthening links with local and national activity providers, addressing participant travel fears, and providing additional social support to participants attending community-based sessions.

The amber actionable ideas directly informed the co-production of support resources. The resources needed to include practical guidance on adapting spaces (venues, facilities) and sessions to overcome barriers and create SMI-supportive environments, with illustrative case studies showing effective venue adaptations. Lived experience narratives would make training materials more meaningful and myth-busting information about SMI and checklists for community providers, would provide clear guidance for community providers.

Red actionable ideas were considered high impact but outside the immediate scope of Co-SPACES, often because they involved structural or service-level constraints (e.g., utilising link workers, funding, transportation), or low impact and outside the immediate scope of Co-SPACES. These were documented for future development.

By the end of workshop 2, attendees had finalised important content of the resources to be produced and a prototype booklet (an amber resource). A complimentary amber resource was requested, that of a short video to complement the booklet.

##### The booklet

The booklet aimed to provide an overview of SMI (what it is, why physical activity is important and what it’s like to enter a community physical activity space) from lived experience perspectives using quotes from service users, guidance for community providers on creating inclusive and welcoming environments, advice for future PACs and NHS professionals on effective engagement with community providers, case studies and examples of good practice. The appropriateness of the providers was summed up as the three P’s; people, place and provision, identified as key to enabling people with SMI to feel comfortable and all these needed to be present. If one P was missing, the provider was not felt to be appropriate by the PAC and potentially a person with lived experience, may feel uncomfortable and not attend. The prototypes from the workshop were transformed by a professional graphic designer into an A5 eight-page full colour booklet (see Additional File 7). Workshop participants and the SPACES PPIE group were invited to review and give feedback on the first iterations of the booklet before being revised and finalised. The resources are being incorporated into the SPACES trial intervention and will be evaluated in that context.

##### The video

The video entitled ‘*Creating Welcoming and Safe Activity Spaces for People with Severe Mental Illness’* and available via a QR code in the booklet, demonstrated similar themes to the booklet, but in another format, with the intended purpose to aid community provider recruitment for taster sessions for the SPACES intervention. The video features people with SMI sharing their experiences of taking part in community physical activity. The video provides a narrative of the benefits of physical activity provided within in a community setting for those with SMI, from the perspective of SMI lived experience, NHS staff and the community provider.

## Discussion

Co-SPACES has co-produced two resources to support NHS trust engagement with community providers, and to, in turn, aid community providers knowledge and confidence to engage people with SMI in their provision. This will support people with SMI to participate in community-based physical activity thus enabling long-term maintenance of activity initiated through SPACES. The Double Diamond model guided the process. The produced Co-SPACES booklet and video serve two main purposes: 1) to augment the SPACES intervention by facilitating the involvement of community providers and supporting maintenance of physical activity after the SPACES intervention ends, and 2) to raise awareness of SMI among community providers and optimise the likelihood of community activity spaces being welcoming and inclusive of people with SMI.

The findings from across the interviews, survey and workshops highlight barriers and enablers to community physical activity engagement among people with SMI. They highlight the importance of community provider characteristics and the essential role of the person-centred transition to community activity spaces. The Co-SPACES resources reiterate that supporting people with SMI to engage in community physical activity requires more than simply signposting to local services, rather additional, personalised support is needed [20]. Existing literature shows the gap between clinical services and community provision for people with SMI, putting them at risk of exclusion if their unique needs cannot be met [21]. In the physical activity context, research has demonstrated that for people with SMI, the transition to community-based activity is a long, complex, and often challenging period that requires careful, personalised support [8]. People with SMI face a myriad of challenges to community-based physical activity including isolation, stigma and discrimination [22]. Some of the barriers are said to directly relate to the lack of education about mental illness in the physical activity and fitness industry [23, 24]. However, little is known about community providers’ own perspectives on supporting people with SMI, including their perceived barriers and training needs.

Co-SPACES adds to the limited evidence-base regarding community physical activity provision for individuals with SMI. There is a lack of direct evidence exploring the viewpoint of exercise/fitness professionals. By focussing on the perspectives of those providing the activity, we have found that whilst there might be enthusiasm and willingness to welcome and support people with SMI into community activity spaces, often activity community providers lack the knowledge and confidence to provide appropriate support. This mirrors findings that show unmet training needs among exercise professionals, where evidence shows the need for mental health training for exercise instructors to raise awareness, improve knowledge and reduce stigma and discrimination [25]. The Co-SPACES resources fulfil part of this gap, specifically what it may feel like for a person with SMI to experience accessing community provider opportunities, and even what provision could help accessing such opportunities. However, there is still much needed to be done here to properly address known barriers previously highlighted in the literature, and in the present findings namely practical-based community provider mental health training. In a 2018 consensus statement, Rosenbaum stated that to effectively integrate physical activity into mental health condition management, exercise professionals must receive training on mental health literacy and illness-specific considerations [26].

Co-SPACES and the co-produced support resources play a key role in beginning to bridge the SMI knowledge gap among community providers. National guidance, community provider training packages and stronger NHS-community links are essential if we are to truly bridge the gap between clinical mental health services and community-based physical activity provision.

### Implications

#### Implications for community providers

The Co-SPACES resources directly address some of the identified needs of community providers. They have the potential for improving the inclusivity of community activity spaces for people with SMI. By raising awareness among community providers and offering strategies (through the booklet’s guidance and video’s personal testimonies), these resources target known barriers to engagement such as stigma, lack of knowledge, and fear of crisis situations. In doing so, they provide a mechanism to improve the transition of people with SMI into community physical activity settings, which was the key challenge this study aimed to address. The Co-SPACES resources could act as a springboard for further intervention development to fully address highlighted needs beyond the scope of what was possible for Co-SPACES, which would further strengthen and standardize practice for community providers to meaningfully support people with SMI access community physical activity opportunities.

#### Implications for health systems

The resources provide guidance on how health systems can effectively engage with community providers and highlight the importance of supporting person-centred transitions to community spaces. The findings also suggest that there is a pressing need for investment in community provider training. Co-SPACES has identified training opportunities for community providers so that pathways between clinical and community services are appropriate, supportive and sustainable. The support resources aim to increase knowledge, address stigma, provide reassurance and encourage community providers to create safe and welcoming spaces for people with SMI.

Ultimately, implementing these support resources more widely could help close the gap between clinical care and community physical activity provision for people with SMI.

### Strengths and limitations

The major strength of Co-SPACES is its co- production methodology, embedding the perspective of multiple stakeholders into the support resources produced (lived experience service users, community providers, NHS professionals). The study’s use of multiple data sources across stakeholder groups strengthens the credibility of the findings. In addition, the commitment to lived experience input adds authenticity and impact to the resources produced. However, the samples involved in Co-SPACES may be skewed towards those with an existing interest in inclusion and mental health, meaning that the ‘concerns’ identified may not be truly representative of community providers more generally. Other considerations relate to the time and cost constraints. The Co-SPACES timeline and budget determined the scope of what could be developed and implemented. Whilst the workshop participants generated a rich array of ideas and recommendations, pragmatic decisions were required about which elements could be prioritised within available resources. This has resulted in some valuable co-produced solutions being deferred or excluded from the final outputs e.g., a practical-based mental health training course for community-based physical activity providers).

We have not yet assessed the impact of these resources on provider behaviour or participant outcomes. A formal evaluation in diverse communities is required. Future work should explore how best to disseminate and embed these resources within existing pathways, assess their impact on community provider confidence and participant outcomes, and consider their adaptation for other underserved populations facing similar barriers to community engagement. Engaging national bodies that oversee training for fitness professionals could enhance the broader and sustained use of our resources. This project underscores the value of co-production with end users in developing practical solutions to real-world challenges. By actively involving lived experience service users, community providers and NHS professionals we created resources that are grounded in real-world needs.

## Conclusions

In summary, co-producing solutions with service users, community providers, and professionals resulted in practical resources that address the gap between clinical support and community-based physical activity. These resources have the potential to improve the inclusivity of community sport and exercise environments for people with SMI, which may support longer-term lifestyle change beyond short-term interventions. If implemented at scale, these resources have the potential to reduce health inequalities faced by people with SMI by supporting sustained engagement in community physical activity and offering practical strategies for creating welcoming and accessible activity spaces.

## Supplementary Information

Additional file 1.

Additional file 2.

Additional file 3.

Additional file 4.

Additional file 5.

Additional file 6.

Additional file 7.

Additional file 8.

## Data Availability

The booklet and link to the video can be found in Additional File 7. The anonymised data files are available upon request form the corresponding author.
