# Supplementary material for: Bridging the gap: co-producing resources for NHS mental health trusts and community providers to support people with severe mental illness live active lives in their community
Source: BMC Public Health. 2026 Jun 16;26:2418. doi: 10.1186/s12889-026-27936-7 (PMC13501637; doi:10.1186/s12889-026-27936-7)
Supplement: Supplementary file 2 [file 12889_2026_27936_MOESM5_ESM.pdf]

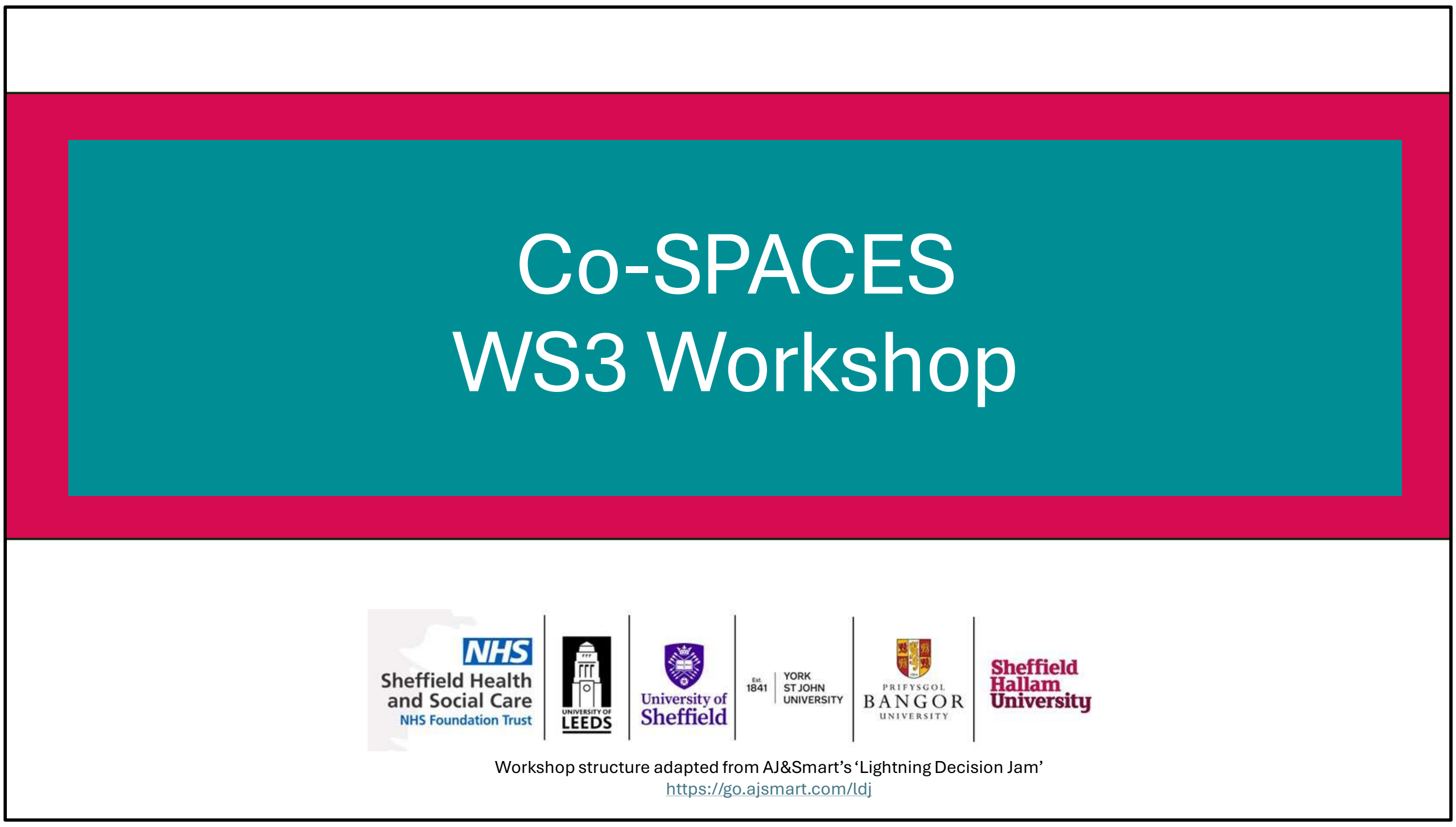

- Welcome everyone
- Housekeeping – Fire/toilets/rules of the workshop
- Introductions

Lead facilitator pack

- PowerPoint slides
- Presentation notes
- Agenda/feedback form
- Printout of themes from WS1 and WS2
- Example table pack items
    - Blue, pink, purple, yellow sticky notes
    - Sticky dots
    - Scissors
    - Black sharpie pen
    - Biro
    - Phone/timer to use throughout each task

Table facilitator packs (1 per table of 6 participants and 1-2 facilitators)

- Presentation notes (in colour)

- Name tags
- Sailboat poster
- Impact/effort scale poster
- Print out of themes from WS1 and WS2
- Square sticky notes (blue, pink and purple)
- Rectangular sticky notes (yellow)
- Sticky dots
- scissors
- Black sharpie pen per person
- Biros one per person
- Agenda and feedback sheet one per person
- **Mobile phone/camera to take photos at every stage to record your table's thoughts for data analysis**
- Envelopes to keep sticky notes in

Co-SPACES
WS3 Workshop

A pre-warning / apology

Workshop structure adapted from AJ&Smart's 'Lightning Decision Jam'
https://go.ajsmart.com/ldj

- Pre-warn everyone that the pace of the tasks undertaken, will by the end seem too fast.
- People will feel they wanted to talk more and discuss ideas with each other in more depth
- Explain the reasons why this fast-paced approach has been taken:
    1. Potentially lots to ideas to uncover
    2. Want to extract as much information from people in the most efficient way possible
    3. Don't want to get sucked into one or two issues only
    4. Want all ideas to be heard and for everyone to 'hear them' and consider them
    5. Want to vote democratically on what should be prioritized.
    6. Want to keep everyone active and engaged

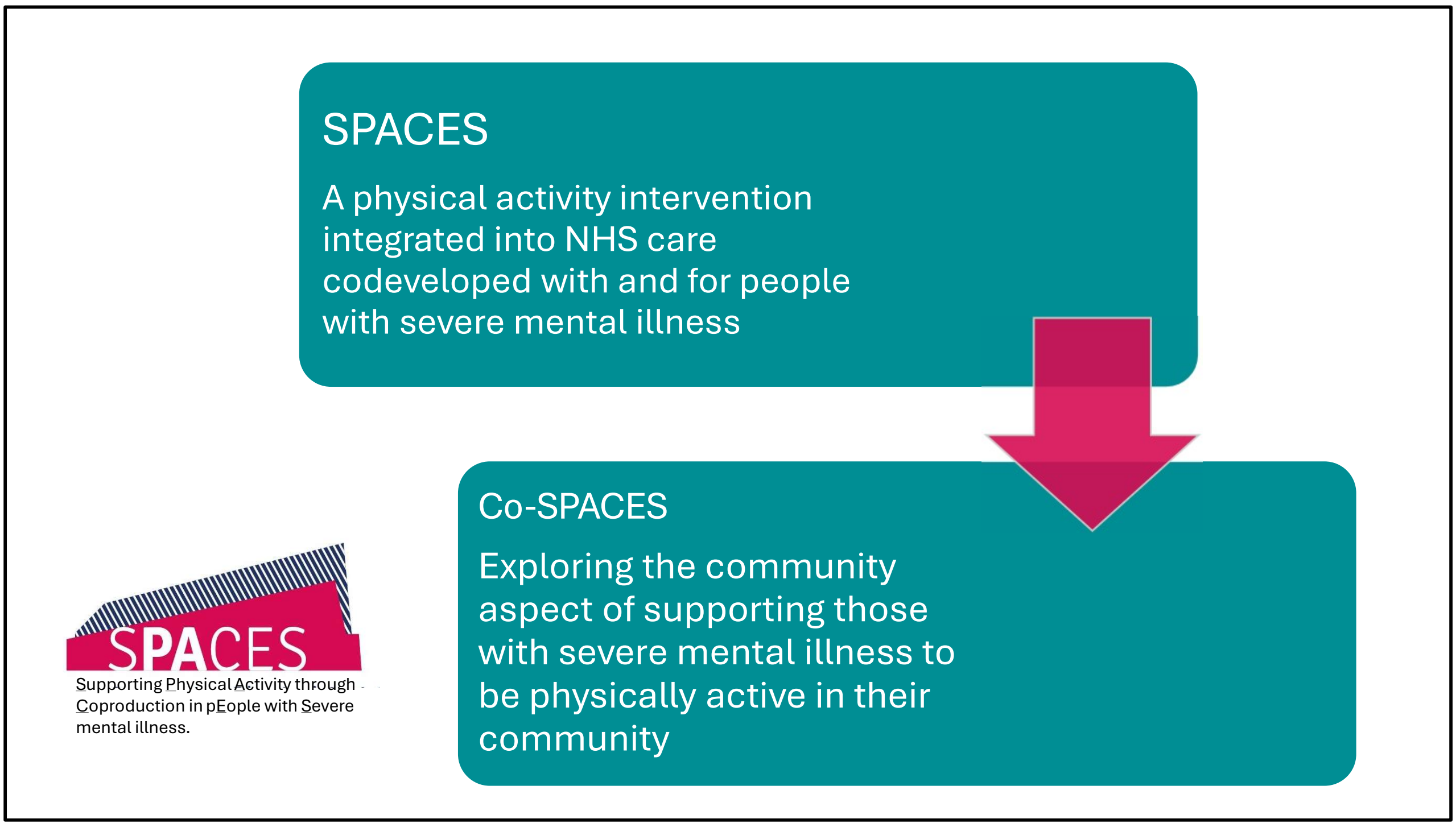

- Reminder /explanation of the SPACES intervention, and role of community provider.
- Explanation of the issues experienced during the feasibility study identifying and engaging community providers and the thus the need for this additional Co-SPACES work.
- WS1 interviews to determine what works and what doesn't
- WS2 online survey to gauge people's understanding of SMI and the PA provision available
- WS3 Workshops to prioritise the problems and consider most impactful solutions, requiring effort available within the Co-SPACES time and budget

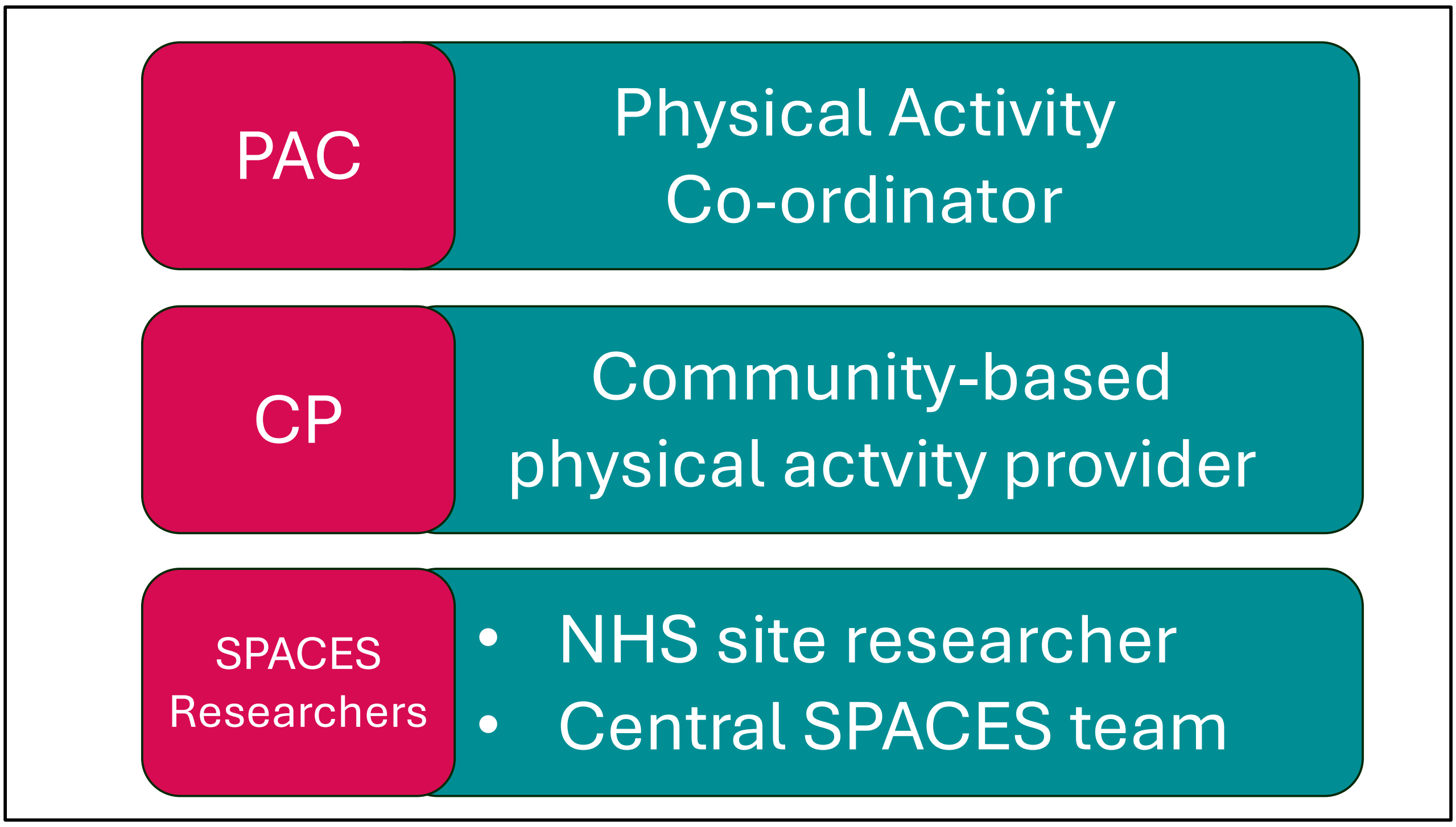
PAC
Physical Activity Co-ordinator
CP
Community-based physical actvity provider
SPACES Researchers
• NHS site researcher
• Central SPACES team

What is SMI

PPIE Definition:

"Severe mental ill health (SMI) means different things to different people. But in the UK, it's officially defined as being diagnosed with conditions like schizophrenia and bipolar disorder.

While it's different for everyone, these conditions can lead to people living with psychosis and experiencing hallucinations or delusional thoughts .

Being diagnosed with a SMI usually means that people experience things differently and this can make life more difficult."

What SMI is not

This definition should not be seen to imply that other diagnoses (e.g., PTSD, anxiety disorders, depression, and neurodiverse diagnoses) are not ‘serious’ or ‘severe’, or that they do not carry any associated physical health risk.

This definition is used to align this study with NICE guidance for physical health checks and the scope of the Quality and Outcome Framework (QOF) SMI register.

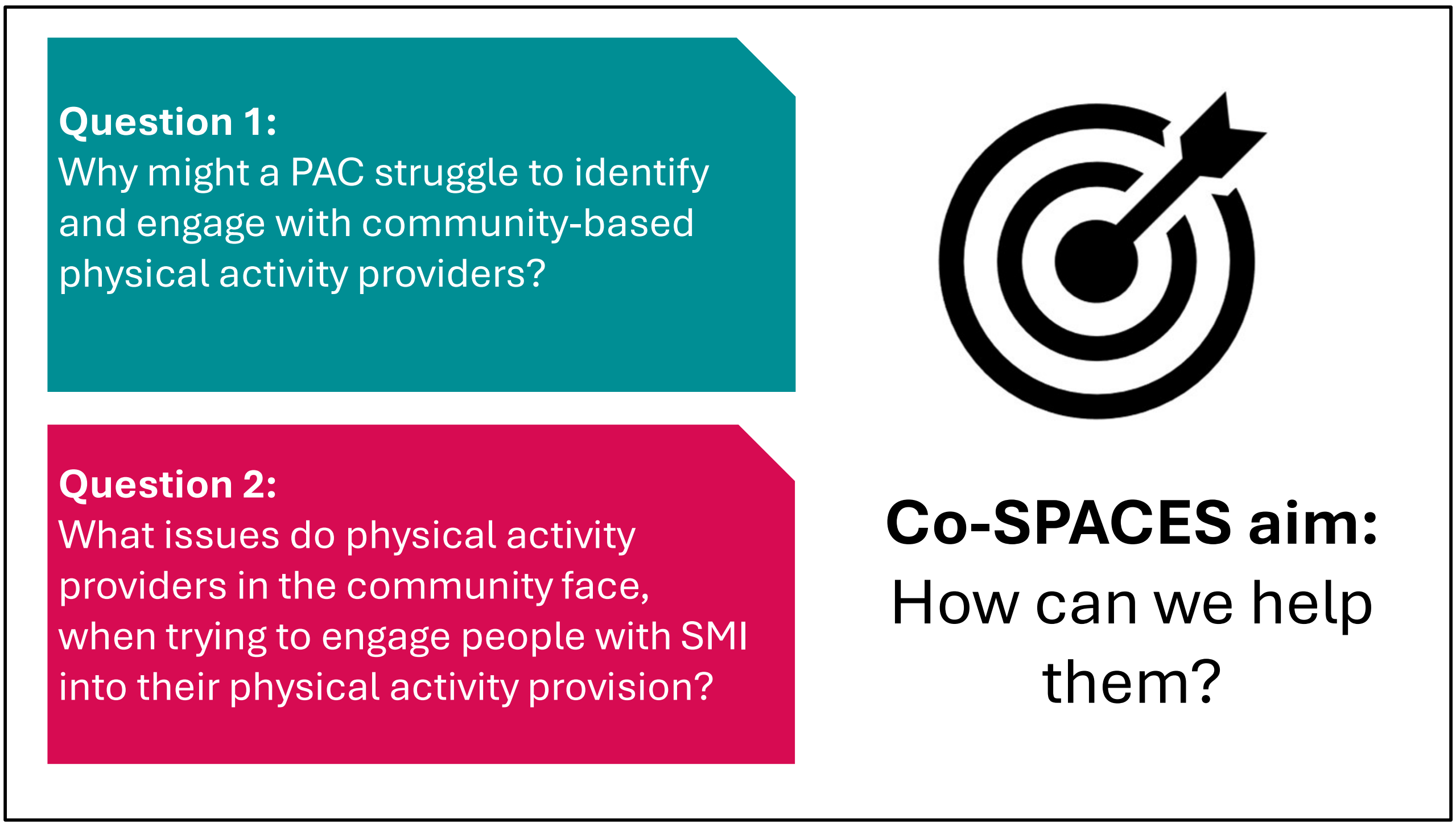

During the feasibility study two problems emerged:

- NHS based physical activity co-ordinators (PACs) struggle to identify and engage with community-based physical activity providers?
- Physical activity providers in the community struggle to engage peoplewith severe mental illness in theirphysical activity provision?

Two research questions:
Q1: Why might a PAC struggle to identify and engage with community-based physical activity providers?
Q2: What issues do physical activity providers in the community face when trying to engage people with severe mental illness in physical activity provision?

In our initial bid for Co-SPACES funding, we considered a practical based training course for PACs and support materials for community-based providers would be of use.

- This may still be the case, but not necessarily.
- Your input through this workshop will help shape what support is best/pragmatically possible.

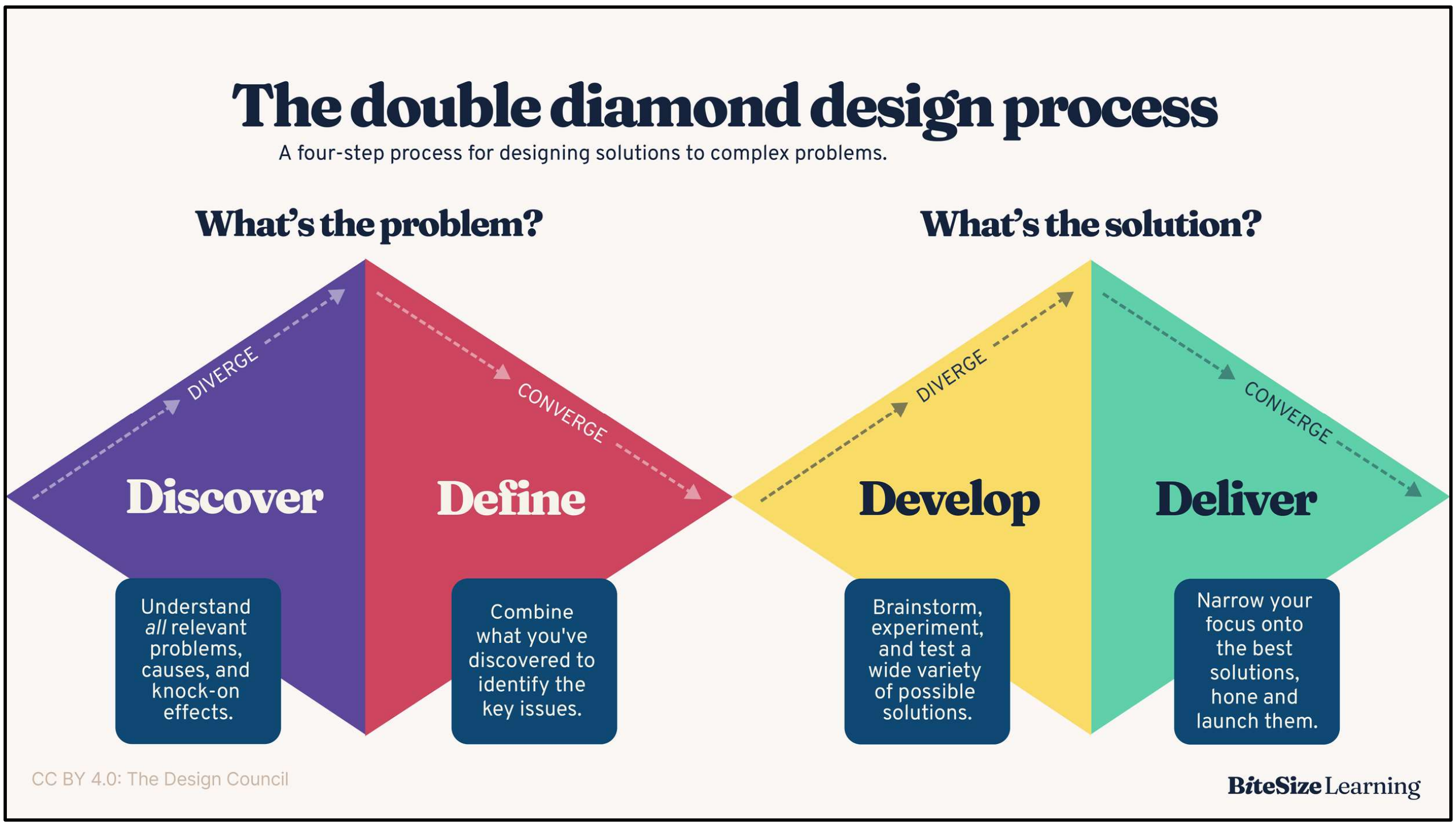

To help us answer these questions we are using the double diamond design process.

DISCOVER

- This process began with WS1 and WS2, discovering and beginning to understand all the relevant problems, causes, and knock on effects that surround PAC and SMI engagement with community providers
- Some of you have completed a questionnaire and/or interview already – thank you

DEFINE

- We have combined what we have discovered so far, but need to you to identify and priortise the key problems

DEVELOP

- After lunch we will brainstorm and consider a wide variety of possible solutions

DELIVER

- We will also narrow our focus onto the most pragmatic solutions to action
- The research team will then go away action your ideas and meet with you again in July to confirm that what we have created meets your expectations
- Once you are happy we will be able to launch these solutions.

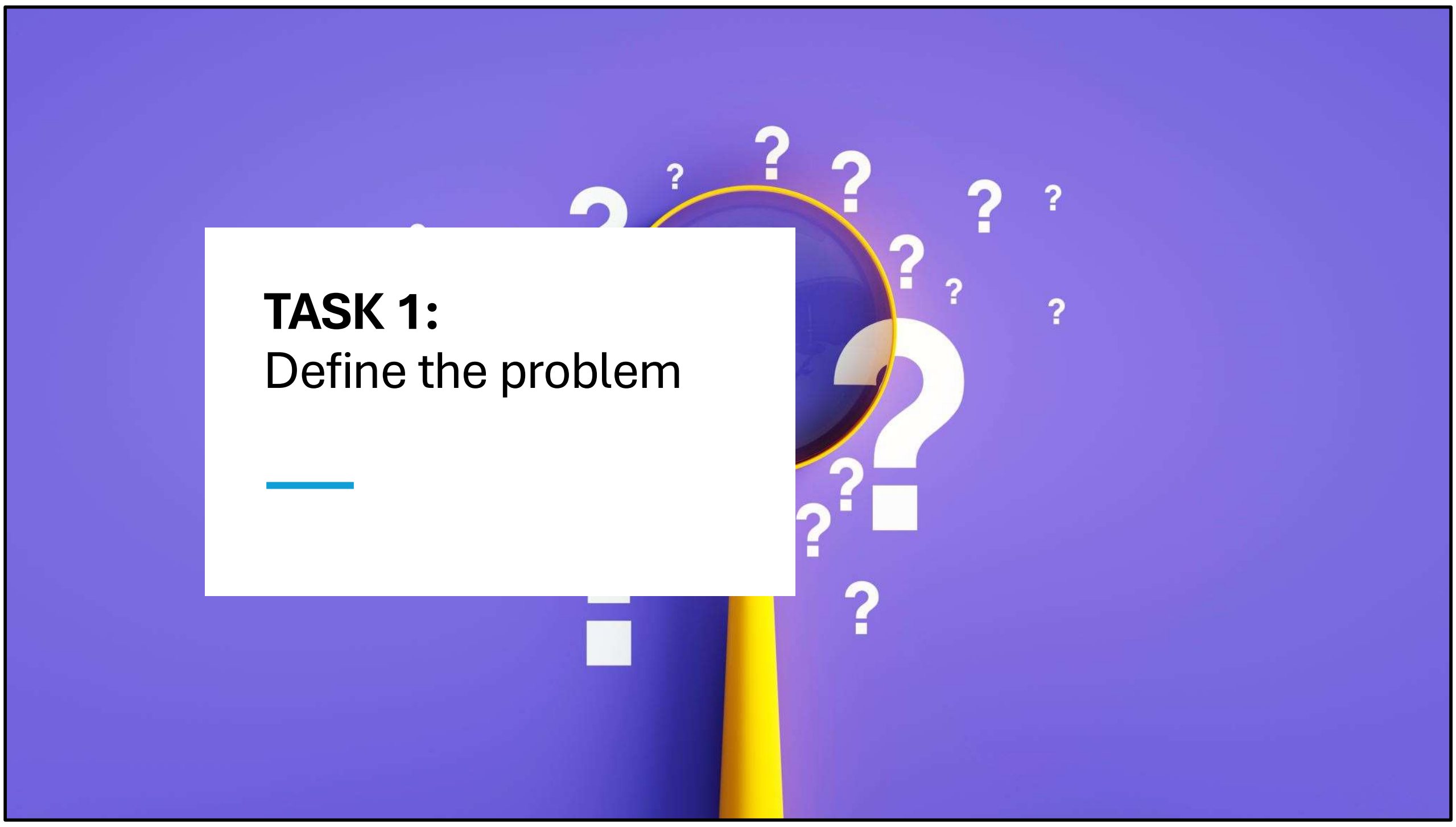
TASK 1:
Define the problem

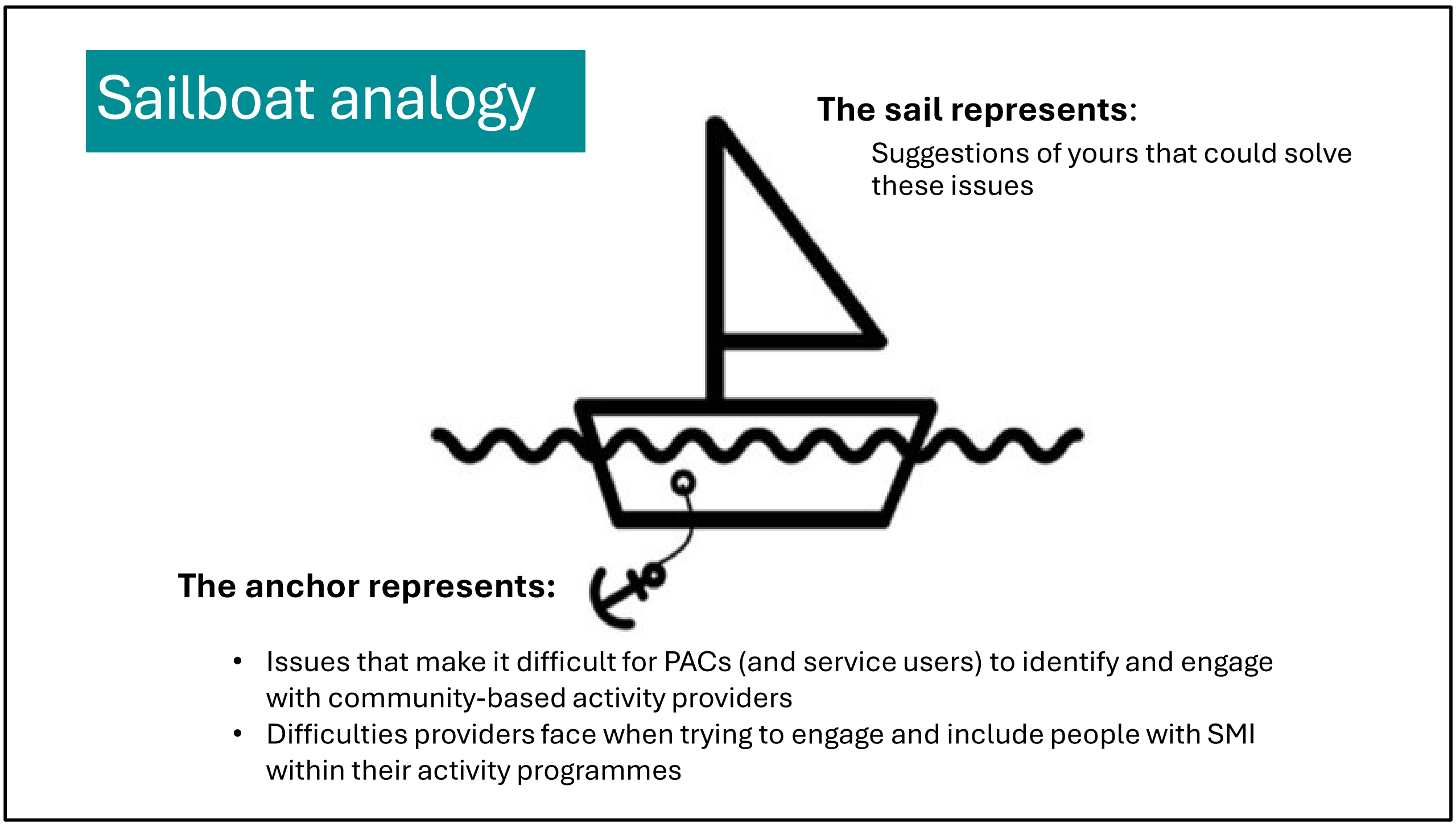

We will be using a sailboat analogy to help structure today's workshop

Anchor

1. Represents issues holding PACs back, making it difficult to identify and engage with community-based providers
2. Represents issues community-based providers have in engaging with people with SMI in their PA provision

Sails moving the sailboat forward

1. Represents current and suggested good practice that facilites easy identification and engagement with community-based providers
2. Represents current and suggested good practice that facilitates increased engagement with people with SMI amongst community-based providers

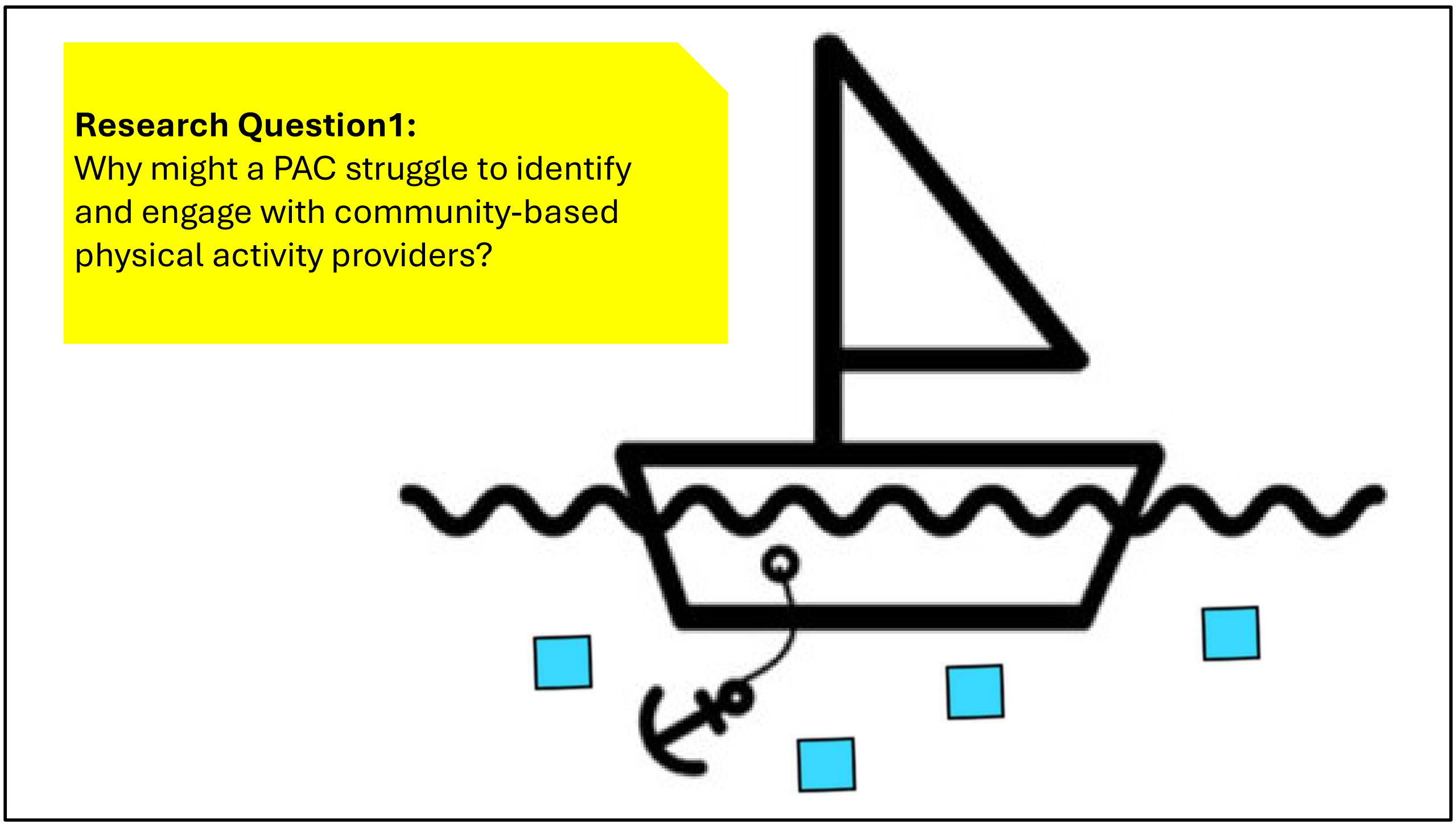

- Split into two groups and use two sailboat boards per table for this exercise
- Explain themes generated from WS1 and WS2 – handout A4 double sided sheet
- Facilitator to describe these and add a sticky note (one for each theme/problem) to the main board. Table facilitators to add them to the table boards on.
- Group to write on a sticky notes any other issues they feel add to the problem – one sticky note per problem,
- **No discussion at this stage, everyone works together alone (5 mins)**
- Group to quickly and quietly place their sticky notes on to their table's board – still no discussion (1 minute)

Prioritise the problems

- Table facilitators please take the problems voted on and arrange them in order of priority.

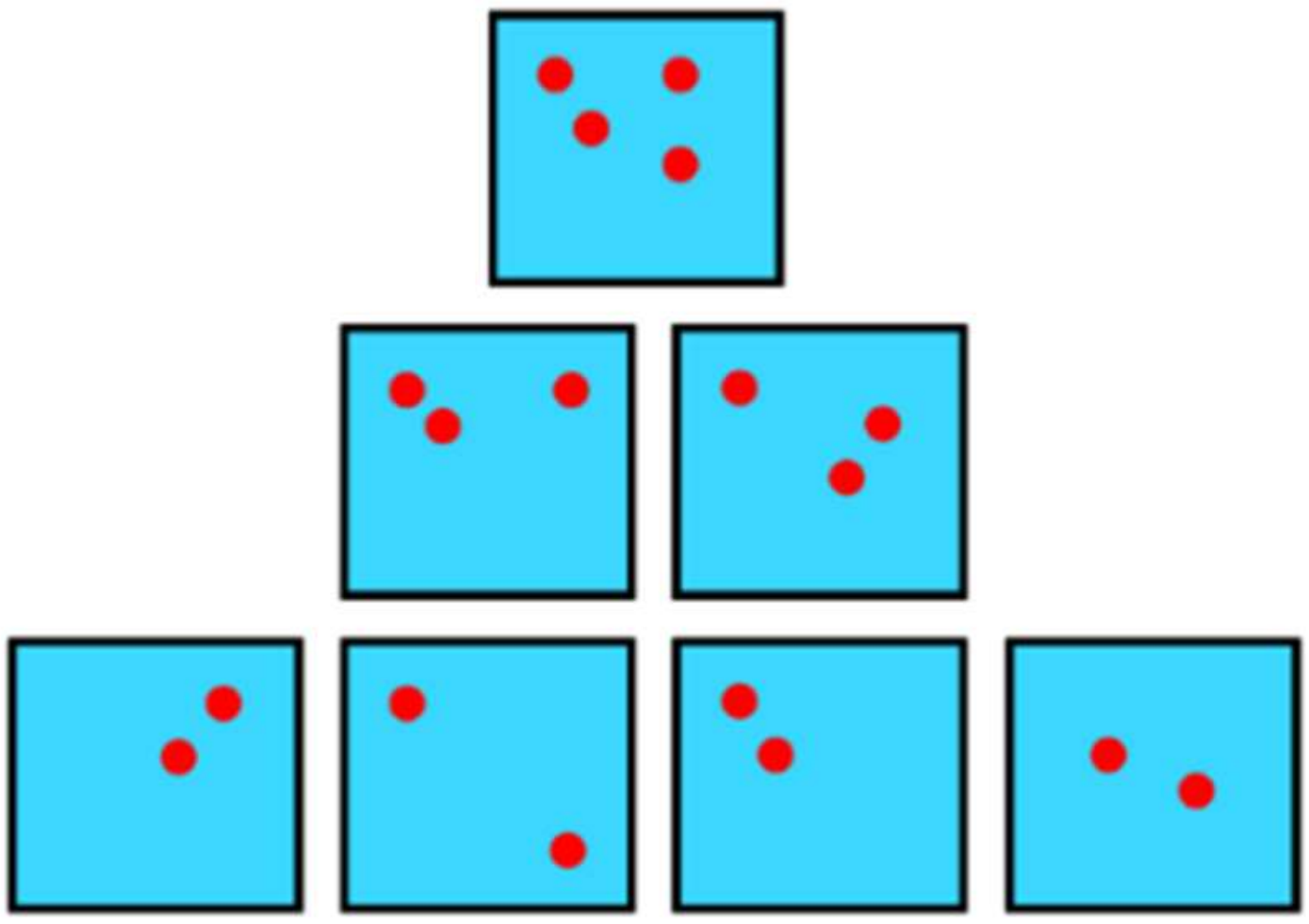

- Table facilitators reorganize the sticky notes in order of priority
- We will come back to these after lunch

Prioritise the problems

- 3 dots per person
- Vote on the problems you consider to be the biggest issues.
- You can vote on their own sticky note
- You can put more than one dot on a problem of you feel strongly about it.
- DUPLICATES – if you sees duplicates place them on top of each other; make sure all the dots are on the top-most sticky note
- 3 minutes

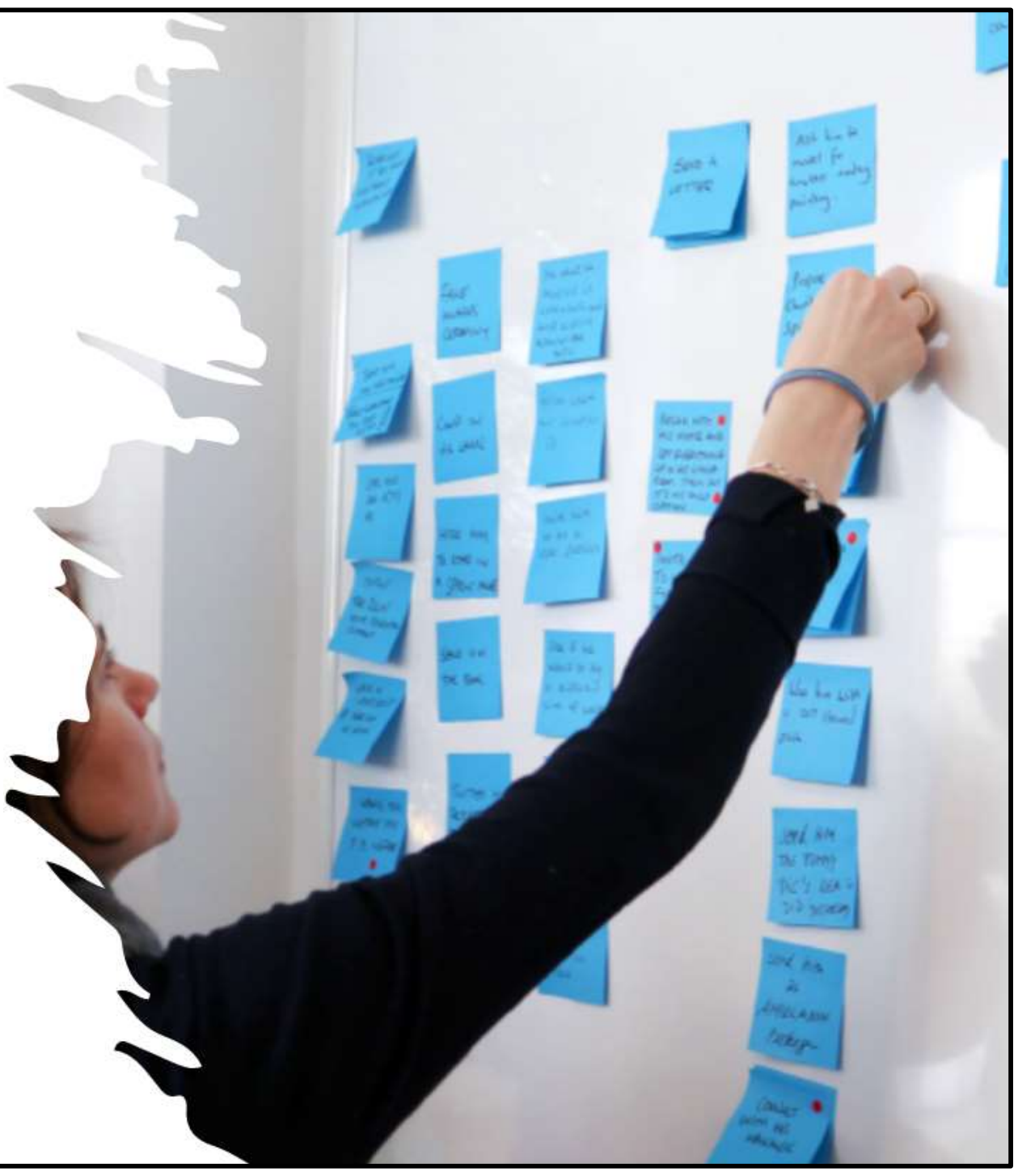

- HANDOUT STICKY DOTS – three per person
- Now everyone votes on the problems they consider to be the biggest issues. Group members can vote on their own sticky note and can put more than one dot on a problem of they feel strongly about it.
- DUPLICATES – if anyone sees a duplicate place the duplicates one on top of the other and add dots to the to most sticky note
- **<u>Do this individually and without discussion (3 mins)</u>**
- Table facilitators reorganize the sticky notes in order of priority

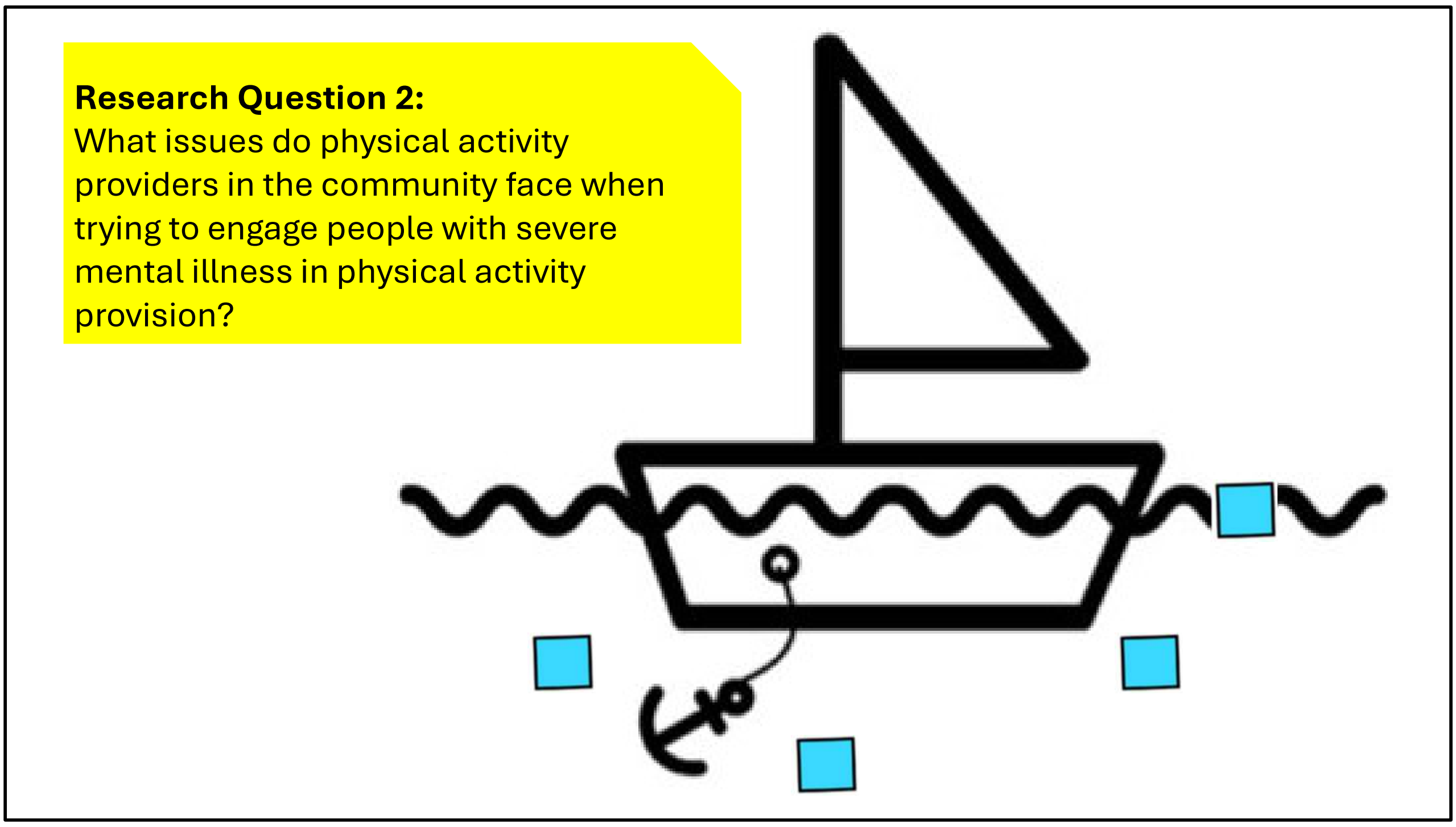

- Repeat the previous task, but with our second question
- Explain themes generated from WS1 and WS2
- Facilitator to describe these and add a sticky note (one for each theme/problem) to the main board. Table facilitators to add them to the table boards on.
- Group to write on a sticky notes any other issues they feel add to the problem – one sticky note per problem,
- **<u>No discussion at this stage, everyone works together alone (5 mins)</u>**
- Group to quickly and quietly place their sticky notes on to their table's board – still no discussion (1 minute)

Prioritise the problems

- 3 dots per person
- Vote on the problems you consider to be the biggest issues.
- You can vote on their own sticky note
- You can put more than one dot on a problem of you feel strongly about it.
- DUPLICATES – if you sees duplicates place them on top of each other; make sure all the dots are on the top-most sticky note
- 3 minutes

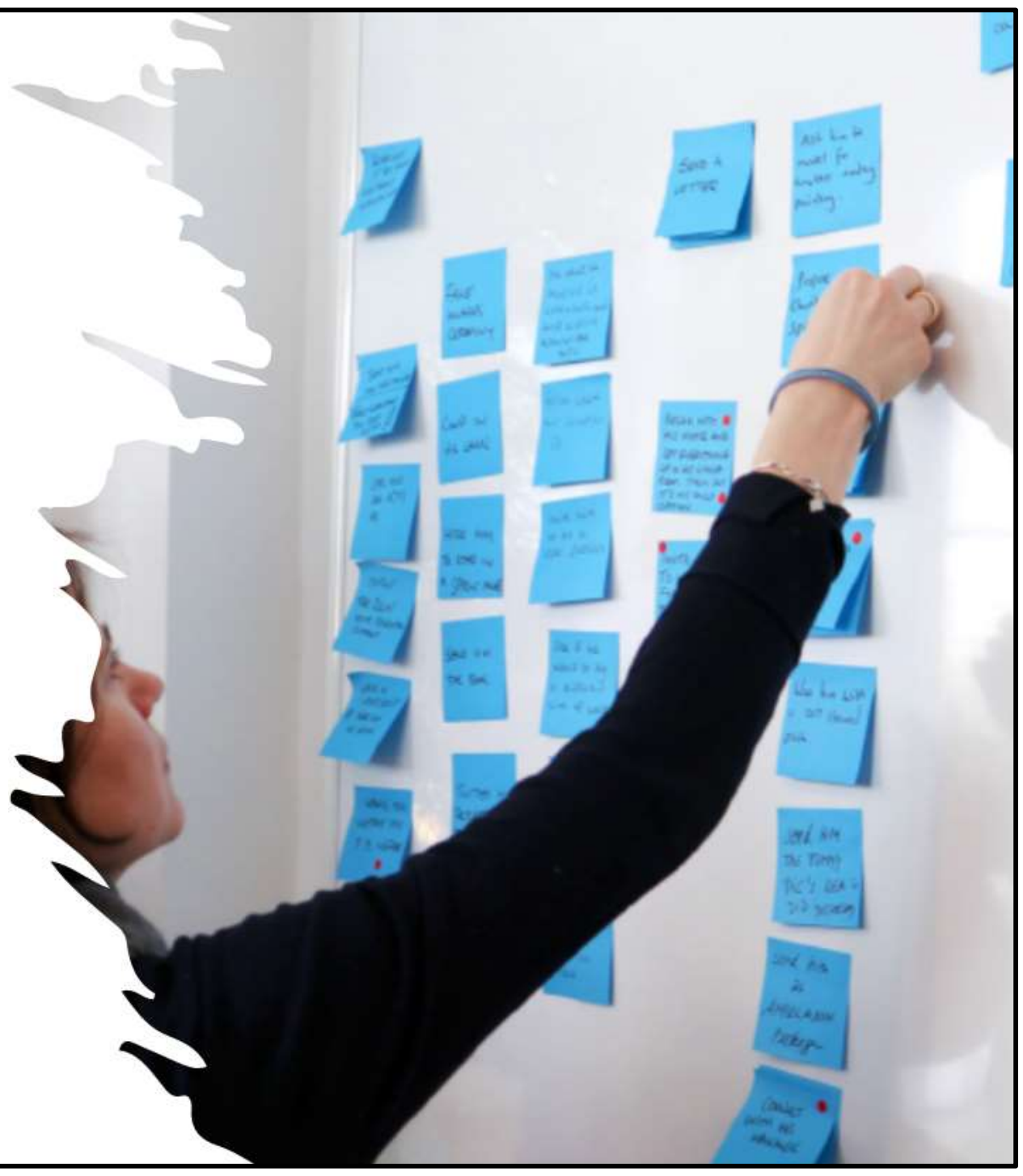

- HANDOUT STICKY DOTS – three per person
- Now everyone votes on the problems they consider to be the biggest issues. Group members can vote on their own sticky note and can put more than one dot on a problem of they feel strongly about it.
- DUPLICATES – if anyone sees a duplicate place the duplicates one on top of the other and add dots to the to most sticky note
- **<u>Do this individually and without discussion (3 mins)</u>**
- Table facilitators reorganize the sticky notes in order of priority

Prioritise the problems

- Table facilitators please take the problems voted on and arrange them in order of priority.

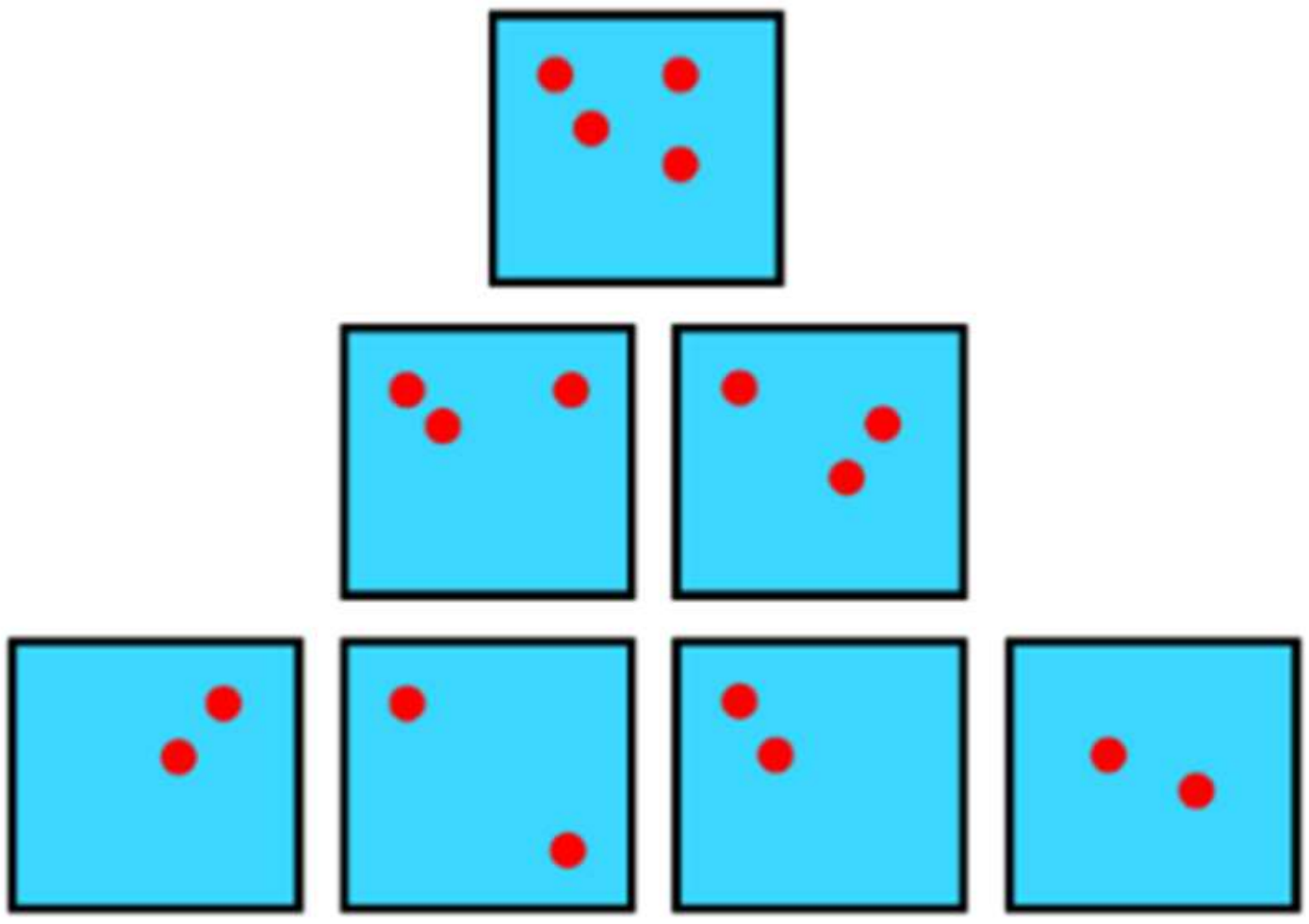

- Table facilitators reorganize the sticky notes in order of priority
- We will come back to these shortly

Lunch

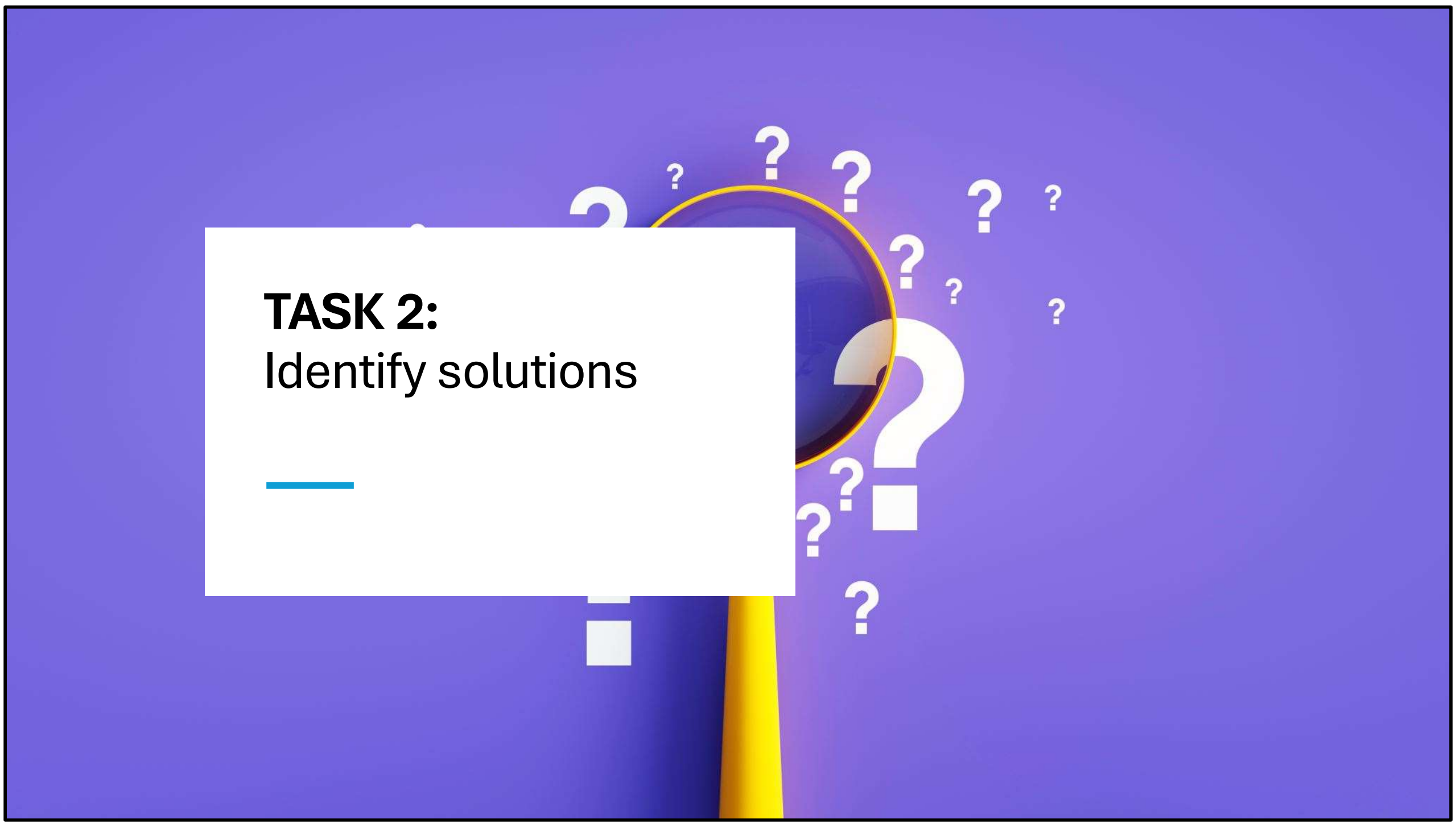
TASK 2:
Identify solutions

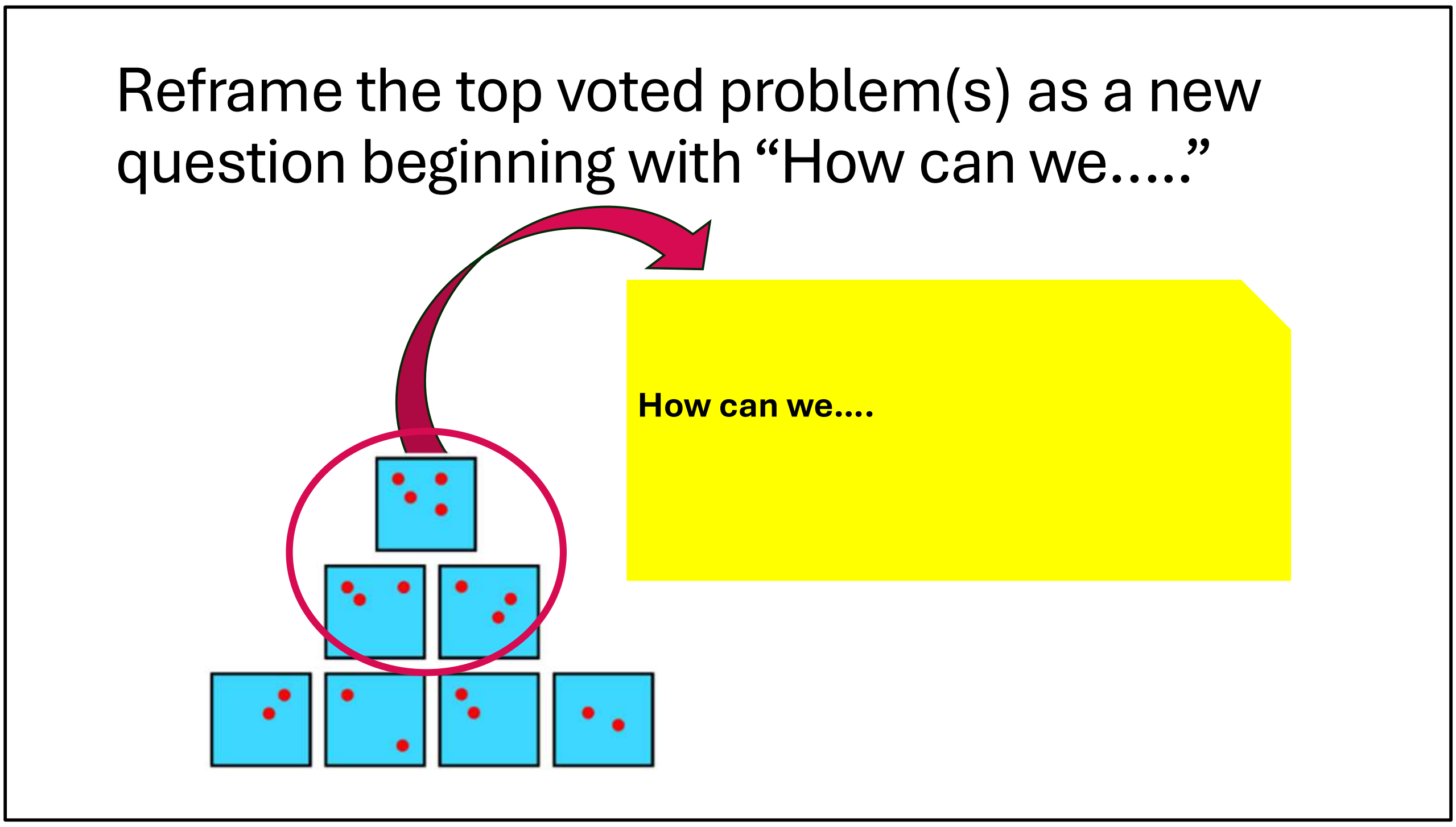

- **The table facilitators reframes the top-voted problem on their table, as a challenge or question “How can we....”** This helps the group produce a mass of solutions
- Example
  - The top voted sticky notes says "I have no idea what's happening on project X"
  - “How can we.... keep everyone in the loop on all current projects”

- If you’d like to find solutions to more than one challenge, repeat this step for all prioritised negatives.
- Work your way down from the problem with the highest number of votes, turning each of them into a separate “How can we…” questions.
- Be aware that the more challenges/questions the group works on, the more you dilute the group’s focus on the highest-voted negative.
- It is recommended that you ignore all negatives with just one vote and having a maximum of 4-5 challenges.
- Any problems you decide not to solve now can go into a problem backlog and can be looked at on another day. Once you have rephrased all negatives as HCWs, continue with the next task.

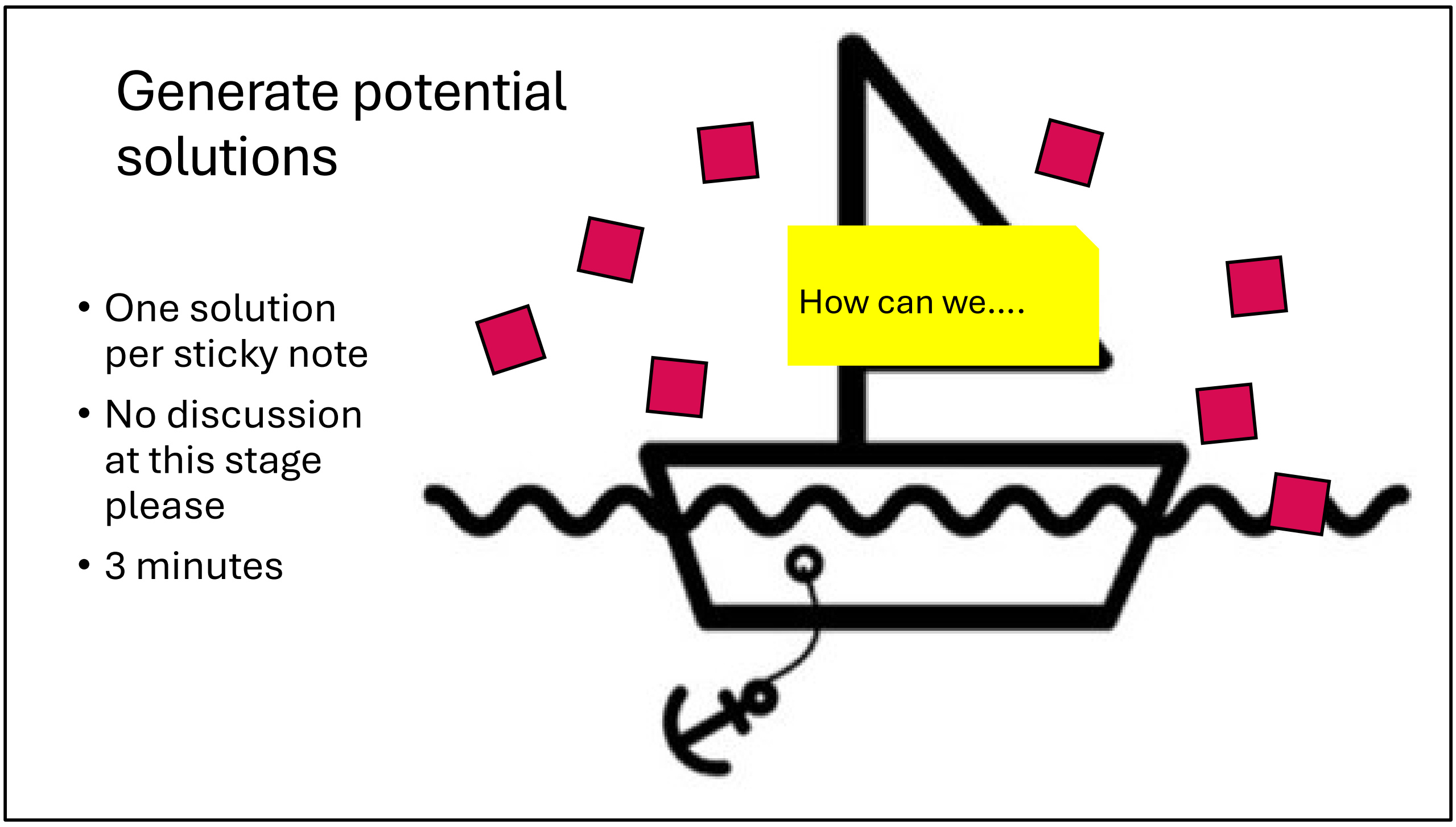

- Explain 'solution' themes generated from WS1 and WS2
- Facilitator to describe these and add a sticky note (one for each theme/problem) to the main board. Table facilitators to add them to the table boards.
- Group to write solutions they can think of on a sticky note (one sticky note per problem),
- **<u>No discussion at this stage, everyone works together alone (5 mins)</u>**
- Group to quickly and quietly place their sticky notes on to their table's board – still no discussion (1 minute)

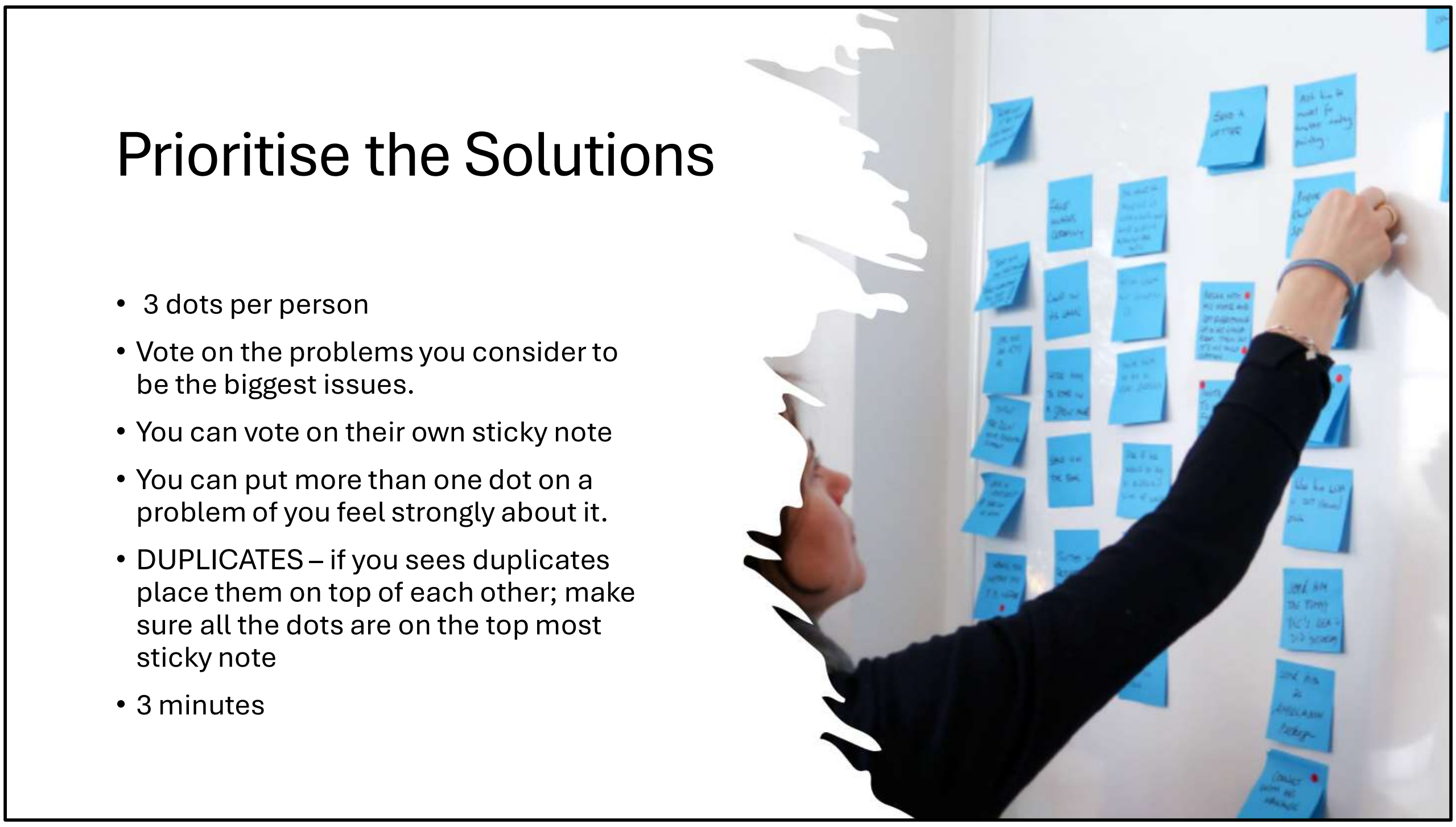

- HANDOUT STICKY DOTS – three per person
- Now everyone votes on the problems they consider to be the biggest issues. Group members can vote on their own sticky note and can put more than one dot on a problem of they feel strongly about it.
- DUPLICATES – if anyone sees a duplicate place the duplicates one on top of the other and add dots to the to most sticky note
- **<u>Do this individually and without discussion (3 mins)</u>**
- Table facilitators reorganize the sticky notes in order of priority

Prioritise the Solutions

- Table facilitators please take the solutions voted on and arrange them in order of priority.

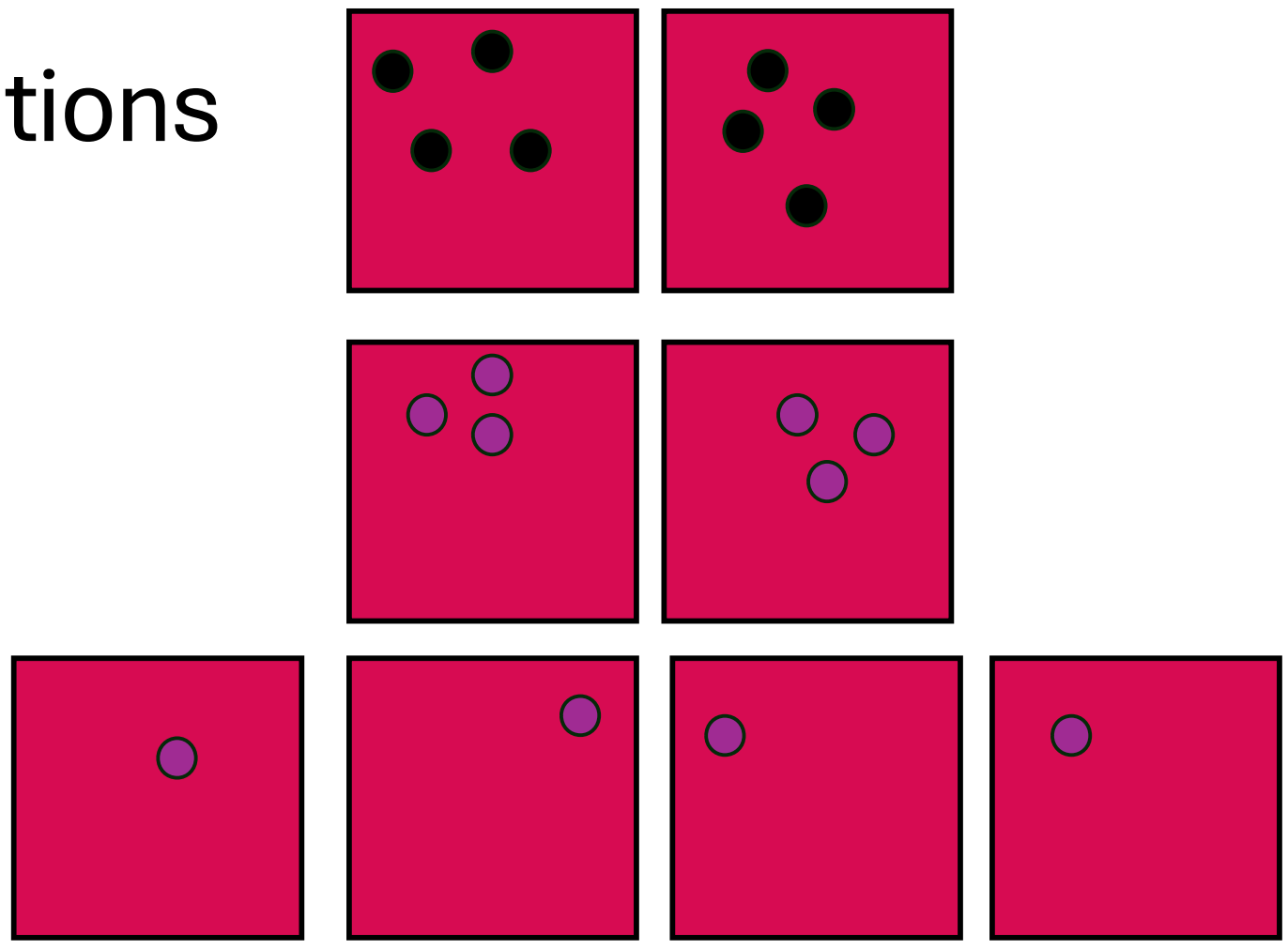

- Table facilitators reorganize the sticky notes in order of priority
- **REAPEAT TASK 3 FOR EVERY ‘HOW CAN WE…’ QUESTION**

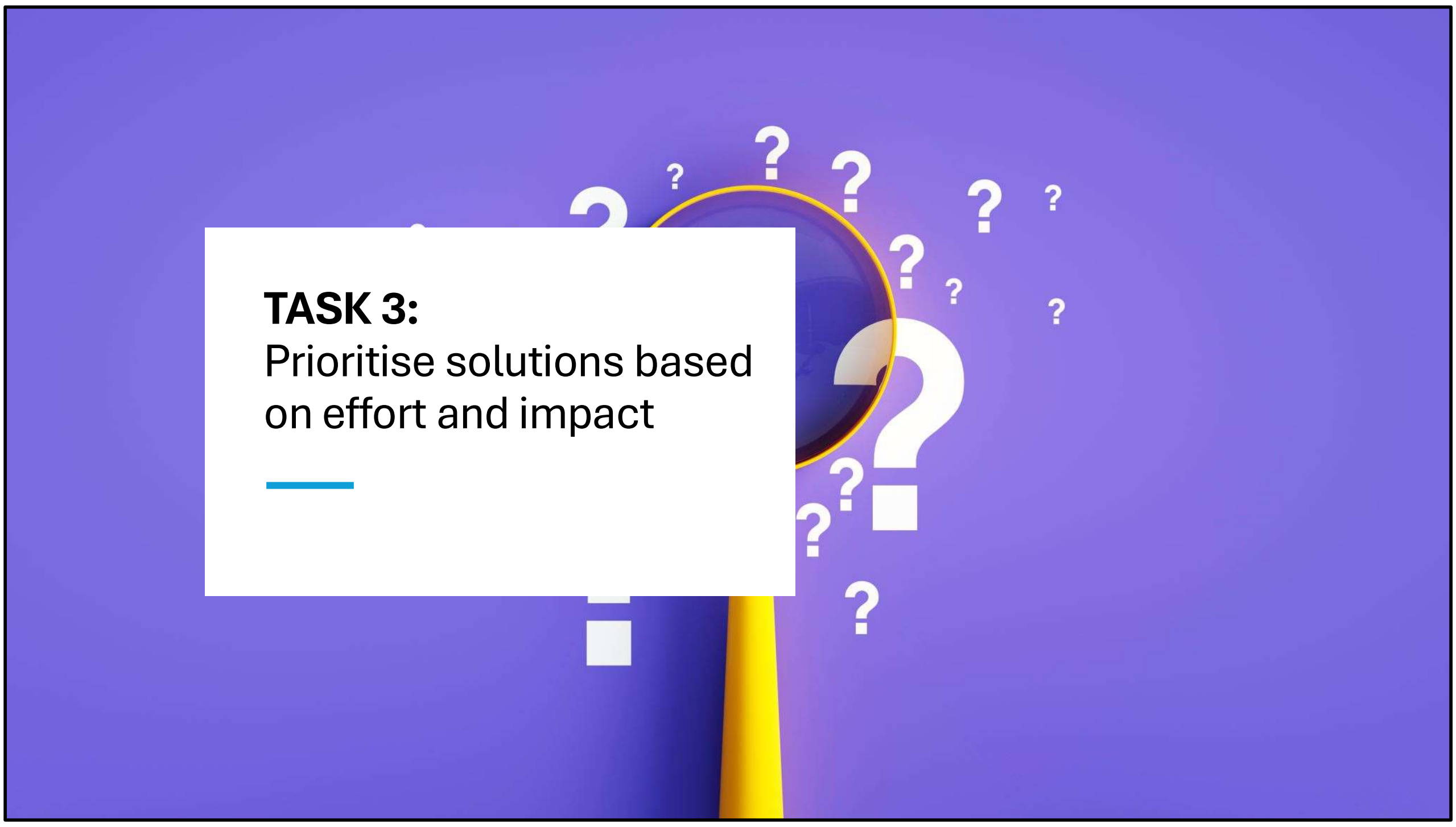
TASK 3:
Prioritise solutions based on effort and impact

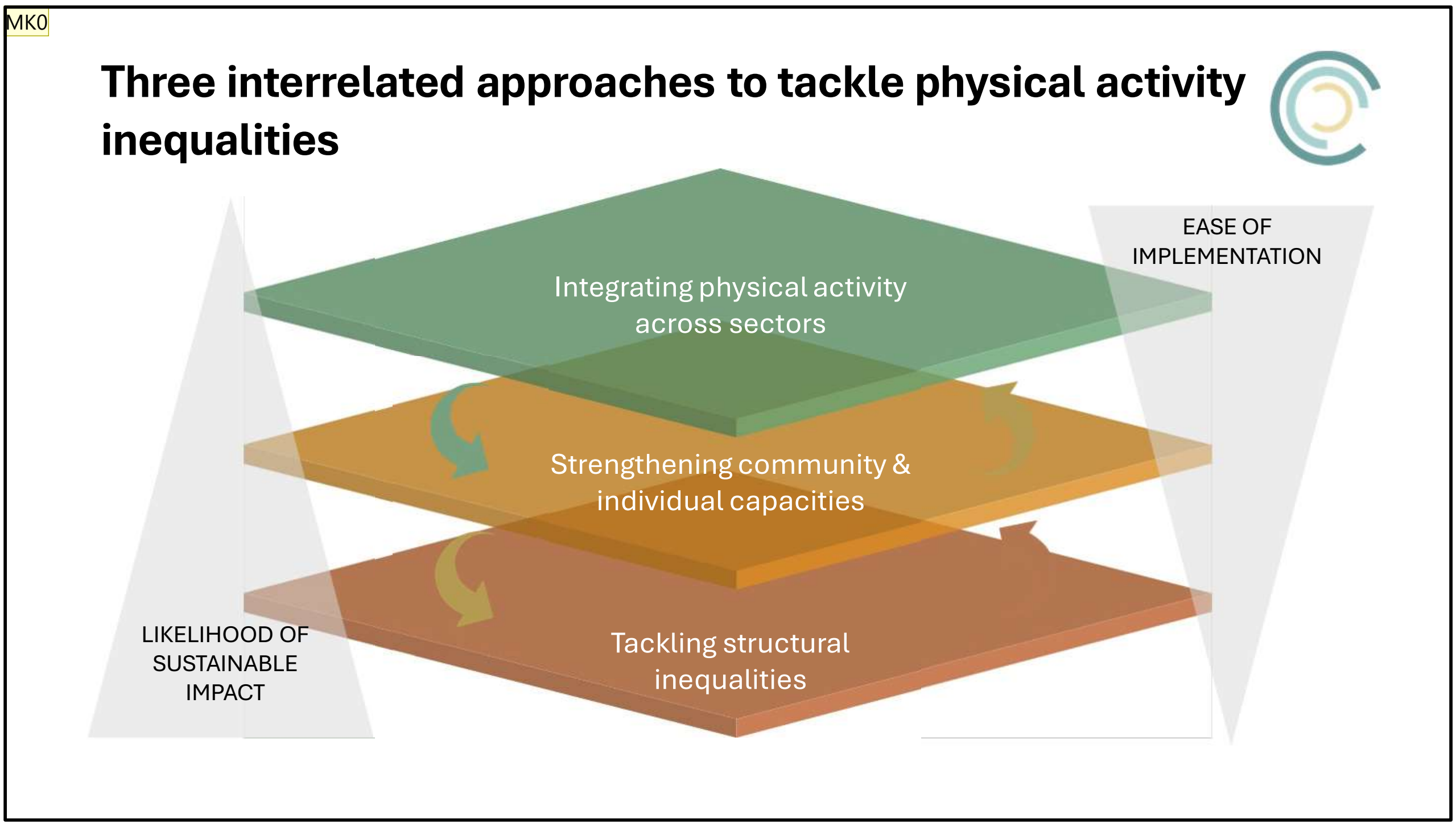

Talk through 3 different approaches very briefly.

How do we act in these ways

What we have observed in work of PBA and QSA, dependant on who is leading the work is how they interpret how they can act in the system, is different. 3 different practices are see in the work… elements of all of it and it is interconnected.

Slide 24

MK0 It would be great to use simple language to ensure all participants can understand this slide. Also, it would be helpful to include definitions for terms like "structural inequalities."

Since this is a co-design workshop, I suggest removing this slide. Explaining its content could take a while, and we might be better off dedicating more time to hearing participants' thoughts and ideas.

Machaczek, Katarzyna, 2025-05-01T10:02:48.667

Take the top voted solutions only and place them on the effort/impact scale

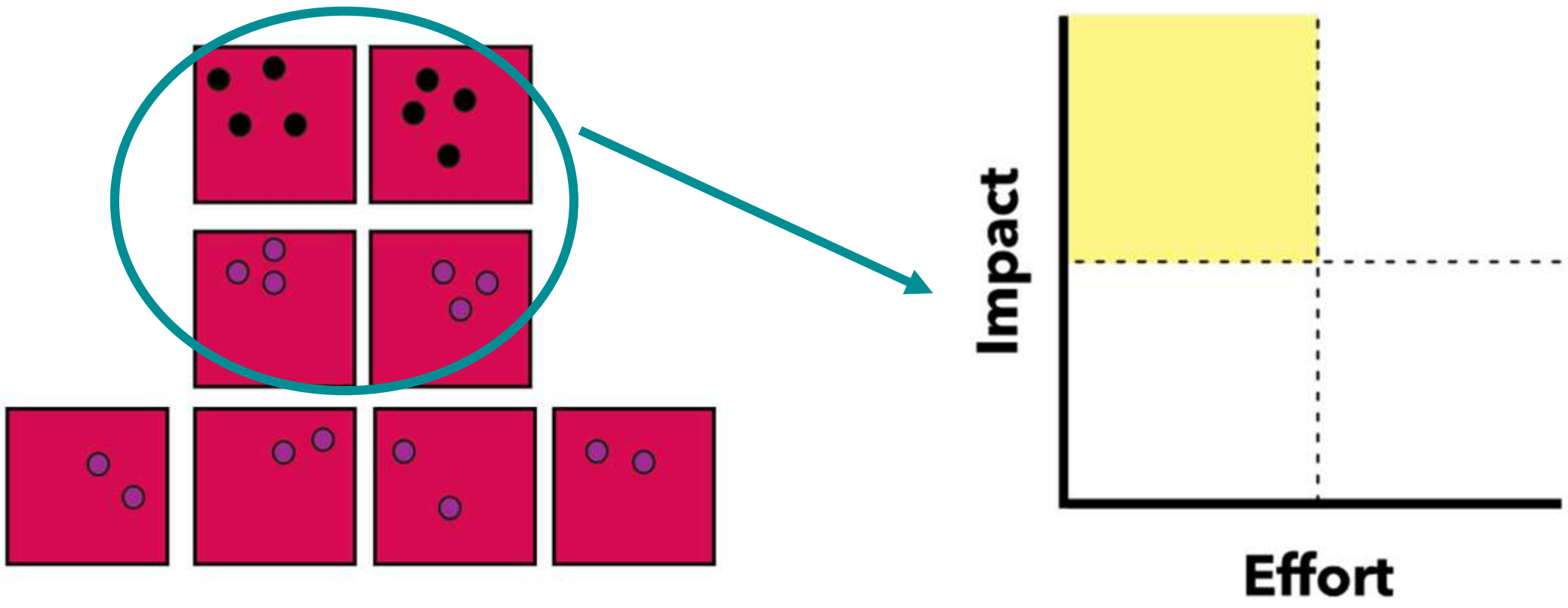

- Now it's time to decide which solutions offer the biggest "return on investment" with the least amount of time and effort.
- To ensure we utilise our time efficiently, we will use the Effort/ Impact scale to determine which solutions we should implement right away, which should be turned into a bigger project, and which we should shelve for now.
- The horizontal "Effort" axis along the bottom edge estimates how much time, work, resources, cost we need to implement the idea (from "very little" to "a lot").
- The vertical "Impact" axis along the left edge is the magnitude to which we think the solution would fix our problem (from "not at all" to "completely"). Consider whose impact/effort - NHS, CP, Lived experience people?
- The yellow area defines what is realistic for Co-SPACES
- As a table facilitator, you need to be ready to step in and manage the group at this point, as this is the moment when discussion tends to open up. The table facilitator now takes each solution, starting with the highest voted one, and adds them to the Effort/ Impact Scale.

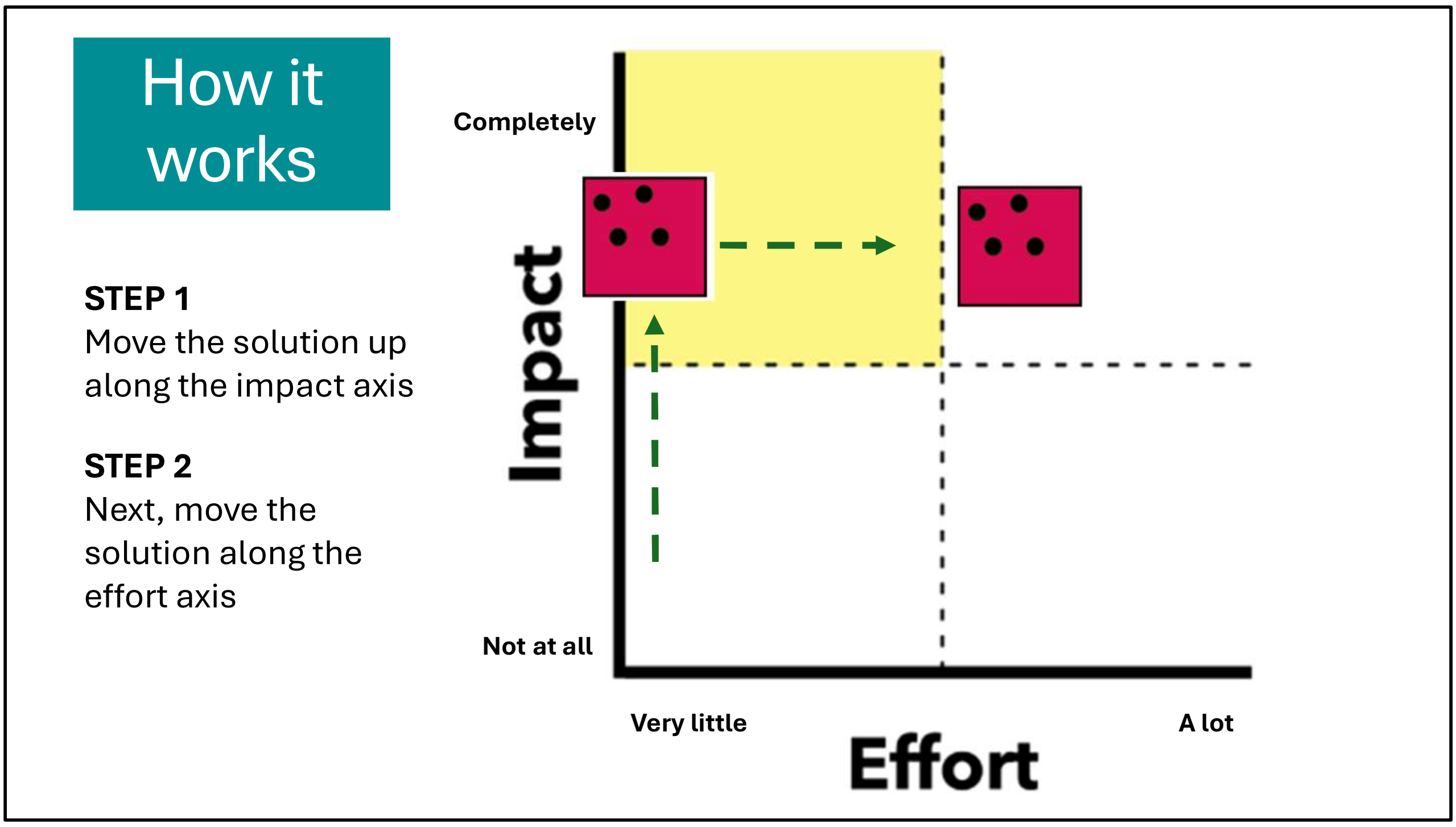

Here's how it works (Main facilitator to demo on the board with their doted sticky notes)

1. Start with "Impact":
    - Take the sticky note with the top-voted solution…
    - … hover it over the centre of the Effort/ Impact Scale…
    - … and simply ask "is the impact higher or lower?"

2. The facilitator then moves the sticky note up or down the "Impact" axis until the team members are happy with the placement. Often some small discussions break out here, so the moderator has to be diligent in finding a consensus and stopping any conversations extending past 20 seconds. The placement on the axis doesn't have to be perfect, an approximation is fine. The important thing is what quadrant the sticky note is in!
3. Once the impact has been determined, use the same method for "Effort", asking "Is the effort higher or lower?" Move the sticky note left or right, until the group is happy with the placement.
4. Repeat the process for the other top-voted solutions Once the top voted sticky-notes have been added to the scale, you'll have something that looks like this…. Main facilitator to demo on the board with their doted sticky notes

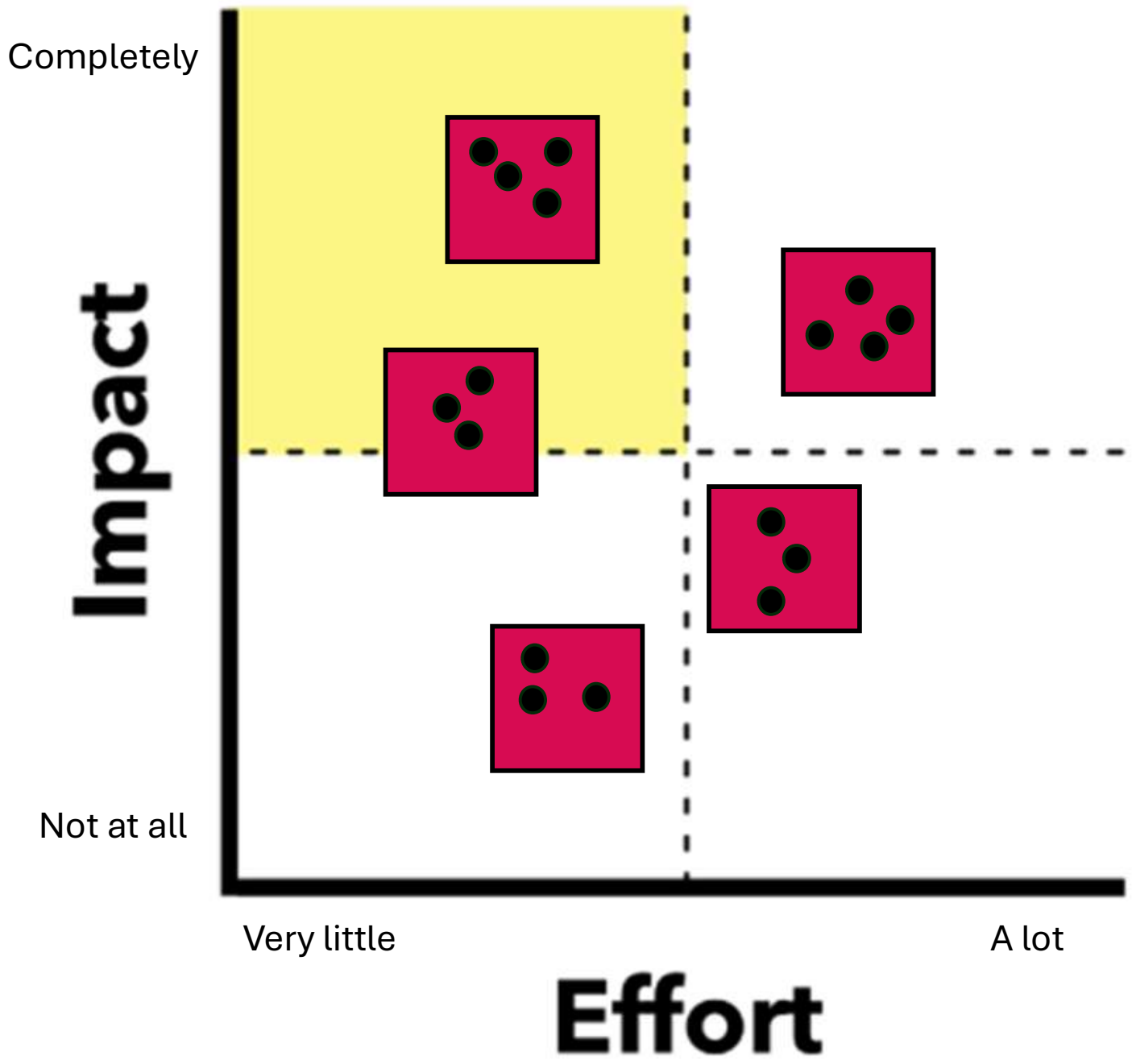

- When you look at the yellow “sweet spot” on the top left, you have a clear view on which high impact solutions can be executed on and tested very quickly.
- On the top right quadrant, you can see high impact solutions that will take more time and effort.

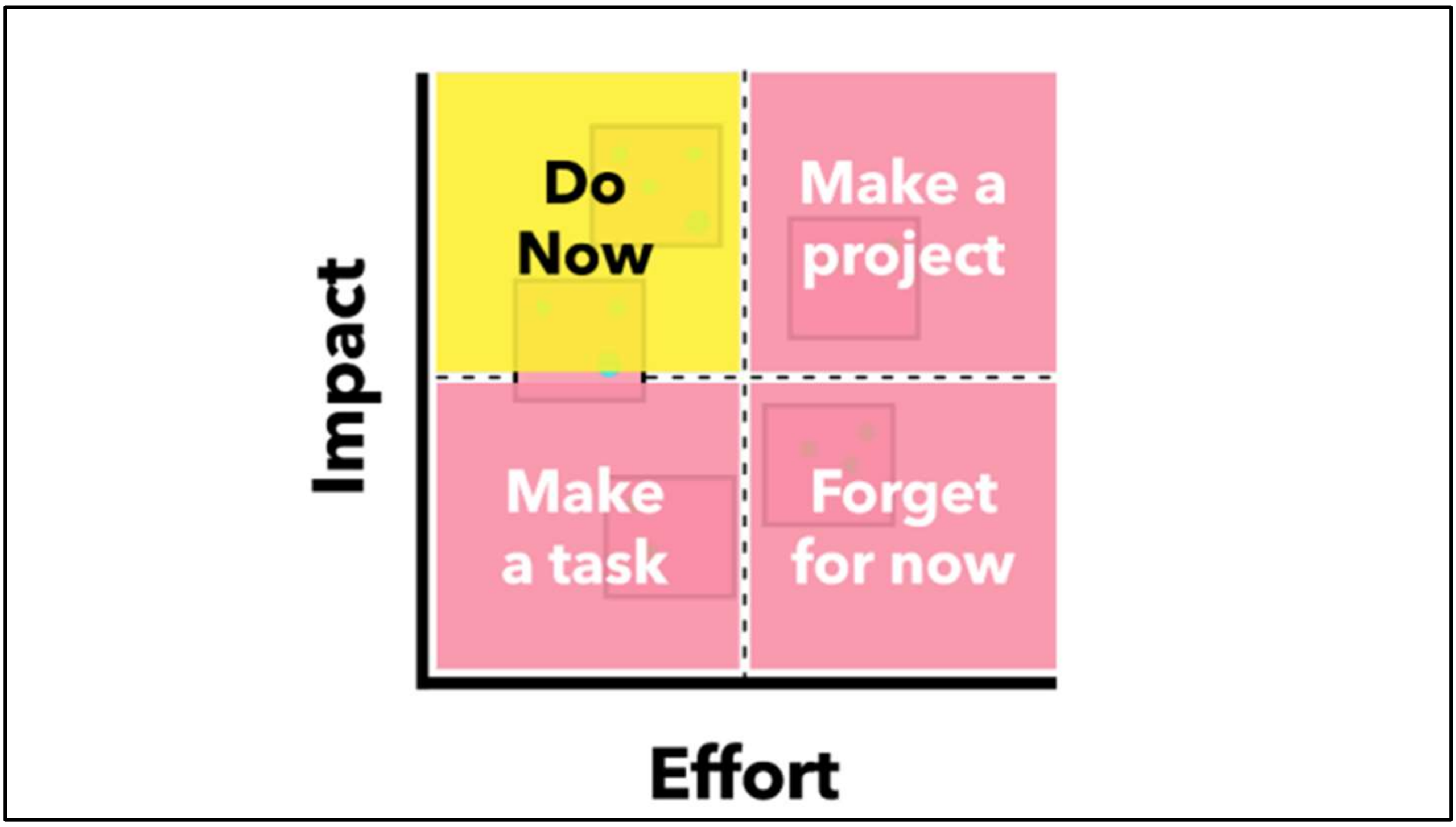

- Explain the quadrants after you have completed the exercise so that participants are not swayed to move into one direction over an other

Break

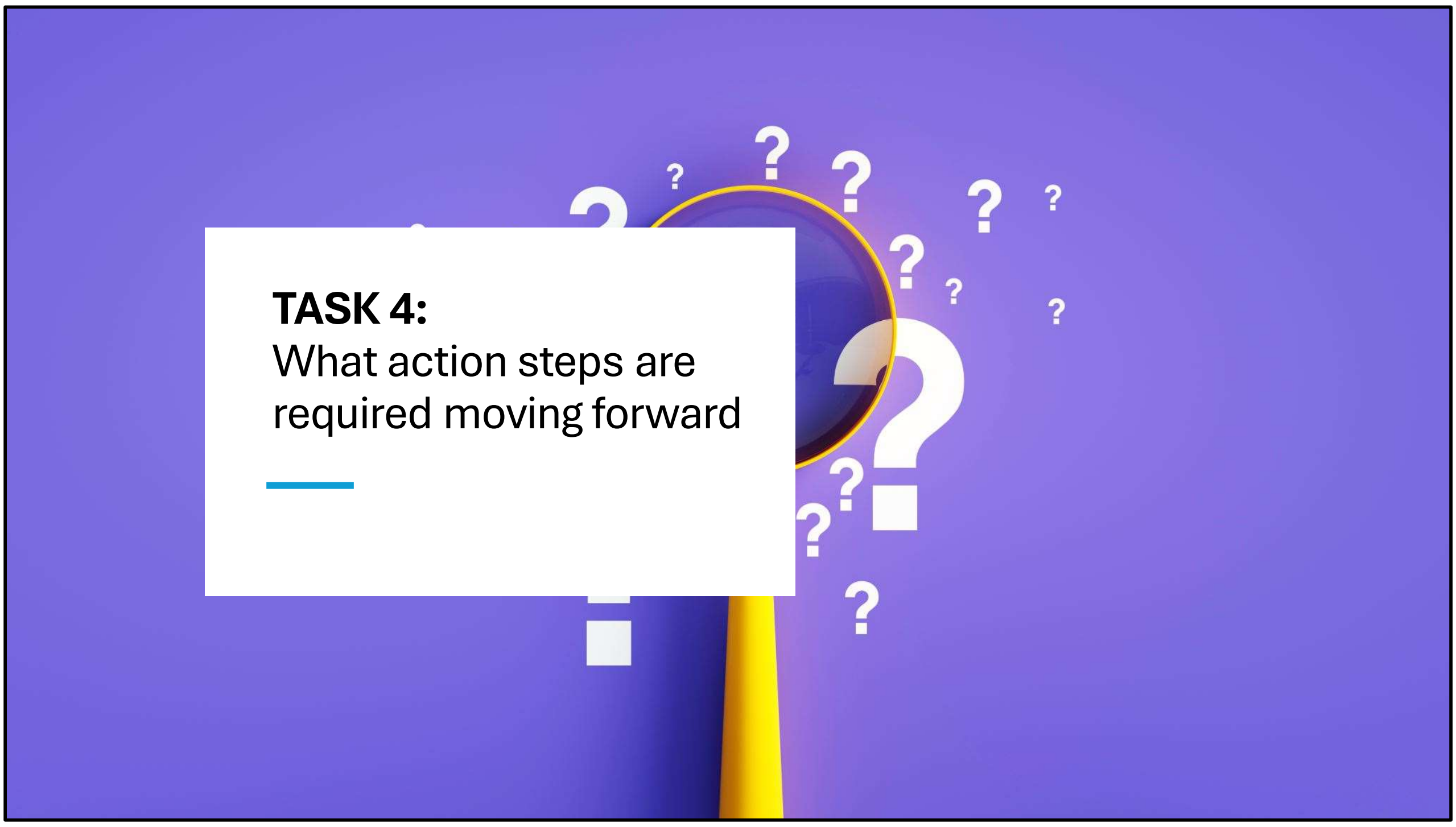
TASK 4:
What action steps are required moving forward

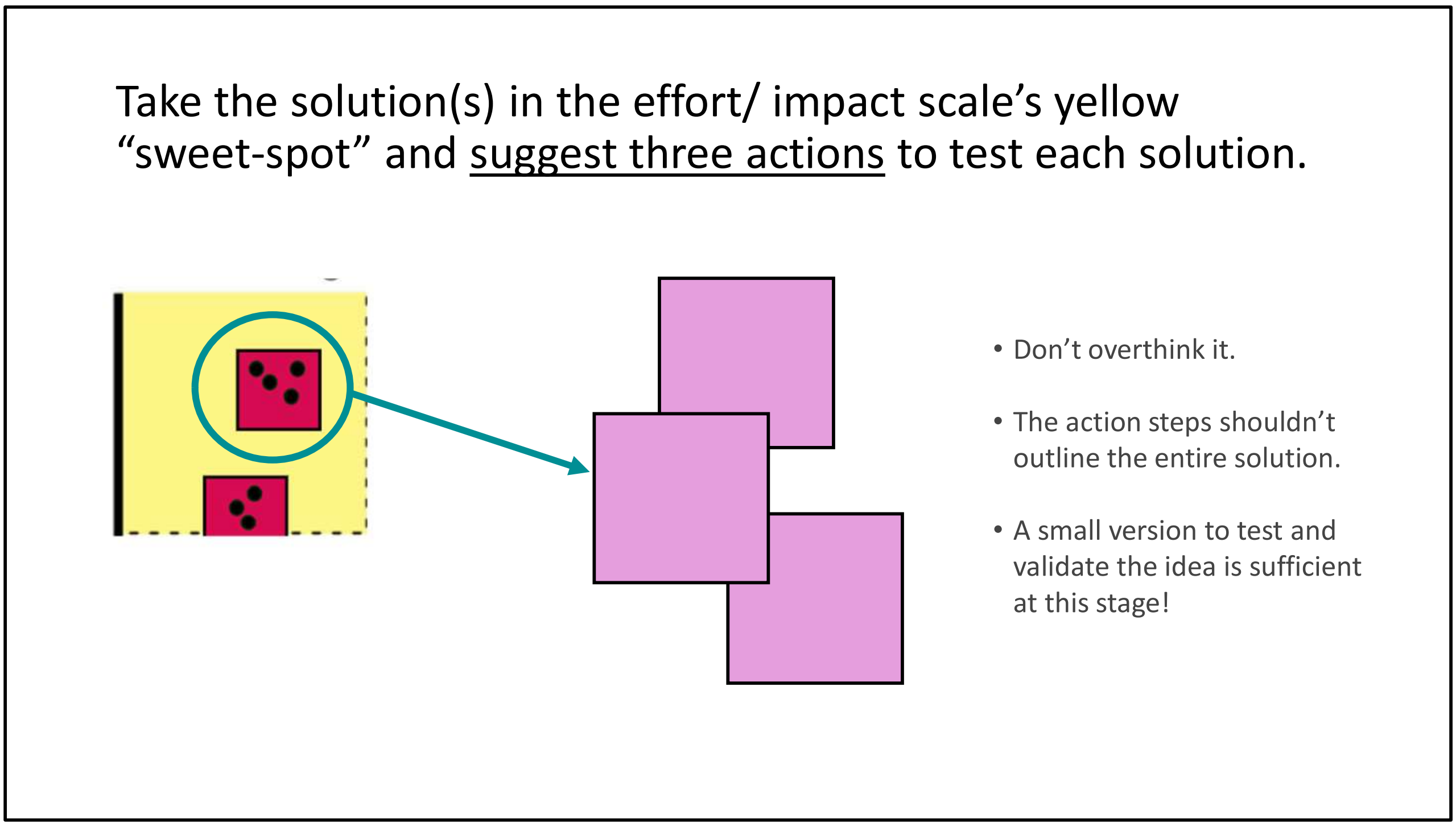

- The table facilitators now take the solutions in the Effort/ Impact Scale's "sweet-spot" quadrant and asks the team to suggest three action steps for testing the solution.
- Each participant can write their three action steps on individual sticky notes
- The group decide on the key steps through voting using their sticky dots.

    - Don't overthink it.
    - The action steps shouldn't outline the entire solution.
    - A small version to test and validate the idea is sufficient at this stage!

Prioritise your action points

- 3 dots per person
- Vote on the solutions you consider may be useful
- You can vote on their own sticky note
- You can put more than one dot on a problem of you feel strongly about it.
- DUPLICATES – if you sees a duplicate place them on top of each other and add your dot
- 3 minutes

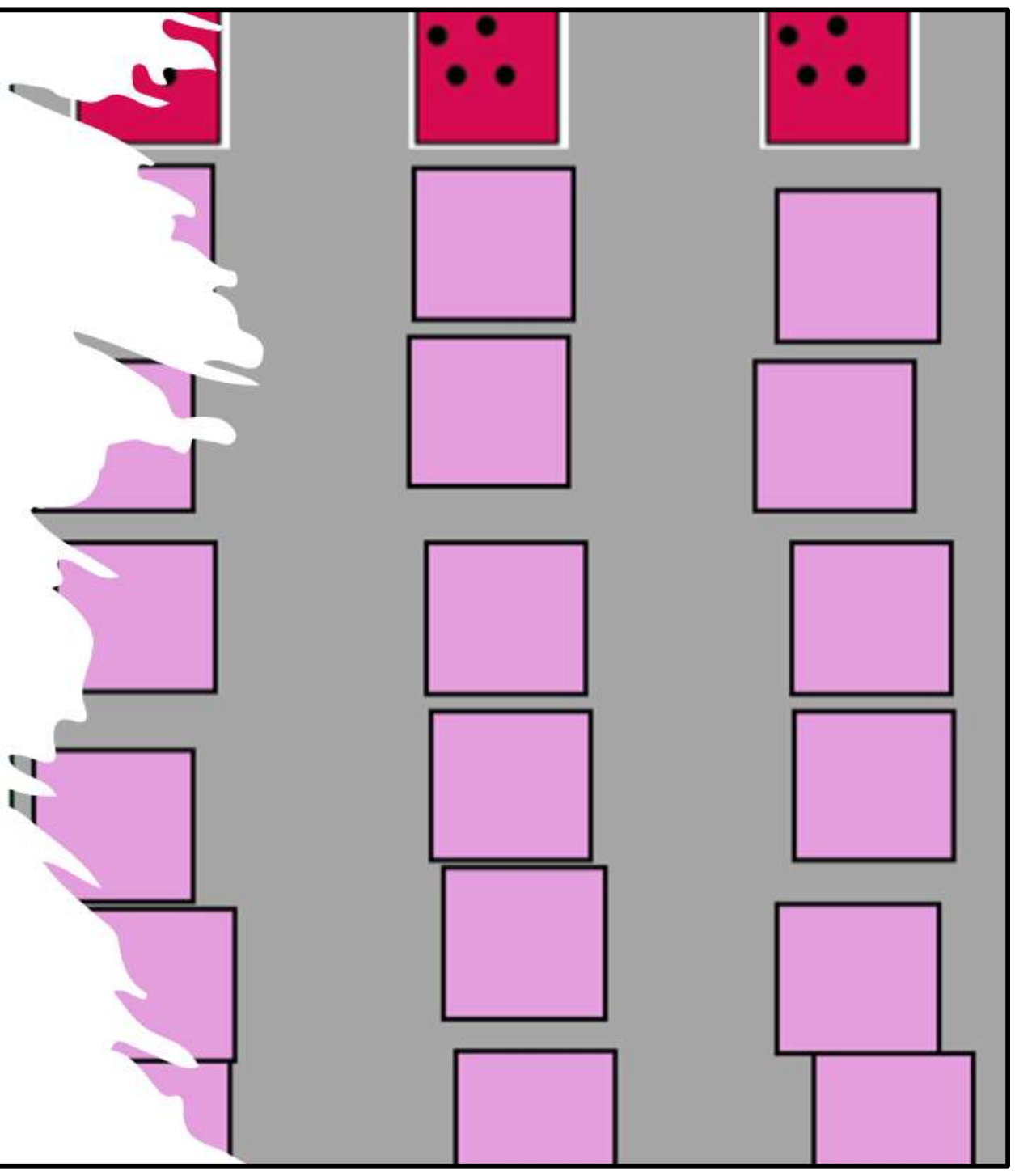

- HANDOUT STICKY DOTS – six per person
- Now everyone votes on the problems they consider to be the biggest issues. Group members can vote on their own sticky note and can put more than one dot on a problem of they feel strongly about it.
- DUPLICATES – if anyone sees a duplicate place the duplicates one on top of the other and add dots to the to most sticky note
- **<u>Do this individually and without discussion (3 mins)</u>**
- Table facilitators reorganize the sticky notes in order of priority

Prioritise the Solutions

- Table facilitators please take the take the solutions voted on and arrange them in order of priority.

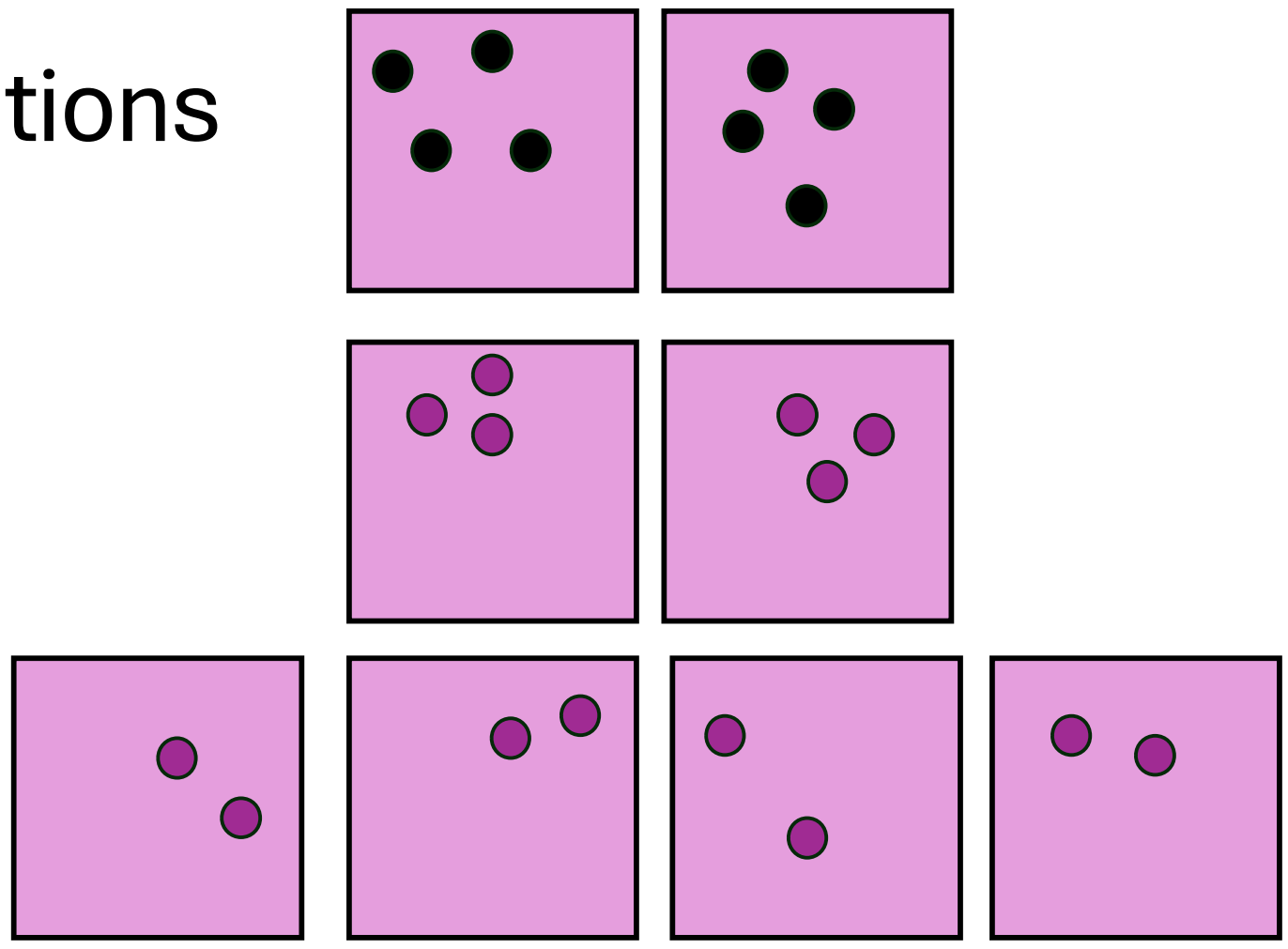

- Table facilitators reorganize the sticky notes in order of priority

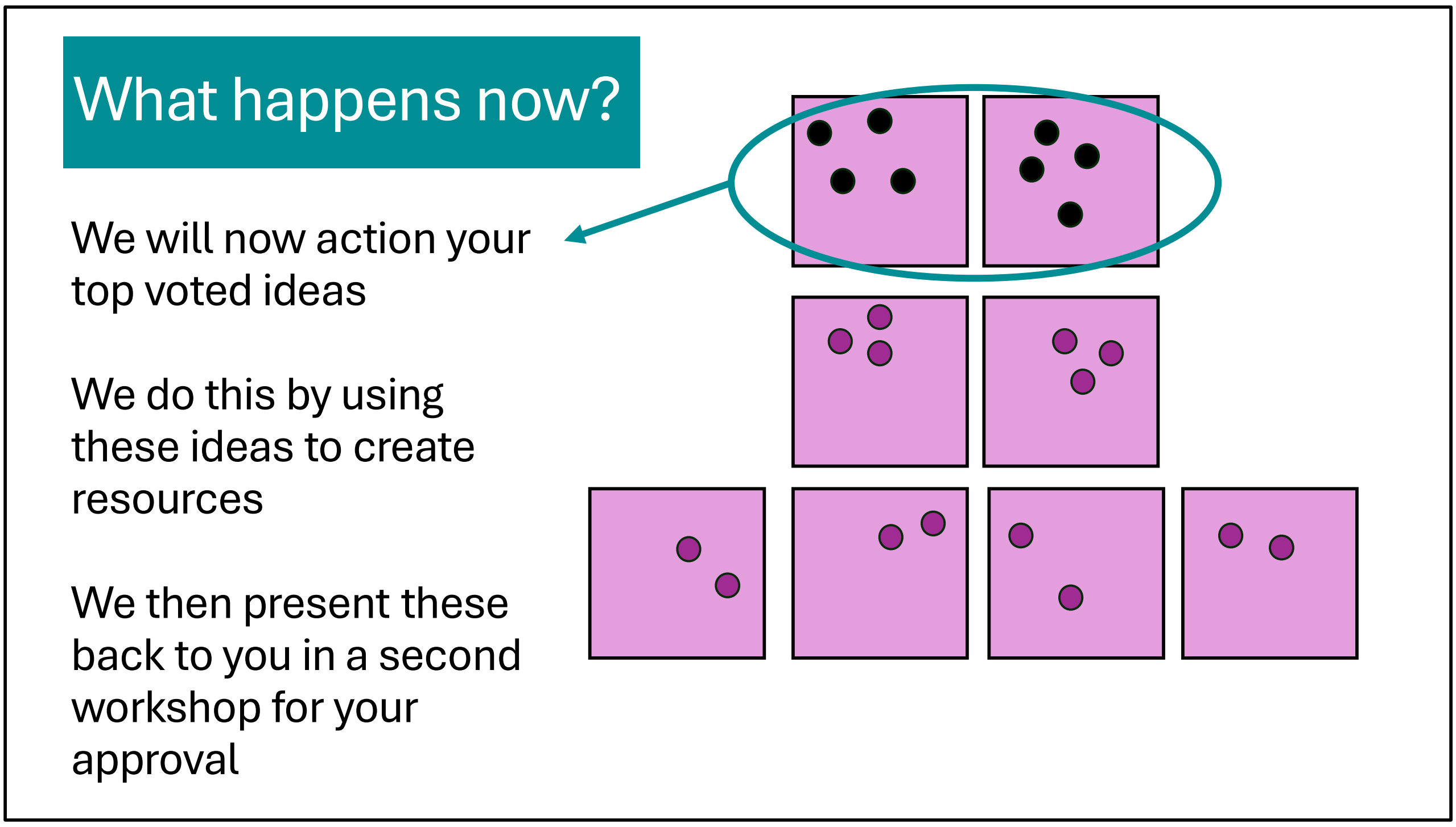

- We will now action your suggestions and present the outputs to you for consideration and approval in July
- Ask everyone to let Ann know if they wish to attend in July. Ann to tick them off on th eregister

THANK YOU

- Thank you so much for your time and effort in co-developing resources for both PACs and Community providers
- Hand over to Ann
    - Any expenses forms outstanding
    - Vouchers
    - Attending in July

- Please complete the feedback forms
- We will be staying on for a while afterwards if you have any questions
- THANK YOU AGAIN
