# Supplementary material for: Bridging the gap: co-producing resources for NHS mental health trusts and community providers to support people with severe mental illness live active lives in their community
Source: BMC Public Health. 2026 Jun 16;26:2418. doi: 10.1186/s12889-026-27936-7 (PMC13501637; doi:10.1186/s12889-026-27936-7)
Supplement: Supplementary file 3 [file 12889_2026_27936_MOESM6_ESM.pdf]

Co-SPACES
Co-Creation & Critique Workshop

Sheffield Health and Social Care NHS Foundation Trust | University of Leeds | University of Sheffield | York St John University | Prifysgol Bangor University | Sheffield Hallam University

Workshop 2 Agenda

11:00 –11:15	**Arrival and coffee**
11:15 –11:30	Recap of Co-SPACES aim and workflow
11:30 – 12:00	Results from workshop 1<br>Example resources from other studies / grey literature
12:00 – 12:30	**Lunch**
12:30 – 13:45	Task 1: Co-creation of resource content
13:45 – 14:00	**Break**
14:00 – 15:00	Task 2: Share, critique, and reflection session
15:00 – 15:30	Summing up and close

SPACES

A physical activity intervention integrated into NHS care co-developed with and for people with severe mental illness

Co-SPACES

Exploring the community aspect of supporting those with severe mental illness to be physically active in their community

SPACES

Supporting Physical Activity through Coproduction in pEople with Severe mental illness.

Co-SPACES project flow diagram

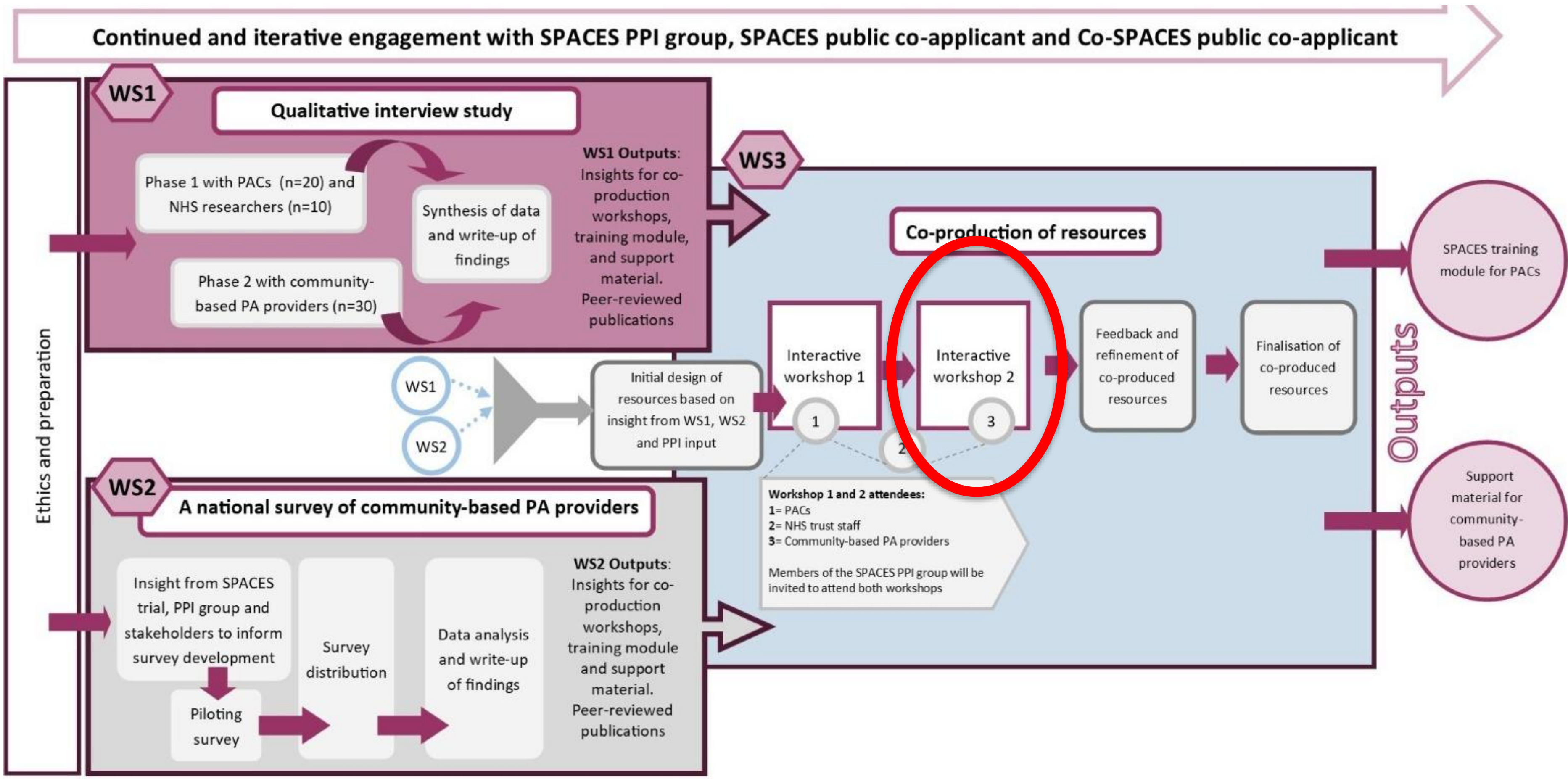

PA = physical activity; **PACs**= SPACES Physical Activity Coordinators; **PMG**= Co-SPACES Programme Management Group; **PPI**= patient and public involvement group; **WS**= workstream

Workshop 1

Question 1:
Why might a PAC struggle to identify and engage with community-based physical activity providers?

Question 2:
What issues do physical activity providers in the community face, when trying to engage people with SMI into their physical activity provision?

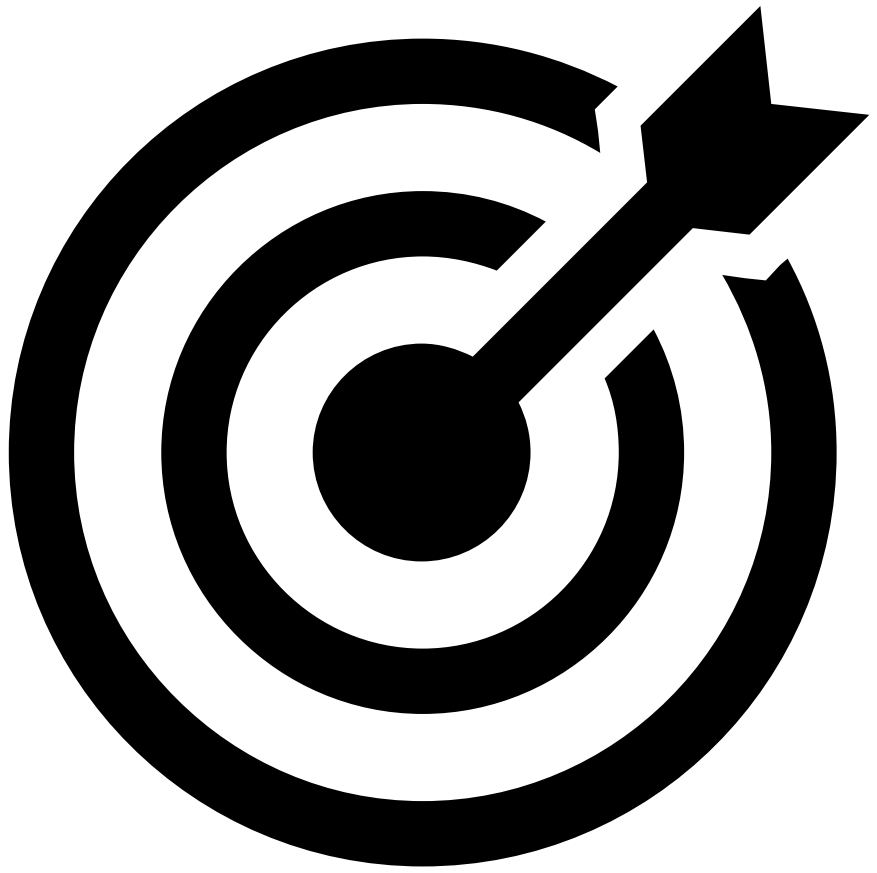

Co-SPACES aim:
How can we help them?

Results from workshop 1

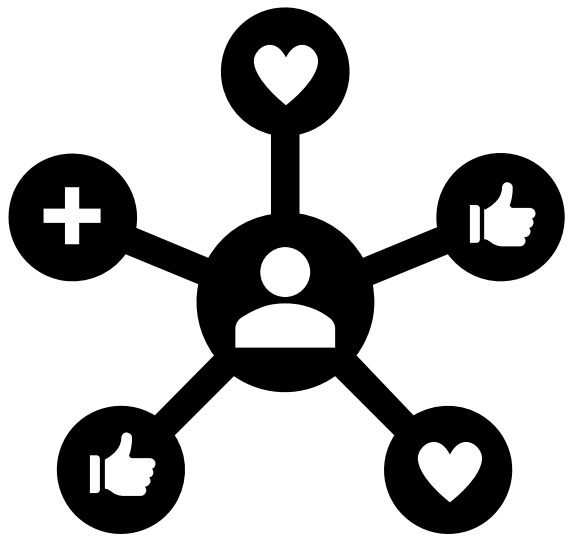
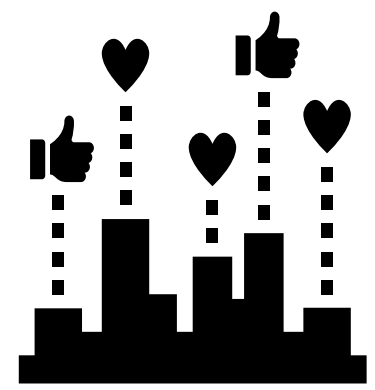
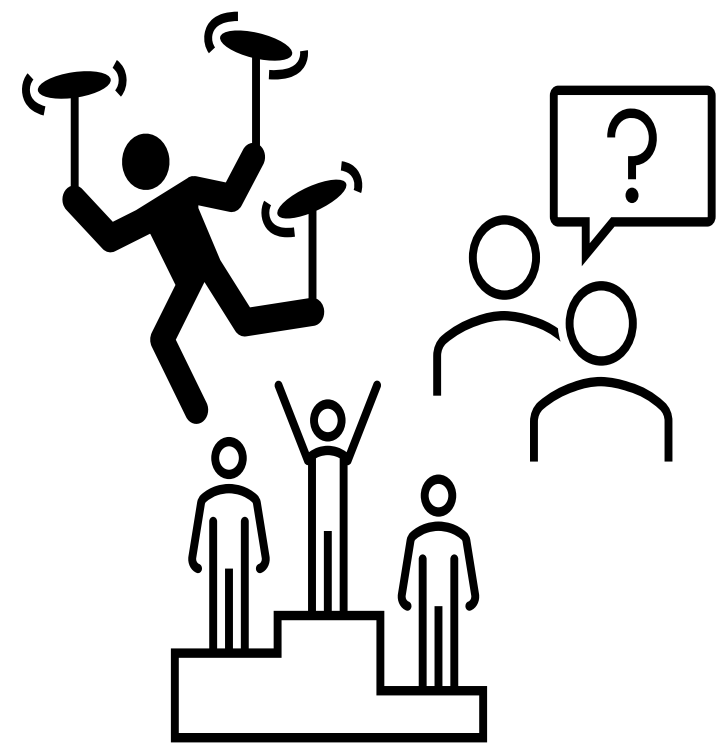

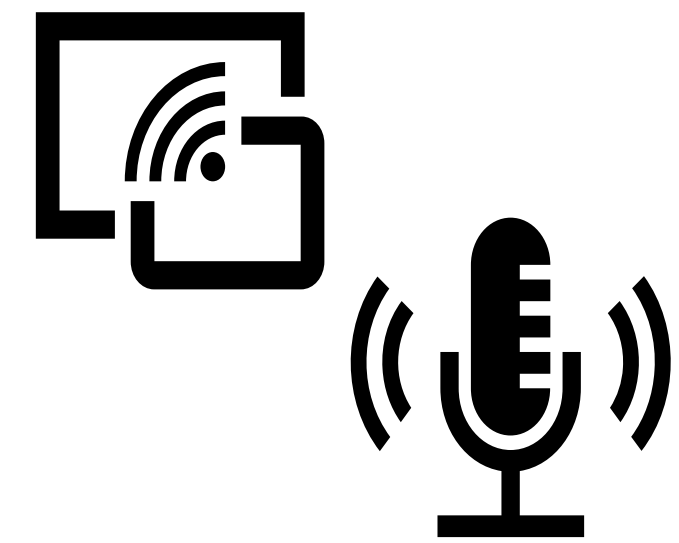

Update PAC training	Infographic<br>Venue use and circumnavigating challenges	Infographic<br>What is SMI, adaptions to PA sessions for CAPs	Video case studies + infographic applied aspects of delivering intervention at venue
Aimed at PACs	Aimed at PACs and CAPs	Aimed at CAPs and those with lived experience	Aimed at PACs, CAPs and those with lived experience
PACs updated 10th June and training slides updated ✓	Group A	Group B	Requires more time

Aim of Today's Workshop

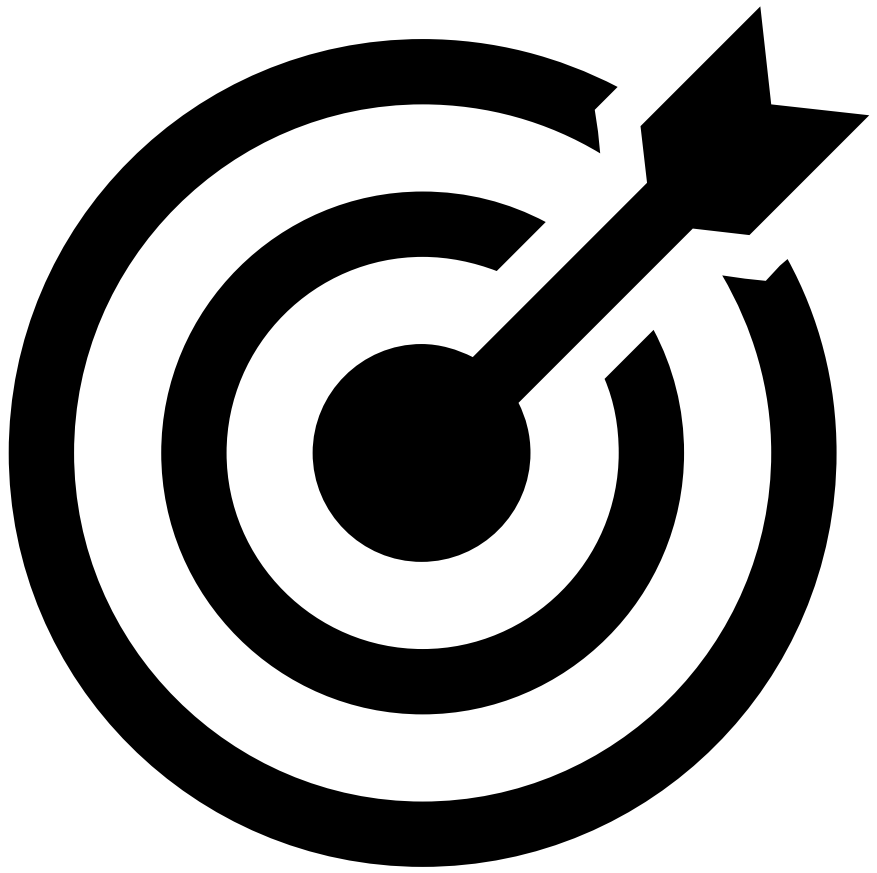

Co-SPACES aim:
How can we help them?

Group A: Creating SMI friendly venues

- Infographic
- Example venues and how different spaces have been used (e.g., green spaces).
- How to circumnavigate challenges,
- Case study of what's worked in the past

Group B: What is SMI & adapting your physical activity session to be SMI friendly

- Infographic for providers of what SMI is and how to adapt sessions etc.,
- Sign-post to existing training, list of MH training courses (e.g., Mind)

Example resources

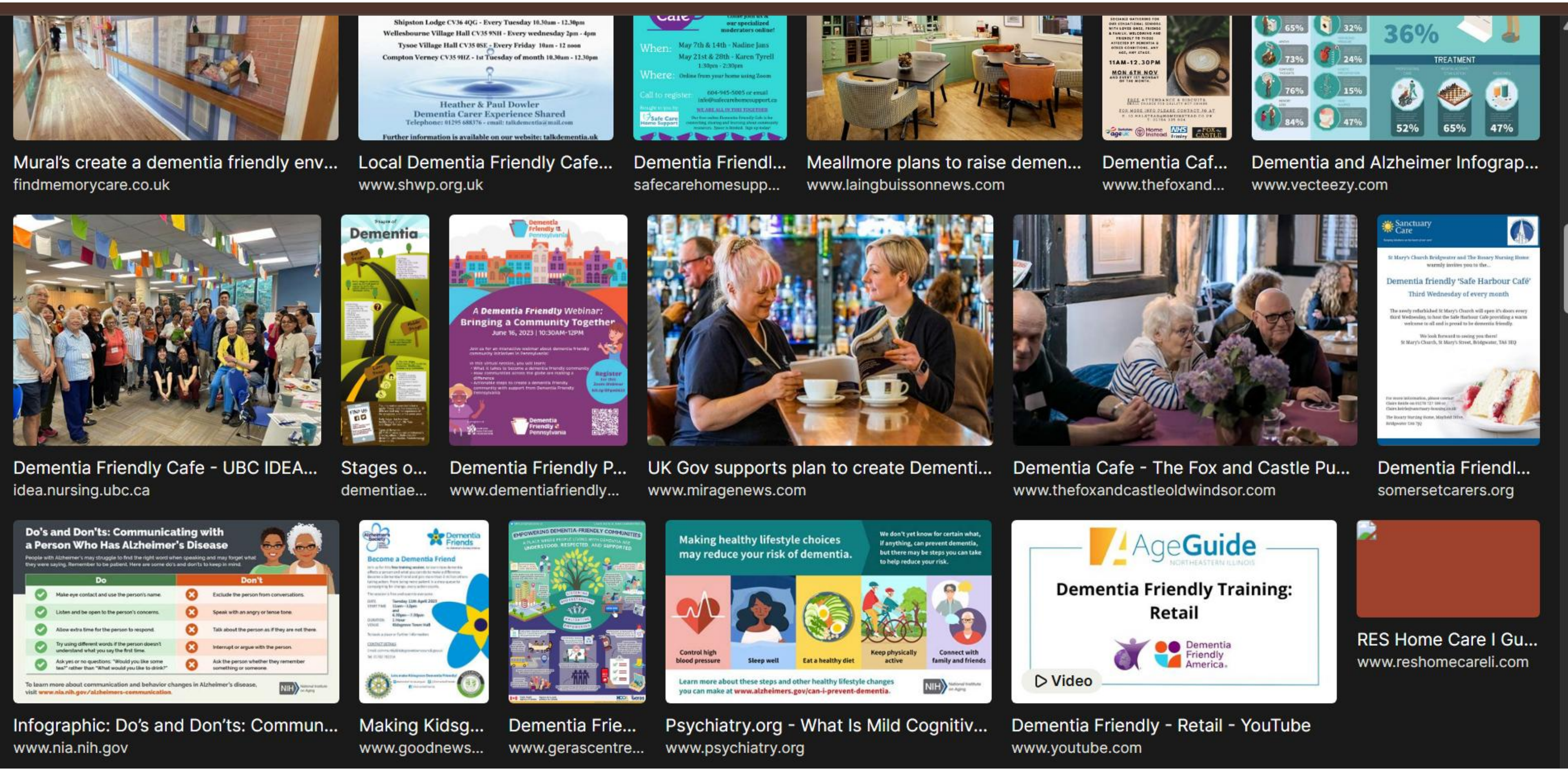

[Explore the web for inspiration]

Pre-task work: Design ideas – what do you like?

1

- Each group look at the examples provided

2

- What do you like in terms of presentation and content

3

- Work collaboratory and discuss your ideas for 15 minutes

Prompts to get going

- Take 10–15 minutes to explore the resources.
- Use the handout or sticky notes to jot down feedback.
- Prompt suggestions:
  - 'I like…'
  - 'I wish…'
  - 'What if…'

LUNCH

Task 1: Co-creation of resource content

In your groups...

1
- Use the web and your experience to consider content for the infographic.

2
- Sketch layouts, write sections, and/or design concepts

3
- Work collaboratory and discuss your ideas for 1 hour

4
- Prioritisation of key elements

Task 2: Share, critique, and revise

1

- Each group presents their ideas and mockup infographic

2

- Swap infographics and critique – use keep / add / drop / change, to guide your critique

3

- Work collaboratory and discuss your ideas for 45 minutes

4

- Prioritisation of key elements

Task 2: Reshare and reflect

- Each group presents revised versions or improvement ideas.
- Share for 5-10 minutes per group.
- Use your dots to prioritise key elements

Summing up

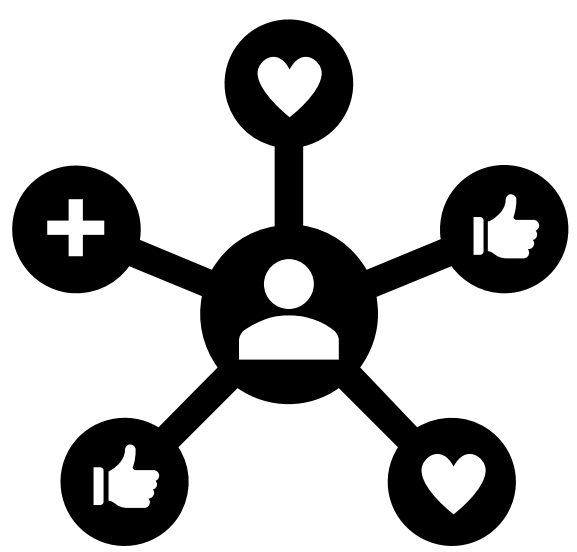

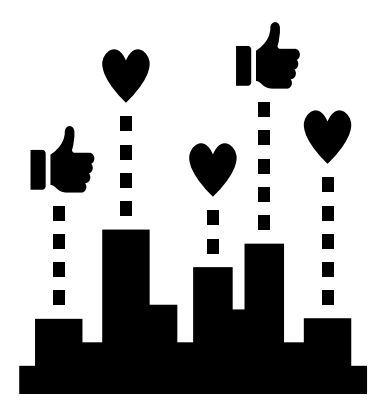

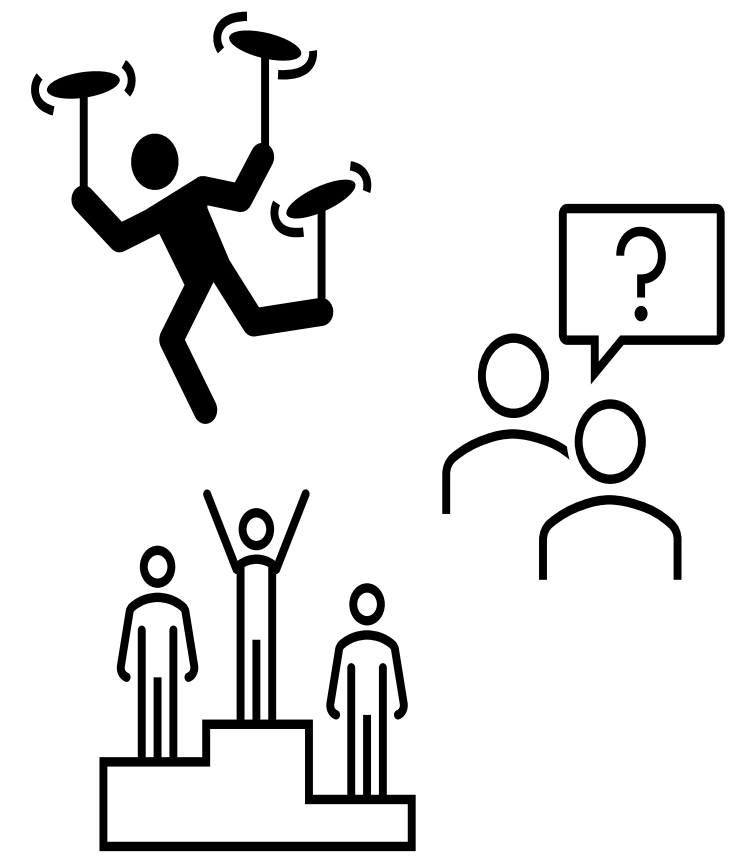

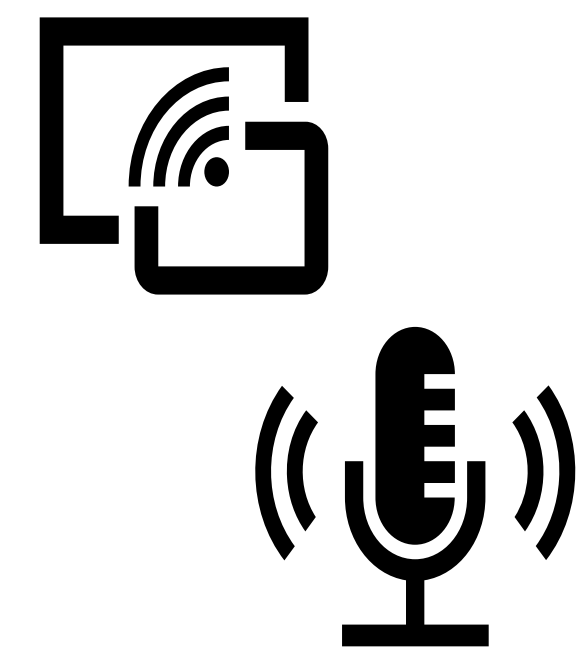

Recommendation<br>Find networks not just CAPs	Infographic<br>Venue use and circumnavigating challenges	Infographic<br>What is SMI, adaptions to PA sessions for CAPs	Awareness video of case studies + infographic applied aspects of delivering intervention at venue
PAC training update delivered 10th June and training slides updated ✓	Group A ✓	Group B ✓	Requires more time

What happens next?

1

- The research team will make these infographics and if possible, create the video
- You will be asked to review and comment on these drafts (November)

2

- The research team will make necessary amendments and produce final versions for use within the SPACES intervention and make publicly available on the SPACES website.

3

- The research team will write up and publish the process all of us have followed.
- You will be listed as a contributor, if you are happy for us to do so.

Co-SPACES

Thank you for all your time, effort, and invaluable input.
