# Supplementary material for: Bridging the gap: co-producing resources for NHS mental health trusts and community providers to support people with severe mental illness live active lives in their community
Source: BMC Public Health. 2026 Jun 16;26:2418. doi: 10.1186/s12889-026-27936-7 (PMC13501637; doi:10.1186/s12889-026-27936-7)
Supplement: Supplementary file 4 [file 12889_2026_27936_MOESM7_ESM.pdf]

Creating welcoming and safe activity spaces for people with severe mental illness

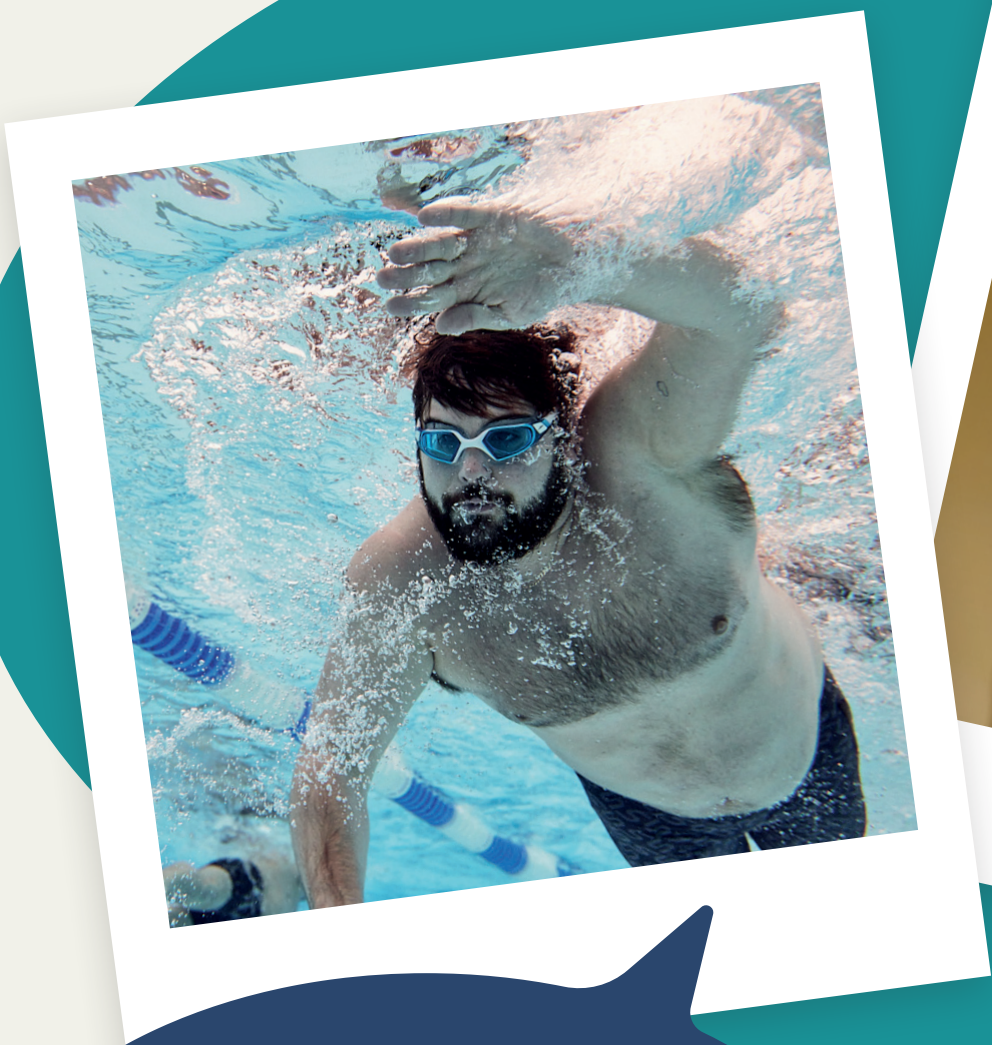

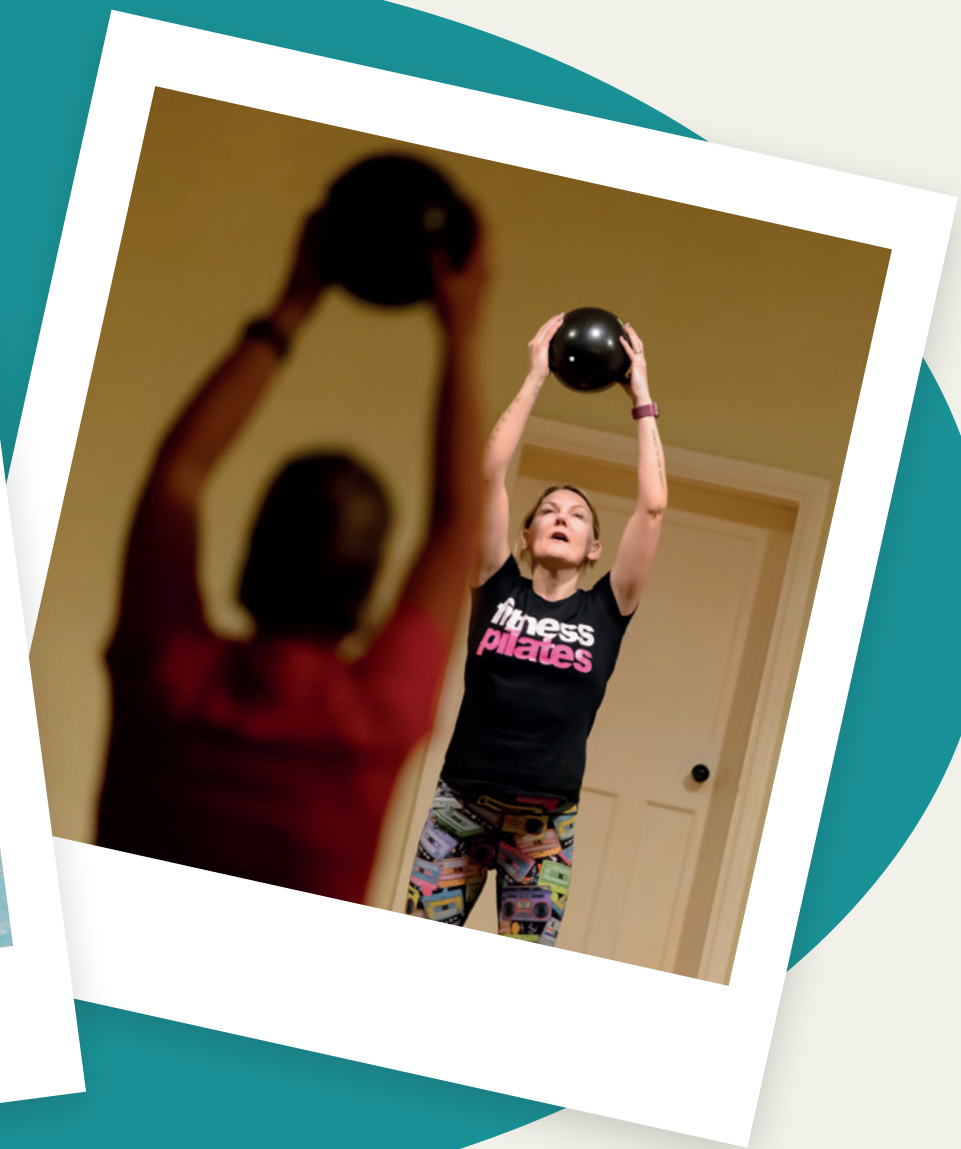

"I know I have some needs, but basically, I just want to be treated like everyone else."

Simon

SPACES
Supporting Physical Activity through Co-production

Where possible, all activity spaces should be safe and welcoming for those with severe mental illness, so that everyone feels confident and comfortable to be active in community environments.

This guide is for anyone - community members, instructors, staff - who wants to help create spaces for physical activity that feel safe, inclusive and welcoming for people living with severe mental illness.

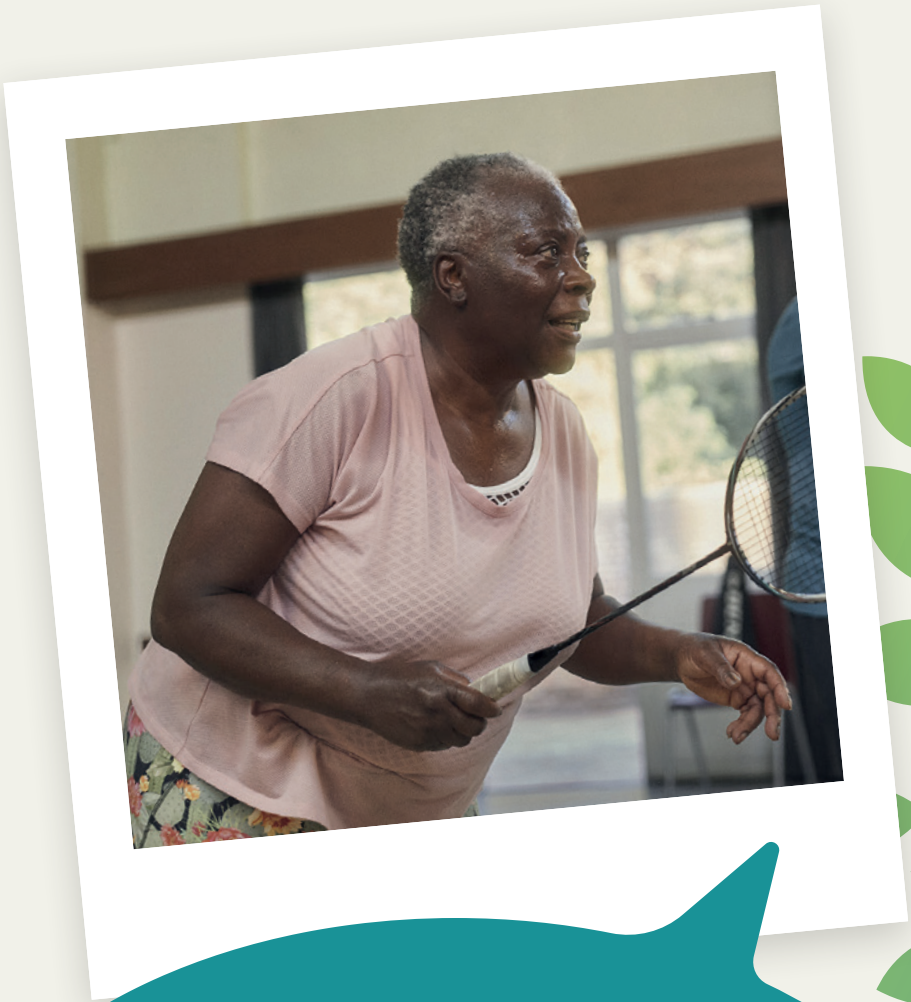

"We're all human, we're all individuals, many of us have regular jobs and families, and our conditions are well managed. Mental illness is not what you read in the news."

Dianne

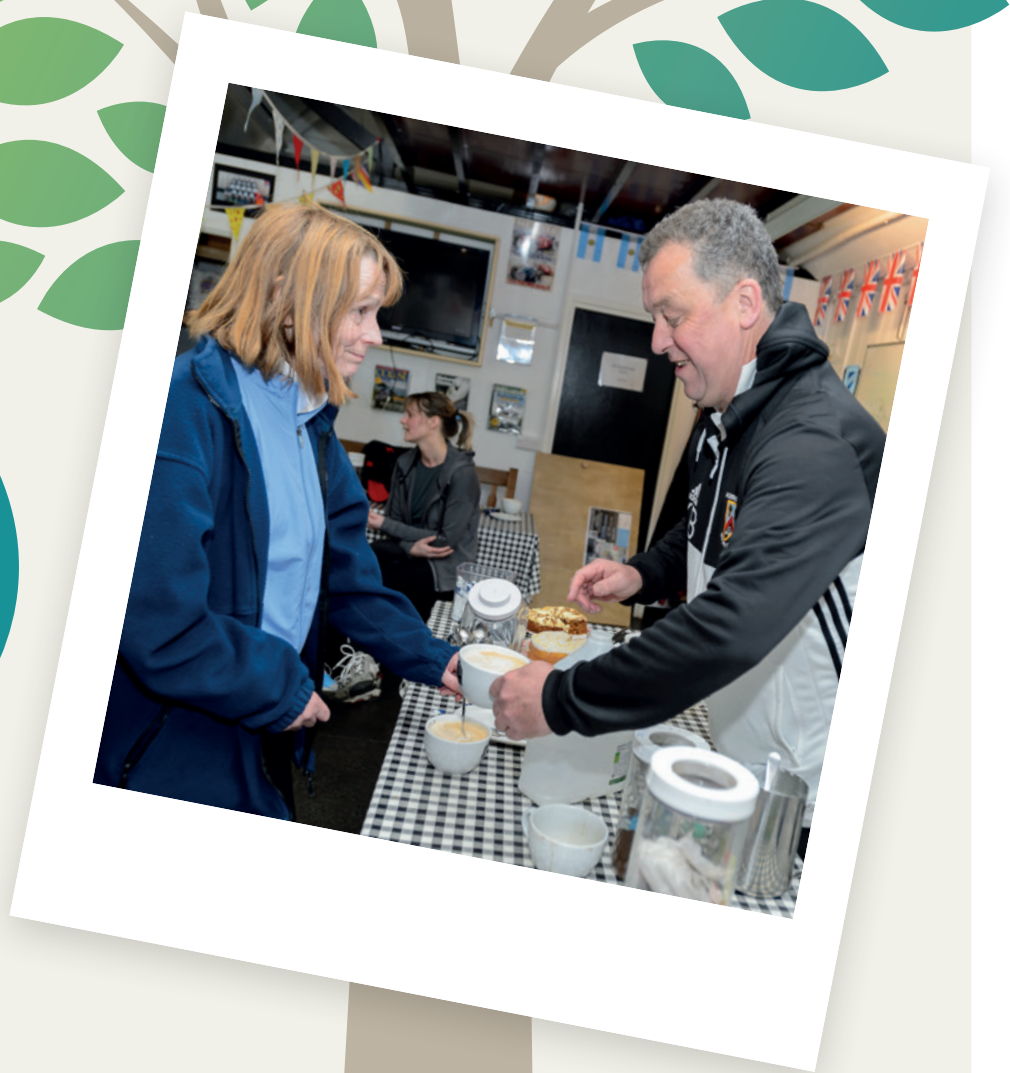

Some useful information

What is severe mental illness?

Severe mental illness (SMI) means different things to different people, but in the UK, it is officially defined as being diagnosed with conditions like schizophrenia, schizoaffective disorder, psychosis and bipolar disorder. While it's different for everyone, these conditions can lead to people experiencing hallucinations or delusional thoughts, mood shifts, social withdrawal, lack of motivation and paranoia. Being diagnosed with SMI usually means that people experience things differently and this can make life more difficult.

What severe mental illness is not

This definition should not be seen to imply that other diagnoses are not serious or severe or that they do not carry any associated physical health risks. This definition is used solely to align this booklet with national guidance for physical health checks.

Why is physical activity so important for people with severe mental illness?

Physical activity benefits everyone's wellbeing, but for many people living with severe mental illness, it can offer something more profound. Beyond the physical and mental health benefits, it can create moments of calm, connection and belonging, improved quality of life, and improved sleep.

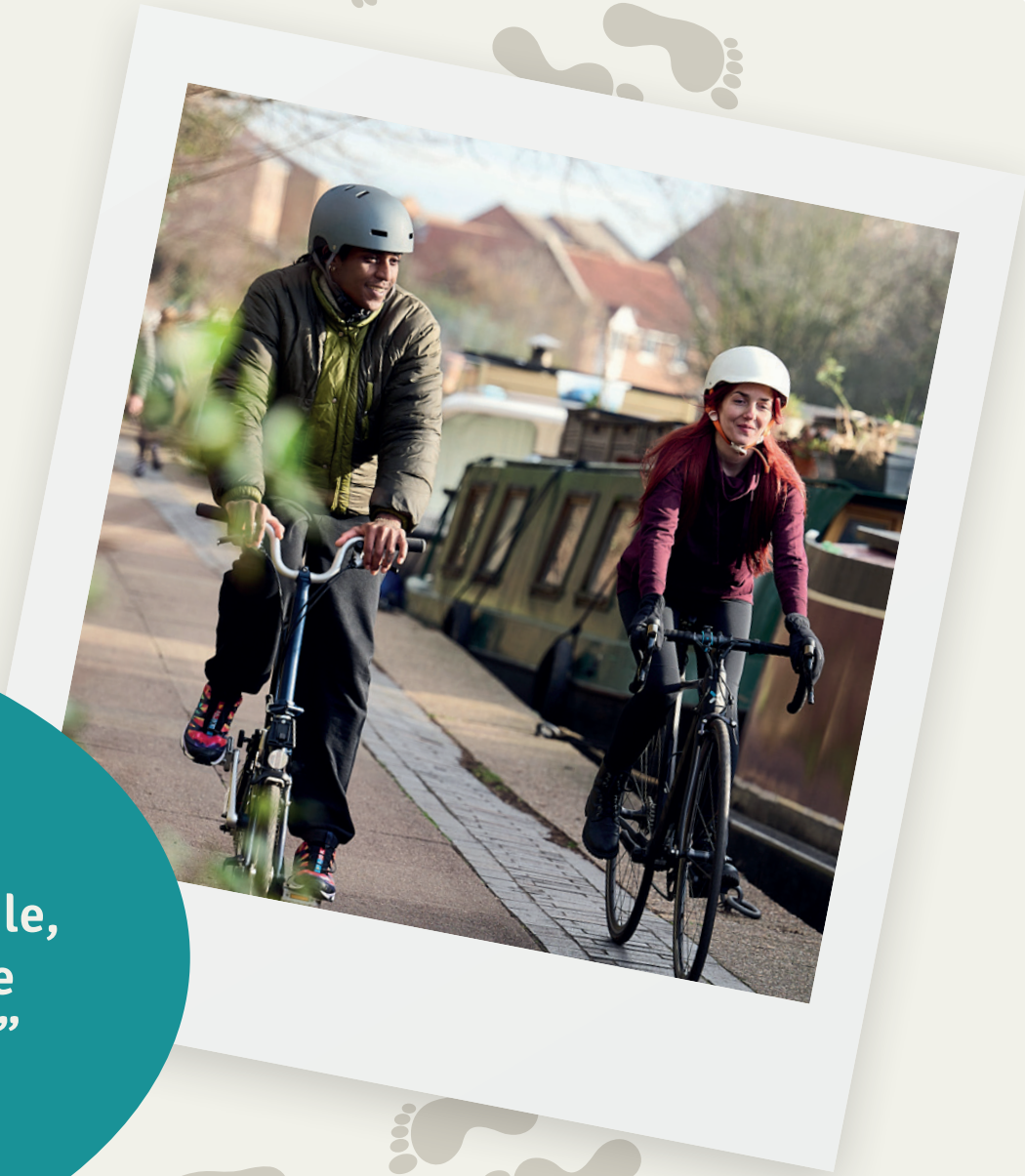

"Physical activity makes the voices go quiet and just for a while, lets me feel like everybody else enjoying their activity session."

Ravi

Accessing physical activity in the community

Let's walk alongside Claire

Claire, who lives with schizophrenia, shares what helped her feel safe and supported in community physical activity spaces. Accessing physical activities in the community can be a challenging journey.

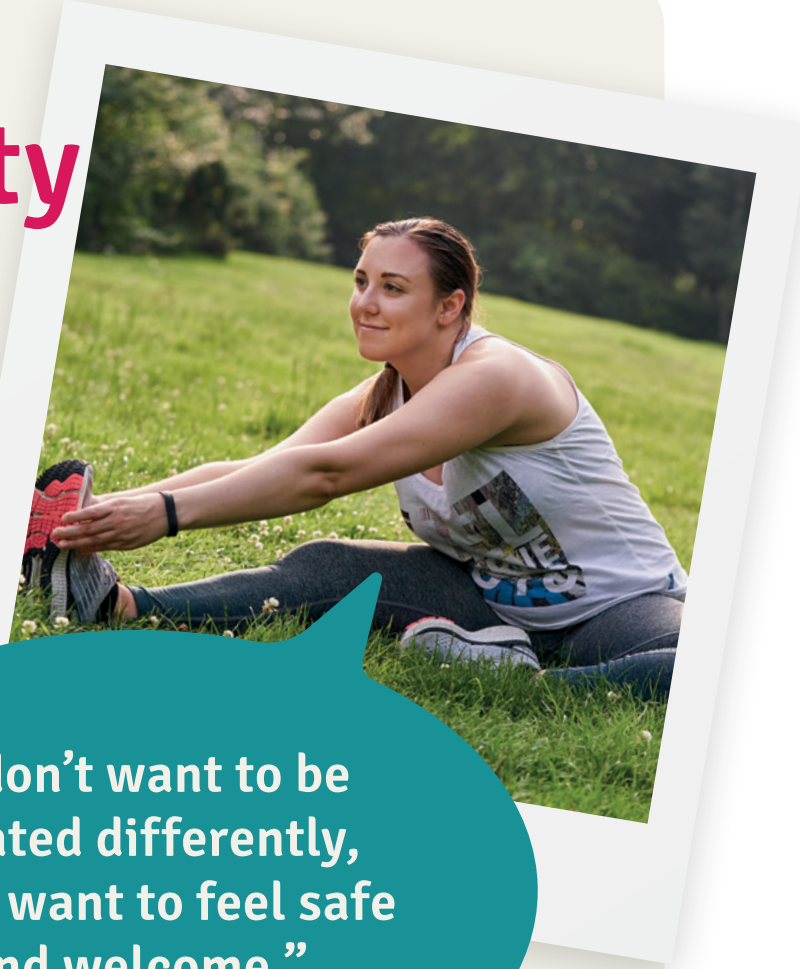

> "I don't want to be treated differently, I just want to feel safe and welcome."
>
> Claire

What it feels like to experience psychosis?

- I might hear voices
- My medication makes me tired
- I worry about what people might think of me
- I worry about the stigma associated with my condition

These feelings make entering community environments very intimidating and scary.

- I may come with a friend, relative, or carer
- I might need a break / more time during a session
- I might need additional demonstrations and instructions
- I might need support with how to adjust the exercises

When entering a facility…

Having staff with knowledge of how to create welcoming, non-judgemental physical activity spaces is important for everyone, including receptionists, cleaners, volunteers, café workers and instructors.

What support helps me feel a community space is a safe and welcoming place

- A warm, respectful welcome can make all the difference.
- Let a friend, family member or carer attend sessions alongside someone with SMI
- Offer a flexible approach and plenty of understanding
- Use inclusive, non-stigmatising language – words matter
- Consider visible signs of mental health awareness, such as a poster to show support

During the activity…

What actions might help me cope with my condition during the activity?

- Understand that every individual is different. I might need:
  - Extra breaks or more time
  - Additional instructions or demonstrations
  - Quiet spaces to pause and regroup
- Be aware of the side effects of medication (e.g., tiredness)
- The instructor's previous knowledge of my physical activity readiness may also be useful, although some people with SMI may not like to fill out forms like the PAR-Q
- Celebrating all achievements – for me, turning up is a huge achievement
- Offer continuity – consistent instructors and sessions times help build trust
- Co-create spaces and offer sessions run with peer leaders or mental health champions
- A safe space to relax and/or socialise after the activity would be helpful
- Clear instructions of where to go and an idea of the room
- Reasonable group sizes in classes, around 8 seems to work well
- Maintain a calm, sensory-considerate environment (no loud music or flashing lights)
- Encourage relationship-building between participants and community venue users – connection matters.
- Appropriate timing of any sessions – early afternoons help with medication

Community Providers

If you need more information about supporting those with SMI to engage in physical activities you offer, please see further guidance from **NHS England** and **Open Mental Health**.

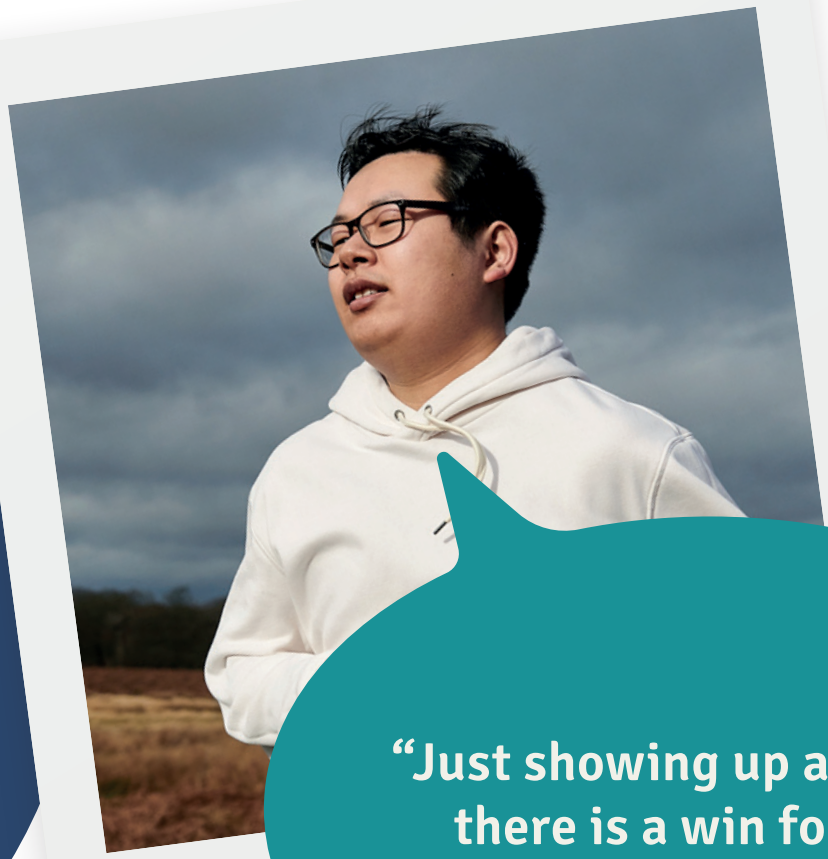

"Just showing up and being there is a win for me."

Adrian

Creating a safe activity space
Factors to consider – the 3 Ps

People

- ☐ Welcoming, friendly greetings upon arrival
- ☐ Optional pre-session introductions or inductions to help ease anxiety and build trust
- ☐ Staffing levels appropriate to activity and group size
- ☐ All staff are informed of SMI awareness and trauma-informed care
- ☐ Delivery staff are aware of the possibility of mobility issues
- ☐ Site induction offered, including toilets, lockers, exits and fire escapes
- ☐ Peer support presence or wellbeing champions
- ☐ Participants have the option to bring a companion for support; friend, family, care worker

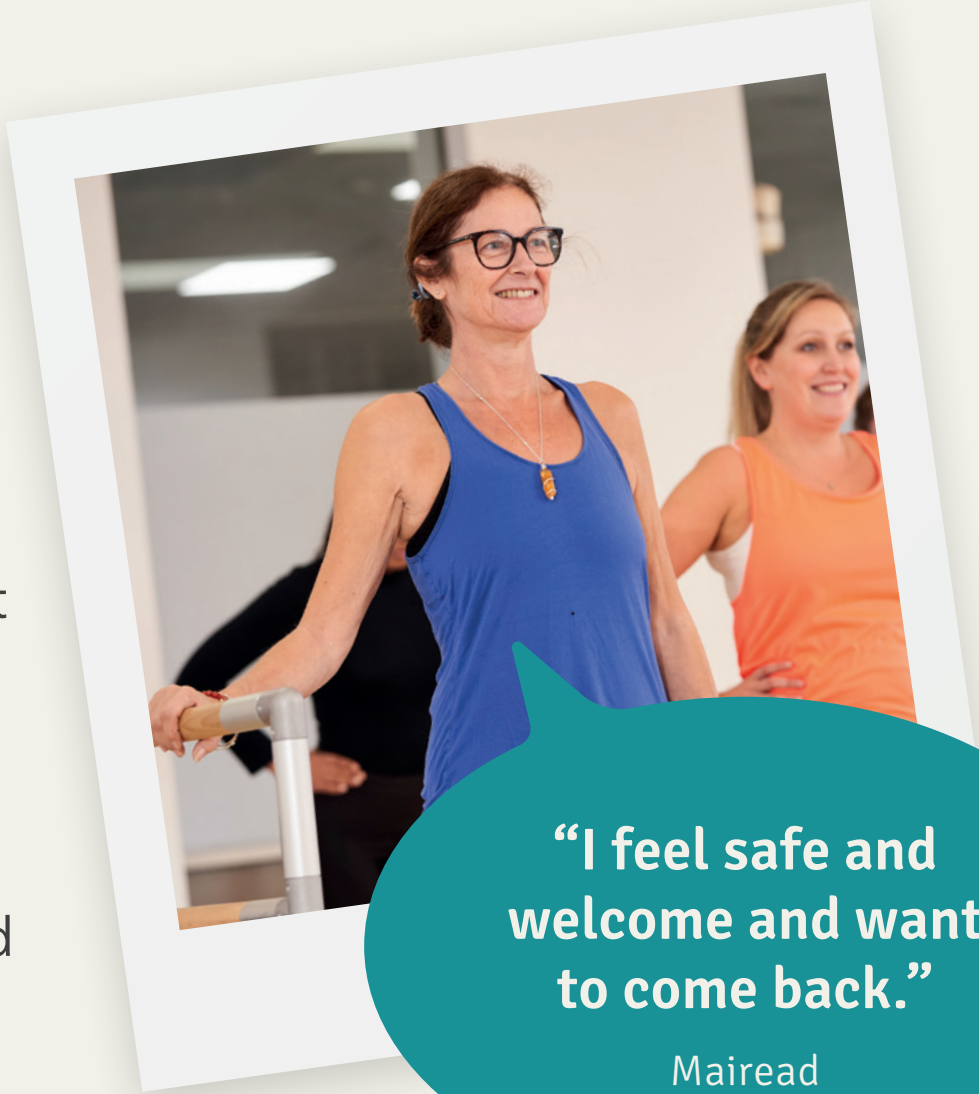

"I feel safe and welcome and want to come back."

Mairead

Find case studies of other people's real life journeys

“I love how we’ve made our community venue truly inclusive.”

Becky

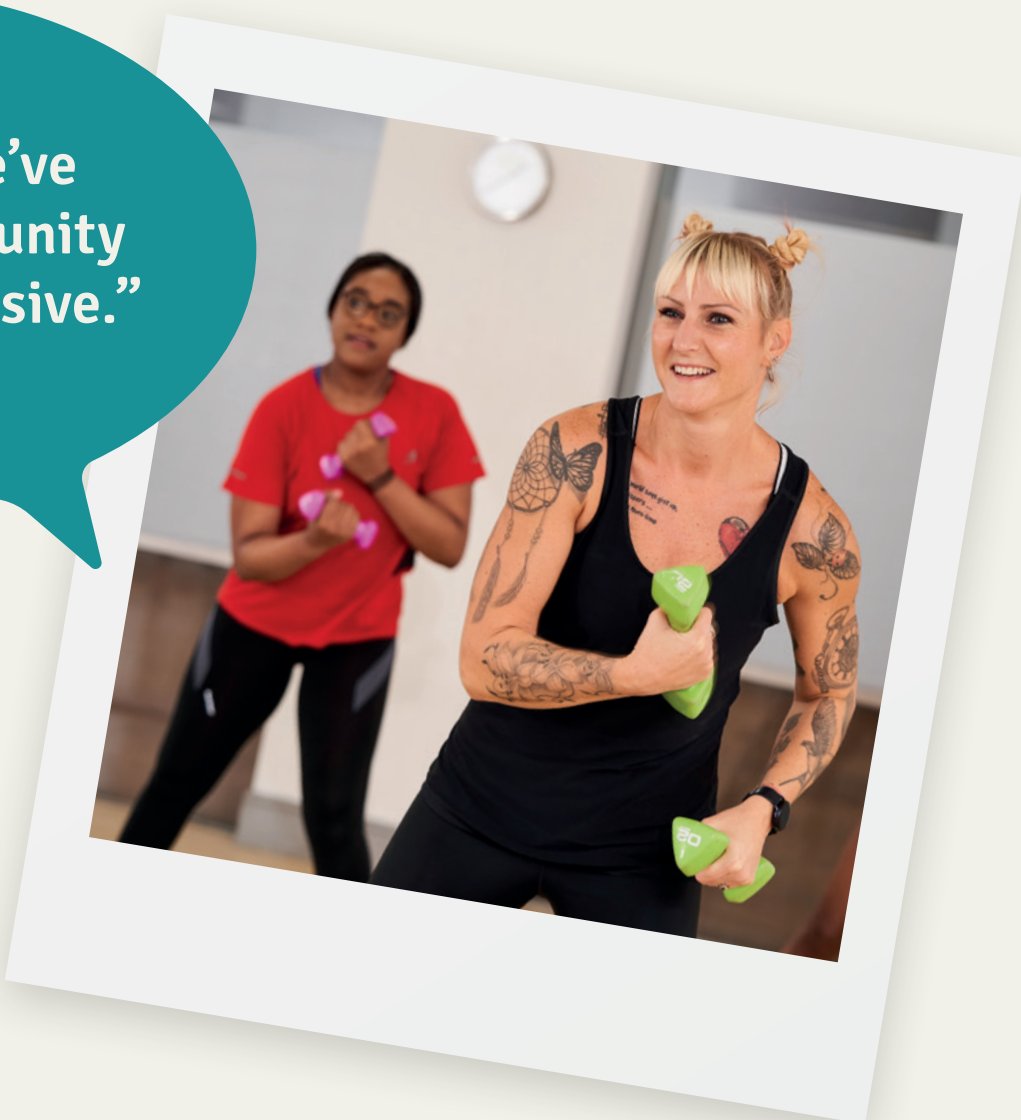

Place

- [ ] An open and well-lit space
- [ ] Calming atmosphere with minimal sensory overload
- [ ] Quiet rooms or spaces to decompress
- [ ] Clear signage and easy to navigate entry points with discrete options
- [ ] Warm, clean and well-maintained facilities
- [ ] Clearly marked café area or drinks/refreshments that are affordable
- [ ] Defined outdoor areas for walking or gentle activity
- [ ] Transport access and parking options
- [ ] Video tour and images of venue
- [ ] Smoking / vaping areas clearly defined

Provision

- [ ] There is no one size fits all, ensure a personal approach is taken
- [ ] Low intensity session options
- [ ] Fun, relaxed, supportive atmosphere
- [ ] Cater for limited mobility
- [ ] Beginner sessions available
- [ ] Variety of activity options
- [ ] Include a social element

You may want to check client’s readiness to be physically active using this Physical Activity Readiness Questionnaire (PAR-Q)

Some useful organisations in your area:

This guide, including the language and contents, was agreed and has been co-created by people living with schizophrenia, schizoaffective disorder, psychosis and bipolar disorder, alongside NHS professionals and community activity leaders. Together, we can design spaces that feel safe, affirming and inclusive.

Images used © Sport England. All Rights Reserved.

NHS Sheffield Health Partnership University NHS Foundation Trust | University of Leeds | University of Sheffield | York St John University Est. 1841 | Prifysgol Bangor University | Sheffield Hallam University | Sport for Confidence | NHS Tees, Esk and Wear Valleys NHS Foundation Trust
