# Supplementary material for: Bridging the gap: co-producing resources for NHS mental health trusts and community providers to support people with severe mental illness live active lives in their community
Source: BMC Public Health. 2026 Jun 16;26:2418. doi: 10.1186/s12889-026-27936-7 (PMC13501637; doi:10.1186/s12889-026-27936-7)
Supplement: Supplementary file 5 [file 12889_2026_27936_MOESM8_ESM.pdf]

Sheffield Hallam University

Consent

Understanding community-based physical activity providers' capacity, attitudes, and infrastructure to support people with Severe Mental Illness (SMI)

Thank you for agreeing to take part in this survey.

In this survey, the Co-SPACES (**Co**mmunity - **S**upporting **P**hysical **A**ctivity through **C**o-Production for p**e**ople with **S**evere Mental Illness) research team are wanting to understand community physical activity providers' perspectives of engaging and including people with severe mental illness in physical activity.

The survey normally takes around 10 minutes to complete, but please take as much time as you need. You can choose not to answer some questions by moving onto the next question or selecting 'prefer not to say' and you can close the survey at any time by closing your browser. Though if you do this, the information you provide up to that point will be saved and used in the findings.

For more information about this survey, such as how we will use your data, please read a more detailed participant information sheet via this link: Co-SPACES participant information sheet For any further questions or concerns you may have about this survey, please contact the research team via email.
Email: co-spacesproject@shu.ac.uk

SPACES is a programme funded by the National Institute for Health and Care Research (NIHR) to design and test a physical activity intervention for those with severe mental illness (SMI; such as schizophrenia, schizoaffective disorder, and bipolar disorder, not including primary diagnosis of anxiety or depression) to be delivered within the NHS. As part of that programme, it was evident a community component was key to support sustainable physical activity. Community SPACES (Co-SPACES) is a spin off programme aiming to understand and support the links between the NHS and community providers in the provision of physical activity for people with SMI. Purpose of this questionnaire: To gain an understanding of the barriers to engaging community-based physical activity providers in the management of SMI in the NHS. To identify what support community-based physical activity providers need to support people with SMI. To inform training resources that can be shared with community-based physical activity providers to help them support people with SMI

Giving consent to take part in this survey

By taking part in this survey, you understand:

- That none of your data will be used, stored, shared or reported in a way that can identify you/your organisation.
- The answers you provide will be stored securely on a University storage network.
- That the findings and your words may be quoted in publications, reports, web pages, and other research outputs.
- That you voluntarily agree to take part.

Consent

- ◯ I consent to participate in this survey
- ◯ I DO NOT consent to participate in this survey

Governance and Leadership

Your organisation

What type of organisation do you represent?

If more than one, please select the one that represents your primary organisation.

All your answers in this survey should reflect the answer you provide here.

- ○ Sole trader (e.g., Yoga instructor, personal trainer)
- ○ Fitness or leisure operator
- ○ Active Partnership
- ○ Sports club
- ○ [ ] Other - please state
- ○ Prefer not to say

At how many sites does your organisation operate from?

- ○ 1
- ○ 2
- ○ 3
- ○ 4
- ○ 5 or more
- ○ Unsure
- ○ Not applicable

Please indicate if your responses to this survey are for:

- ○ Whole Organisation (e.g., a governing body or national gym operator)
- ○ Specific region (e.g., leisure provider within one region with multiple sites or a council sport and leisure service)
- ○ Specific centre, site, or club (e.g., a specific leisure centre or an exercise class)
- ○ Not applicable

Where does your organisation operate?

Select all that apply

- ☐ North West England
- ☐ North East England
- ☐ Yorkshire
- ☐ West Midlands
- ☐ East Midlands
- ☐ South West England
- ☐ South East England
- ☐ East Anglia
- ☐ London
- ☐ Scotland
- ☐ Wales
- ☐ Northern Ireland
- ☐ Republic of Ireland

- [ ] Prefer not to say
- [ ] ______ Other

What is the size of your organisation?

- ○ Micro enterprise (fewer than 10 employees)
- ○ Small enterprise (10-49 employees)
- ○ Medium-sized enterprise (50-249 employees)
- ○ Large enterprise (250 or more employees)

What is your role within your organisation?

Select all that apply

- [ ] Management
- [ ] Deliver physical activity
- [ ] Exercise referral manager / coordinator
- [ ] Exercise referral instructor
- [ ] Committee member
- [ ] Front desk staff
- [ ] Receptionist
- [ ] Lifeguard
- [ ] Maintenance staff
- [ ] Healthcare professional
- [ ] Other employee
- [ ] Sole trader

- ☐ Club member
- ☐ Volunteer
- ☐ [ ] Other - please state

What is the focus of your organisation's provision?

- ○ General public
- ○ Targeted groups (e.g., elderly groups, dementia, disability)
- ○ Both

What type of physical activity provider are you?

Select all that apply

- ☐ Gym
- ☐ Leisure centre
- ☐ Fitness centre
- ☐ Sports club
- ☐ Community sports venue
- ☐ Swimming pool
- ☐ Outdoor sports complex
- ☐ Exercise class
- ☐ Personal trainer
- ☐ Boutique gym / franchise
- ☐ Green or blue activity provider
- ☐ Sports venue

- [ ] ______ Other
- [ ] Prefer not to say

How does your organisation provide physical activity?

Select all that apply

- [ ] Group classes (e.g., Zumba, Body Pump, Spin classes, yoga)
- [ ] Personal training
- [ ] Non class based activities such as weights/public swim sessions
- [ ] Sporting clubs and activities (i.e., training and competition)
- [ ] One-off sporting or activity events
- [ ] Programmes targeted at a specific group (e.g., Dementia friendly or post natal group)
- [ ] Green or blue outdoor activity
- [ ] ______ Other (please specify)
- [ ] Unsure
- [ ] Prefer not to say

Please list the different activities / sports you provide

______

Is your organisation a 'Disability Confident Employer'?

- ◯ Yes
- ◯ No
- ◯ Not sure
- ◯ Prefer not to say

Please select which level of Disability Confident Employer certificate is obtained.

- ◯ 1
- ◯ 2
- ◯ 3
- ◯ Not sure
- ◯ Prefer not to say

Does your organisation have initiatives that target particular groups, communities or characteristics?

Select all that apply

- ☐ Long term health condition (e.g., heart conditions, dementia)
- ☐ Physical disabilities (e.g., wheelchair users)
- ☐ Mental health (e.g., wellbeing / mental illness)
- ☐ Learning disabilities (e.g., autism, down's syndrome)
- ☐ Ethnic or cultural diversity

- ☐ Religion/belief groups
- ☐ LGBTQ+
- ☐ Pregnancy/Maternity
- ☐ Older adults
- ☐ Children and young people
- ☐ Men and/or boys only groups
- ☐ Women and/or girls only group
- ☐ Other (please specify)
- ☐ Not sure
- ☐ Prefer not to say

How many people typically attend a physical activity session that your organisation offers.

- ○ 1
- ○ 2-5
- ○ 6-10
- ○ 11-20
- ○ 20-49
- ○ 50+
- ○ Not sure
- ○ Prefer not to say

Is paid membership of the organisation required?

- ○ No

- Yes
- Yes, but we offer discounted rates. Please state who is eligible for discounted rates
- Pay as you go
- Not applicable

If membership is required, please specify the cost per annum (select all that apply if multiple options available)

- Less than £50
- £51 to £100
- £101 to £150
- £151 to £200
- £201 to £250
- £251 to £300
- £301 to £400
- £401 to £500
- £500 or more
- Not applicable

Does your organisation have any of the following roles?

Select all that apply

- Welfare/safeguarding

- ☐ Diversity and inclusion
- ☐ First aider
- ☐ Mental health first aider
- ☐ Not sure
- ☐ Prefer not to say

Barriers to supporting SMI

Barriers to supporting SMI

To the best of your knowledge, please answer the following questions regarding your organisation's physical activity provision

Instructors' physical activity training / qualifications prepares them to engage with people who have severe mental illness

- ○ Strongly agree
- ○ Agree
- ○ Neither agree nor disagree
- ○ Disagree
- ○ Strongly disagree

Staff at my organisation have undertaken additional courses related to mental health or severe mental illness

- Definitely yes
- I think so
- Not sure
- I do not think so
- Definitely not

If known, please specify the names of these courses

My **organisation** would **need** to make changes in order to engage with people who have severe mental illness

- Definitely yes
- I think so
- Not sure
- I do not think so
- Definitely no

My **organisation** is **able** to change what we offer to engage with people who have severe mental illness

- Definitely yes
- I think so
- Not sure
- I do not think so
- Definitely no

Please explain what may need to change in order to engage with people who have severe mental illness (if applicable)

My **organisation** has engaged with people who have severe mental illness in the past

- Definitely yes
- I think so
- Not sure
- I do not think so
- Definitely not

Instructors in my organisation have personal experience of living with, caring for, or knowing someone with severe mental illness

- Definitely yes
- I think so
- Not sure
- I do not think so
- Definitely not

Capability, Opportunity, Motivation

Capability, Opportunity, Motivation

To the best of your knowledge, please answer the following questions regarding your organisation's physical activity provision

Instructors in my organisation feel confident in their ability to provide physical activity to a client who has severe mental illness.

- Strongly agree

- Agree
- Neither agree nor disagree
- Disagree
- Strongly disagree

Instructors in my organisation have sufficient skills and knowledge to provide physical activity to people who have severe mental illness

- Strongly agree
- Agree
- Neither agree nor disagree
- Disagree
- Strongly disagree

Staff in my organisation have sufficient support from the organisation to engage with people who have severe mental illness.

- Strongly agree
- Agree
- Neither agree nor disagree
- Disagree
- Strongly disagree

Staff in my organisation are personally willing to engage people with severe mental illness

- Strongly agree
- Agree
- Neither agree nor disagree
- Disagree
- Strongly disagree

My **organisation** is willing to support people with severe mental illness (e.g., it is an organisational aim)

- Strongly agree
- Agree
- Neither agree nor disagree
- Disagree
- Strongly disagree

Future outputs

To the best of your knowledge, please answer the following questions regarding your organisation's physical activity provision

My **organisation** would benefit from training and guidance to help us engage with people who have severe mental illness

- ○ Strongly agree
- ○ Agree
- ○ Neither agree nor disagree
- ○ Disagree
- ○ Strongly disagree

What would this training and guidance need to cover? Please highlight all things that come to mind.

What training methods would work best for you / your **organisation**?

Select all that apply

- ☐ Brief online training
- ☐ Intensive online training
- ☐ Online resources portal
- ☐ In person practical training course
- ☐ Information guides/booklets

- [ ] [ ] Other, please specify
- [ ] Not applicable

Powered by Qualtrics
