# Supplementary material for: Bridging the gap: co-producing resources for NHS mental health trusts and community providers to support people with severe mental illness live active lives in their community
Source: BMC Public Health. 2026 Jun 16;26:2418. doi: 10.1186/s12889-026-27936-7 (PMC13501637; doi:10.1186/s12889-026-27936-7)
Supplement: Supplementary file 6 [file 12889_2026_27936_MOESM1_ESM.docx]

GRIPP2-Short Form Checklist for the Reporting of Patient and Public Involvement (PPI) in research

| Section and topic | Item | Reported on page No |
| --- | --- | --- |
| 1: Aim | *Report the aim of PPI in the study*  The aim was to ensure to ensure end-users experiences were embedded in the co-production of resources. | Page 7,  Paragraph 2,  Line 139-141 |
| 2: Methods | *Provide a clear description of the methods used for PPI in the study*  To ensure that the views of people with lived experience were included in the process we shared our findings with the PPIE group to obtain their feedback and suggestions (Define phase). People with lived experience of SMI (who were not members of the PPIE group) took part in the stakeholder workshop (Develop phase) and we shared the resources with the PPIE group and asked for suggestions for improvement before finalising (Deliver phase)  The survey was designed through consultation with key stakeholders, including our partner organisation Sport for Confidence and public co-applicant  Workshop attendees were purposefully recruited from PACs and community providers identified during the interviews and through additional stakeholders and partner organisations, ensuring representation from people living with SMI, community providers, and professionals. | Page 11, Paragraph 1, Lines 217-219,  Page 11, Paragraph 4, Lines 235-239 |
| 3: Study results | *Outcomes—Report the results of PPI in the study, including both positive and negative outcomes*  PPI contributed to the study in several ways, including:   - The public co-applicant provided input into the development of the survey of community physical activity providers - PPIE colleagues were invited to attend the workshops and contribute to the development of the resources. - Workshop participants and the SPACES PPIE group were invited to review and give feedback on the first iterations of the booklet before being revised and finalised. | Page 10, Paragraph 1 Lines 217-219  Page 11, Paragraph 4, Lines 229-233  Page 21, Paragraph 1, Lines 442-445 |
| 4: Discussion and conclusions | *Outcomes—Comment on the extent to which PPI influenced*  *the study overall. Describe positive and negative effects*  In summary, co-producing solutions with service users, community providers, and professionals resulted in practical resources that address the gap between clinical support and community-based physical activity. These resources have the potential to improve the inclusivity of community sport and exercise environments for people with SMI, which may support longer-term lifestyle change beyond short-term interventions. | Page 25, Paragraph 3, Line 538-543 |
| 5: Reflections | *Comment critically on the study, reflecting on the things that went well and those that did not, so others can learn from this experience*  The major strength of Co-SPACES is its co- production methodology, embedding the perspective of multiple stakeholders into the support resources produced (lived experience service users, community providers, NHS professionals). The study’s use of multiple data sources across stakeholder groups strengthens the credibility of the findings. And the commitment to lived experience input adds authenticity and impact to the resources produced.  This project underscores the value of co-production with end users in developing practical solutions to real-world challenges. By actively involving lived experience service users, community providers and NHS professionals we created resources that are grounded in real-world needs. | Page 25, Paragraph 3, Line 538-543  Page 26, Paragraph 2, Line 560-563 |
