# Supplementary material for: Bridging the gap: co-producing resources for NHS mental health trusts and community providers to support people with severe mental illness live active lives in their community
Source: BMC Public Health. 2026 Jun 16;26:2418. doi: 10.1186/s12889-026-27936-7 (PMC13501637; doi:10.1186/s12889-026-27936-7)
Supplement: Supplementary file 7 [file 12889_2026_27936_MOESM3_ESM.docx]

Co-SPACES WS1 Interviews

Discover and Define Interview Schedule

**Phase 1- PACs and NHS-based researchers**

Objectives:

**To conduct semi-structured interviews with a purposive sample of people involved in the delivery of the SPACES intervention (SPACES PACs and NHS-based researchers) to gain an in-depth understanding of the barriers, enablers and support needs of current and future PACs in identifying and engaging community-based physical activity providers in the SPACES intervention.**

*Remind participant about the purpose of the research

*Ask if they are happy with the interview being recorded

*Turn on recording

*Read out consent statements

*stop consent recording*

*start interview recording*

*Start interview

Definition of what we mean by SMI

Severe mental illness means different things to different people. But in the UK, it’s officially defined as being diagnosed with conditions like schizophrenia and bipolar disorder.

While it’s different for everyone, these conditions can lead to people living with psychosis and experiencing hallucinations (seeing or hearing things that most people don’t) or delusional thoughts (strongly believing something to be true when most people believe it is not).

Being diagnosed with a SMI usually means that people experience things differently and this can make life more difficult.

Opening question– *what involvement have you had or plan to have with SPACES?*

Purpose: understand their experience of referring people with SMI to community-based physical activity providers (what’s happening / what is our starting point – acknowledging that people have different experiences of doing this)

*Can you tell me about your experience of referring people with SMI to community-based physical activity providers (what did it involve?)*

*How did it go? (for you and the participant/person with SMI)*

Explore whether they have encouraged self-referrals to community-based physical activity providers (to be asked to those who have not taken part in SPACES Feasibility Study)

*What does referral to community-based PA opportunities involve?* (How do you go about it)

*Follow-up*: *it is a one-off thing, or do you follow-up to see if it was effective?*

Purpose: to explore the acceptability of PACs making referrals to existing physical activity opportunities within the local community

*How do you feel about referring SPACES participants/people with SMI to community-based physical activity providers?*

*How important is it that SPACES participants/people with SMI engage with community-based physical activities?*

*Why is it important / not important?*

Purpose: to establish the extent to which they feel **competent** to support people with SMI to take up PA opportunities in the community

*How do you feel about your ability to identify appropriate or suitable community-based physical activity providers*

*What would need to happen to make you feel better able to doing this?*

Purpose: Establish the extent to which they feel **confident** to support people with SMI to take up PA opportunities in the community

*How confident are you about identifying community-based physical activity providers?*

*How confident are you in referring SPACES participants/people with SMI to community-based physical activity providers?*

*[if applicable] What helps you feel confident to do this?*

*[if applicable] What would need to happen to make you feel more confident doing this?*

*Explore any training or additional support needs to help SPACES participants/people with SMI*

*to take up PA in their local community*

Purpose: Establish the extent to which they feel and **comfortable** to support people with SMI to take up PA opportunities in the community

*How comfortable do you feel referring SPACES participants/people with SMI to community-based physical activities?*

*[if applicable] What helps you feel comfortable doing this?*

*[if applicable] What would need to happen to make you feel more comfortable doing this?*

*What would you like to know about the community-based PA providers to feel comfortable about referring SPACES participants/people with SMI to them (what are you looking for in a provider?)*

*[if applicable] What support would community-based PA providers benefit from to cater for the needs of people with SMI?*

[if not already explored above] Purpose: To identify any *additional* barriers that prevent them from supporting SPACES participants/people with SMI to take up community-based physical activity opportunities

- SMI-related barriers
- Practitioner-related barriers
- Organisational barriers
  - Explore if workload is a barrier / sufficient time
  - Explore if it’s a priority for organisation
- Provider-related barriers (e.g., Availability and access barriers)

“About you” – please only share this information if you feel comfortable.

- What is your gender?
- What is your ethnicity?
- What is your job role?
- How many years of experience with SMI?

**Phase 2- community-based physical activity providers**

Objectives:

**To conduct semi-structured interviews with community-based physical activity providers to understand their knowledge of the needs of people with SMI, as well as their capacity and willingness to support SPACES participants (people who are living with SMI) in their physical activity provision.**

*Remind participant about the purpose of the research

*Ask if they are happy with the interview being recorded

*Turn on recording

*Read out consent statements

*stop consent recording*

*start interview recording*

*Start interview

Definition of what we mean by SMI

Severe mental illness means different things to different people. But in the UK, it’s officially defined as being diagnosed with conditions like schizophrenia and bipolar disorder.

While it’s different for everyone, these conditions can lead to people living with psychosis and experiencing hallucinations (seeing or hearing things that most people don’t) or delusional thoughts (strongly believing something to be true when most people believe it is not).

Being diagnosed with a SMI usually means that people experience things differently and this can make life more difficult.

Purpose: **Experience** of engaging with people who are living with SMI (or mental illnesses more generally)

*Has your organisation ever engaged with people living with SMI before? (if yes, tell us about this – If no, why do you think this is?)*

*Prompts – what worked? What didn’t work? What did you learn?*

*Prompts – how important would it be for your organisation to engage with people living with SMI? Would it be considered a priority? Why / why not?*

*NOTE – if getting nothing about SMI – explore other mental illnesses*

[For those who have been involved in SPACES Feasibility Trial] – what was your experience of engaging with SPACES participants?

*How were you approached?*

*What worked? What didn’t work? What did you learn from this experience?*

*How did you find the session? How could it be improved?*

Purpose: Understand how **willing** and **confident** they are in engaging with people with SMI

*Would your organisation be willing to broaden your offer to cater to SPACES participants / people with SMI?*

*Prompts – organisation priorities*

*Would your organisation / your colleagues feel confident to cater for the needs of SPACES participants / people with SMI?*

Purpose: Understand their **capacity** and **capability** to support SPACES participations / people with SMI

*How suitable do you think your current PA offer is to people living with SMI?*

*Why so?*

*Would your organisation be able to broaden your offer to cater to SPACES participants / people with SMI?*

*What would need to happen for your organisation to be able to cater to the needs of people with SMI?*

*Prompts – training and knowledge / resource / personnel (reliant on volunteer basis? Wellbeing officer? MH officer? Etc)*

Purpose: To explore their support needs and requirements for format of support resources

*What support resources would your organisation benefit from? / format of resources*

“About you” - please only share this information if you feel comfortable.

- What is your gender?
- What is your ethnicity?
- What is your job role?
- How many years experience do you have in physical activity provision?
