# Supplementary material for: Bridging the gap: co-producing resources for NHS mental health trusts and community providers to support people with severe mental illness live active lives in their community
Source: BMC Public Health. 2026 Jun 16;26:2418. doi: 10.1186/s12889-026-27936-7 (PMC13501637; doi:10.1186/s12889-026-27936-7)
Supplement: Supplementary file 8 [file 12889_2026_27936_MOESM4_ESM.docx]

Actions followed to complete framework analysis of Co-SPACES interview transcripts

| ***Step*** | ***Actions taken by the research team*** |
| --- | --- |
| ***Step 1. Data familiarisation*** | TB reviewed all interview transcripts to familiarise herself with the data. Key ideas relating to each set of transcripts were noted. |
| ***Step 2: Framework Identification*** | First cycle coding used the research objectives as a priori codes. Then further codes were generated (Saldana, 2014). TB coded 3 PAC and 3 community provider transcripts. GJ coded one each of these three transcripts. TB and GJ discussed the data and codes generated to identify and agree upon pattern codes and to generate an initial coding framework.    TB applied the agreed initial framework to a further 7 PAC transcripts and 7 community provider transcripts whilst GJ applied the framework to three of each of these. TB and GJ discussed the data and coding framework again to finalise the codes.  TB and GJ discussed the data and coding framework again to finalise the categories and sub-categories. This process was iterative; categories and sub-categories were merged, split and renamed as necessary.    GJ and TB presented themes to the research team for decision on final themes. Discrepancies in coding or theme interpretation were identified and resolved through discussion, leading to consensus. As a result, minor modifications were made to the framework to better capture the nuances in the data. This collaborative review enhanced the trustworthiness of the analysis.    The qualitative software package NVivo 15 (v1.7.1) and Microsoft’s virtual whiteboard were employed to aid this process. |
| ***3: Indexing*** | TB systematically applied the finalised framework to all remaining data. |
| ***4: Charting*** | TB summarised the data in a matrix by transferring verbatim quotes and participant ID numbers into a Word table. Continuous cross-references to the original transcripts ensured that the information represented in the matrix was a true representation of the data set collected. |
| ***5: Mapping and interpretation*** | Codes initially categorised into ‘problems’ and ‘solutions’, ready for the co-production workshops. Then re-categorised to address research objectives. |
